# New Insights into the Ecology and Physiology of *Methanomassiliicoccales* from Terrestrial and Aquatic Environments

**DOI:** 10.3390/microorganisms9010030

**Published:** 2020-12-24

**Authors:** Marc Cozannet, Guillaume Borrel, Erwan Roussel, Yann Moalic, Maxime Allioux, Amandine Sanvoisin, Laurent Toffin, Karine Alain

**Affiliations:** 1 Laboratoire de Microbiologie des Environnements Extrêmes LM2E, Univ Brest, CNRS, IFREMER, IRP 1211 MicrobSea, UMR 6197, IUEM, Rue Dumont d’Urville, F-29280 Plouzané, France; Marc.Cozannet@univ-brest.fr (M.C.); Erwan.Roussel@ifremer.fr (E.R.); Yann.Moalic@univ-brest.fr (Y.M.); Maxime.Allioux@univ-brest.fr (M.A.); Amandine.Sanvoisin@univ-brest.fr (A.S.); Laurent.Toffin@ifremer.fr (L.T.); 2 Unit Evolutionary Biology of the Microbial Cell, Department of Microbiology, Institute Pasteur, 75015 Paris, France; guillaume.borrel@pasteur.fr

**Keywords:** *Methanomassiliicoccales*, cultivation, methyl-compounds, environmental cluster, networks

## Abstract

Members of the archaeal order *Methanomassiliicoccales* are methanogens mainly associated with animal digestive tracts. However, environmental members remain poorly characterized as no representatives not associated with a host have been cultivated so far. In this study, metabarcoding screening combined with quantitative PCR analyses on a collection of diverse non-host-associated environmental samples revealed that *Methanomassiliicoccales* were very scarce in most terrestrial and aquatic ecosystems. Relative abundance of *Methanomassiliicoccales* and substrates/products of methanogenesis were monitored during incubation of environmental slurries. A sediment slurry enriched in *Methanomassiliicoccales* was obtained from a freshwater sample. It allowed the reconstruction of a high-quality metagenome-assembled genome (MAG) corresponding to a new candidate species, for which we propose the name of *Candidatus* ‘Methanomassiliicoccus armoricus MXMAG1’. Comparison of the annotated genome of MXMAG1 with the published genomes and MAGs from *Methanomassiliicoccales* belonging to the 2 known clades (‘free-living’/non-host-associated environmental clade and ‘host-associated’/digestive clade) allowed us to explore the putative physiological traits of *Candidatus* ‘M. armoricus MXMAG1’. As expected, *Ca.* ‘Methanomassiliicoccus armoricus MXMAG1’ had the genetic potential to produce methane by reduction of methyl compounds and dihydrogen oxidation. This MAG encodes for several putative physiological and stress response adaptations, including biosynthesis of trehalose (osmotic and temperature regulations), agmatine production (pH regulation), and arsenic detoxication, by reduction and excretion of arsenite, a mechanism that was only present in the ‘free-living’ clade. An analysis of co-occurrence networks carried out on environmental samples and slurries also showed that *Methanomassiliicoccales* detected in terrestrial and aquatic ecosystems were strongly associated with acetate and dihydrogen producing bacteria commonly found in digestive habitats and which have been reported to form syntrophic relationships with methanogens.

## 1. Introduction

Methanogenic *Archaea* are responsible for production of biogenic methane (CH_4_) on Earth, reported to release one-half of the annual output of this strong greenhouse gas into the atmosphere [1]. Methanogens inhabit a large variety of anoxic terrestrial and aquatic environments, as well as various animal gastrointestinal tracts (GITs) [2]. In these ecosystems, methanogenesis occurs under reduced conditions, in areas where substrates are produced from fermentation (H_2_, CO_2_, acetate, or methylated compounds) or geological processes (H_2_, CO_2_, CO) [3,4,5]. Over the last decade, our knowledge of methanogenesis pathways and the taxa carrying out this process has changed considerably. For example, methoxylated aromatics and short-chain alkanes have been described as potential methanogenesis substrates for newly-described methanogenic lineages [6,7,8]. Furthermore, genome-centric metagenomics studies and cultivation efforts led to the discovery of several new taxa of methanogens phylogenetically distant from the euryarchaeal phylum [9,10,11,12]. Most of these taxa were predicted to perform a methyl-dependent hydrogenotrophic methanogenesis, challenging the paradigm of an ancestral euryarchaeal methanogenesis based on the consumption of H_2_/CO_2_ [8,12,13,14].

The recently described order *Methanomassiliicoccales* has been reported to perform a methyl-dependent hydrogenotrophic methanogenesis (reducing methyl-compounds with H_2_ as electron donor), that involves two types of heterodisulfide reductases, HdrB and HdrD to conserve energy [15,16,17]. In addition, *Methanomassiliicoccales* possess rather rare metabolic properties because they require the 22nd amino acid, pyrrolysine, encoded by an UAG (amber) codon [16].

Based on 16S rRNA and *mcrA* (methyl co-enzyme M subunit A) genes, this order encompasses at least two family-level lineages, respectively, called *Methanomassiliicoccaceae* and *Candidatus* ‘Methanomethylophilaceae’ [18,19,20], frequently associated with the two specific habitats (host-associated habitats versus non-host associated ones), in which they are found and probably reflecting their ecological adaptation [21,22,23,24].

The first cluster, referred to as the ‘host-associated’ clade, corresponding roughly to *Ca.* ‘Methanomethylophilaceae’, is the one that has been the most studied [24,25]. It comprises representatives originating exclusively from animal GITs (e.g., mammals, reptiles, and insects), suggesting an evolution specialized towards digestive tract microbiomes [23,24]. This clade has representatives grown in enrichment cultures, derived from animal and human GITs or kitchen wastes: *Ca.* ‘Methanomethylophilus alvus’, *Ca.* ‘Methanoplasma termitum’, and *Ca.* ‘Methanogranum caenicola’, as well as strains RumEn M2 and ISO4-H5 [20,21,22,23,26,27]. It has notably been studied in human GITs, appears to be increasing with age of the person, and was predominantly associated with elderly subjects being in good health condition [9,24,28,29,30]. 

The second cluster of *Methanomassiliicoccales*, referred in the literature to as the ‘free-living’ clade, corresponding roughly to *Methanomassiliicoccaceae*, is much less documented. It comprises mainly members detected by molecular methods in terrestrial and aquatic ecosystems, and known in their vast majority only through 16S rRNA or *mcrA* gene sequences. Sequences affiliated with this ‘free-living’ clade have been detected in various common and extreme habitats, such as seafloor sediments, peatland soils, terrestrial hot springs, or mangroves [23,31,32,33]. To date, in addition to fragmentary environmental DNA sequences detected through molecular PCR-based methods, our knowledge of this ‘free-living’ clade is limited to few metagenome-assembled genomes (MAGs) and three cultured strains sometimes predominantly active in these environments [15,16,23,34]. So far, the few cultured strains from this clade are paradoxically derived from human GIT, i.e., *Methanomassiliicoccus luminyensis* (*M. luminyensis*, the only strain of *Methanomassiliicoccales* isolated in pure culture) and *Ca.* ‘Methanomassiliicoccus intestinalis’ or from ruminant GITs, i.e., strain RumEn M1 [15,16,23]. However, no *Methanomassiliicoccales* belonging to the ‘free-living’ clade has been isolated or enriched from an aquatic or terrestrial environment. 

The goal of this study was to culture representatives of the ‘free-living’ clade of *Methanomassiliicoccales* from a natural environment, to obtain information on their biology and make assumptions about possible adaptations to terrestrial and aquatic ecosystems, and from a more general point of view, on a type of methanogenesis that seems to be widespread within the recently discovered methanogenic lineages. *Methanomassiliicoccales* abundance was determined for 22 samples from a variety of natural environments worldwide, with various physico-chemical characteristics, out of which, six samples were selected to perform enrichment in slurries. A metagenomic approach was applied and led to reconstruct a high-quality MAG of a *Methanomassiliicoccales* strain from the ‘free-living’ clade coming from a natural setting, providing matter for a phylogenetic and functional characterization.

## 2. Materials and Methods

### 2.1. Site Description

Eighty-six samples were collected from reported reduced areas of various ecosystems (marine sediments, deep-sea hydrothermal vents, marine pockmarks, submarine mud volcano, deep-sea hypersaline anoxic brine, peatland soils, lakes, hot springs, river sediments) during several sampling expeditions from 40 worldwide locations and stored in the laboratory for further investigations. Location and detailed information on sampling sites, depths, and sampling expeditions are given in Table S1. After a first molecular screening (as described in Text S1) performed to detect *Methanomassiliicoccales*-containing samples by PCR targeting 16S rRNA gene sequences of *Methanomassiliicoccales* and then confirmation of their presence by metabarcoding, 22 positive samples were selected for further investigations (Table 1).

The 22 samples used for an in-depth study were the following ones: (i) five samples were collected in a peatland area (Brittany, France), including four soil samples (MOUG1–MOUG4) and one sediment sample from a freshwater stream (MOUG5); (ii) four samples were collected from the active center of the deep-sea methane-emitting Håkon Mosby mud volcano, in the Barents Sea (Arctic Ocean) (HM1–HM4); (iii) three anoxic samples were collected in the water column of the meromictic crater Lake Pavin (Auvergne, France) at 60, 70, and 80 m water depth (PAV60-PAV70-PAV80); (iv) one geothermal hot spring sediment sample was collected from the Xiada reservoir of the Xiamen botanical garden (China) (XIA); (v) one geothermal spring sediment sample was taken from a hot spring at the Kerguelen islands (Kerguelen archipelago, France, Indian Ocean) (KERG); (vi) two samples were originating from the athalassic (MgCl_2_-rich) deep-sea hypersaline anoxic brine (DHAB) Kryos, in the Mediterranean Sea, respectively, collected from layers at 150 g L^−1^ salts (mainly MgCl_2_) (KRY150) and 238 g L^−1^ (mainly MgCl_2_) (KRY238); (vii) three sediments samples were collected in a pockmark field in the Mozambique Channel (off the Madagascar island) (MOZ1–MOZ3); (viii) one superficial marine sediment sample was collected from a marine bay influenced by freshwater inputs (DOUR) (English Channel, France); (ix) one deep-sea sediment sample was collected from the South West Indian Ocean (COMRA); and (x), finally, a freshwater sediment sample was taken from a stream (PENF) (Brittany, France). All the investigated samples were described and conditioned during each cruise and field expedition before being stored at 4 °C under anoxic conditions for culture and at −80 °C for DNA extraction. More detailed descriptions of these samples are given in Text S2 and Table S1.

### 2.2. DNA Extraction

Due to the various nature and composition of the environmental matrices, a standardized DNA extraction protocol, combining mechanical lysis by bead-beating and chemical lysis, was applied in triplicate to each sample onto 0.5 g of environmental matrix. One negative control was included and contained 0.5 mL of DNA-free sterile water. The mechanical lysis step was followed by a chemical lysis, based on the use of detergents (SDS and sarkozyl) and proteinase K, and was followed by a phenol/chloroform/isoamyl alcohol extraction of nucleic acids. The detailed procedure is described in Text S3. Elution of total DNA extracts was performed in 100 µL EB buffer (10 mM Tris-Cl, pH 8.5). Nucleic acid solution quality was determined using the NanoDrop™ 8000 (Thermo Scientific, Waltham, MA, USA) spectrophotometer. Double-strand DNA concentration was measured using the kit Quantifluor™ dsDNA system (Promega, Madison, WI, USA), following the manufacturer’s instructions.

### 2.3. Metabarcoding Sequencing and Sequence Analysis

The hypervariable partial SSU V4 region of the community 16S rRNA genes were analyzed to characterize the prokaryotic diversity in bulk samples and in substrate-amended slurries (DOUR, MOUG2, and PENF, respectively). Amplifications were carried out using the primer pair 515F-Y (5′-GTG YCA GCM GCC GCG GTA A-3′) [35] and 806R (5′-GCA CTA CNV GGG TWT CTA AT-3′) [36], with barcode on the forward primer, following the indexing and PCR conditions described elsewhere [37]; see https://www.earthmicrobiome.org/protocols-and-standards/16s/. The amplicons were then sequenced by the company Molecular Research-MrDNA (Shallowater, TX, USA), using the Illumina MiSeq (2 × 300 bp, paired-end reads; amplicon size: ~390 bp) technology, as described elsewhere [37]; see https://www.earthmicrobiome.org/protocols-and-standards/16s/. Raw reads were processed using the FROGS (Find, Rapidly, Operational taxonomic units (OTUs) with Galaxy Solution) pipeline [38] implemented on Galaxy (v. r3.0-1.4; Toulouse platform (see https://www.genotoul.fr/)) to generate FASTQ file, perform read quality controls, merge paired-end reads, remove unique singletons, and perform clustering by aggregation. Operational taxonomic units (OTUs) were generated from representative sequences (253 ± 20% bp) clustered as described elsewhere [38]. Singletons and potential chimeras were then removed to discriminate against sequencing artefacts. This resulted in 2,074,891 paired-end reads, forming, respectively, 14,096 and 11,843 OTUs, for bulk samples and cultures, respectively. Taxonomic affiliations of OTU representative sequences were done against the release 138 of the SILVA SSU database (www.arb-silva.de) and then double-checked against the RDP Classifier (v. 2.12). The taxonomic position of all *Methanomassiliicoccales-*related sequences was further analyzed by integrating the representative sequences into a reference dendrogram, using the distance-based phylogeny reconstruction algorithm BioNJ, as detailed in Text S4. The tree was visualized with iToL (v. 5.7; https://itol.embl.de/) (Figure 2).

The 16S rRNA gene amplicons dataset was analyzed using the phyloseq package (v. 1.28.0 R) [39] on program R studio (v. 1.3.5001). The OTUs abundance, assigned taxonomy, and sample contextual data were integrated into a phyloseq object before removing potential reagent contaminants using the decontam package (v. 1.4.0) [40]. Then, the metagenomSeq package (v. 1.26.3) [41] was used to normalize the dataset of the different samples by cumulative sum scaling (CSS), a median-quantile normalization which corrects differences in library sizes. Finally, all statistical analyses were performed with the phyloseq package. Extraction of 16S rRNA gene sequences representative of the OTUs and phylogenetic analyses are detailed in Text S4. A Bray-Curtis index was calculated to assess the dissimilarity between total microbial communities in environmental samples by observing ecological distances.

### 2.4. Quantitative Polymerase Chain Reaction

The abundance of 16S rRNA genes from *Methanomassiliicoccales* in natural samples and substrate-amended slurries was estimated using quantitative PCR (qPCR) with the primers AS05 Fw (5′-GGG GTA GGG GTA AAA TCC TG-3′) (this study)—AS2 Rv modified (5′-AAC AAC TTC TCT CCG GCA CT-3′) [28]. Quantifications were performed in triplicate with 5 ng of DNA template (5 μL of 1 ng μL^−1^ DNA template) (+ negative controls). Amplification reactions were carried out in a StepOnePlus™ Real-Time PCR system (Applied Biosystems, Foster City, CA, USA), in a final volume of 25 μL using the kit PerfeCTa^®^ SYBR^®^Green SuperMix (Quanta Biosciences, Gaithersburg, MD, USA, supplier: VWR), 200 nM primers targeting *Methanomassiliicoccales* partial 16S rRNA gene sequences, and 5 μL of DNA template. For 16S rRNA gene copies quantification, qPCR conditions were as follows: 1 initial denaturation step at 95 °C for 10 min, followed by 40 cycles of denaturation at 95 °C for 15 s, then annealing and extension at 58 °C for 60 s. qPCR specificity was checked based on melting curve analysis, PCR efficiency (90–110%), r² of standard curves (>0.99), Ct (≤30), electrophoretic migration of amplicons, and sequencing checking of the PCR products. Standard curves were obtained from 10^−1^ to 10^8^ copies µL^−1^ of plasmids containing a full 16S rRNA gene insert of *Methanomassiliicoccus luminyensis* (DSM 25720) (cloned in the vector pCR™2.1-TOPO™ of the TOPO™TA cloning^®^ kit (Invitrogen)). The r^2^ of standard curves obtained by qPCR were equal to or more than 0.992 and PCR efficiencies between 97 and 105%. The qPCR results were expressed in terms of copy gene numbers per gram of wet weight sample or per ml. For the environmental samples, they were considered as exploitable and biologically relevant when reaching at least 1 × 10^3^ gene copies per ml or gram of wet weight of sample.

### 2.5. Culture-Based Incubation Experiments

#### 2.5.1. Medium Composition for Culture-Based Experiments with Environmental Slurries

Enrichment slurries targeting hydrogenotrophic methyl-dependent methanogens were performed with environmental samples either (i) having the highest *Methanomassiliicoccales* 16S rRNA copy number per gram of wet weight or ml quantified (ii) or corresponding to ecosystems with extreme and unusual physical-chemical parameters (KRY150—MgCl_2_-rich brine subjected to more than 33 MPa pressure; KERG—geothermal hot spring). The medium composition was designed based upon (i) previously published recipes used by culture collections (DSMZ medium 141) (Text S5) to grow or enriched *Methanomassiliicoccales*, (ii) on published enrichment media [15,17,42,43], and (iii) on metabolic information derived from metagenome-assembled genomes (MAGs) of *Methanomassiliicoccales* available in public databases [27]. Most of these recipes were based on substrate-rich composition, notably comprising a high concentration of methanol and/or methyl-compounds to promote *Methanomassiliicoccales* growth.

Basal medium contained, for 1 L of distilled water: 1 g NH_4_Cl, 1 g Na-acetate, 0.1 g peptone from casein (Merck, Germany), 0.5 g KH_2_PO_4_, 20 mL fatty-acid solution, 5 mL porcine hemin solution, 1 mL tungstate-selenite solution, 1 mL trace elements solution SL10 [44], 5 mL K3 vitamin solution, NaHCO_3_ (10% m/v), and resazurin (0.0001%) (see Text S5 for more details concerning the preparation of these solutions).

For culture-based incubation experiments performed with anoxic freshwater (PAV60), peat (MOUG2), geothermal hot spring water (KERG), and river sediments (PENF), 5 g NaCl, 0.4 g MgSO_4_·7H_2_O, and 0.05 g CaCl_2_·2H_2_O were initially added to the basal medium. Since the MOUG2 sample had relatively high acetate concentrations (>4 mM), no Na-acetate was added to the medium. For culture-based incubation experiments performed with marine samples (DOUR, KRY150), 20 g NaCl, 3.45 g MgSO_4_·7H_2_O, and 0.14 g CaCl_2_·2H_2_O were added to the basal medium. As Kryos DHAB is concentrated in magnesium [45], the magnesium concentration was adjusted to 150 g L^−1^ with MgCl_2_ for the slurry KRY150. The pH of the media were adjusted to 5.5 or 7.5 before boiling. After boiling, 0.5 g L^−1^ of cysteine HCl.H_2_O was added to the degassed medium under a N_2_/CO_2_ (80:20, *v*/*v*; 0.3 bar) flow, and the medium was then dispensed into 500 mL Schott flasks (135 mL of medium per flask). The flasks were closed with butyl rubber stoppers, and filled with a N_2_/CO_2_ (80:20, *v*/*v*; 0.3 bar) atmosphere before autoclaving. Vitamins, bicarbonates, trace elements, and reducing agent Na_2_S.9H_2_O (10% *w*/*v*) were then added to the medium before addition of substrates and cofactors from anoxic sterile stocks solutions: 40 mM trimethylamine (TMA), 40 mM methanol, 10 µM coenzyme M, and a gas phase of H_2_/CO_2_ (80:20, *v*/*v*; 1 bar). Since all published *Methanomassiliicoccales* MAGs seem to not contain the gene set required for coenzyme M production, 10 µM of coenzyme M were added to the media to promote their growth [27]. Media were then inoculated with 10% (*w*/*v* or *v*/*v*) original samples and incubated in the dark, without shaking, at 30 °C (DOUR, PAV60, PENF, MOUG2), 37 °C (KRY150), and 60 °C (KERG), respectively. When depleted, methanogenesis substrates were renewed by the addition of 40 mM TMA, 40 mM MeOH, and a renewal of the gas phase with H_2_/CO_2_ (80:20, *v*/*v*; 1 bar) to maintain methanogenesis activity. Uninoculated media were used as negative controls.

#### 2.5.2. Monitoring of Culture-Based Incubation Experiments

The evolution of microbial diversity within substrate-amended slurries has been monitored by carrying out metabarcoding (as described in Section 2.3) and by quantifying *Methanomassiliicoccales* (as described in Section 2.4), on a weekly basis, on three technical replicates. Although the metabarcoding and qPCR analyses were made from the same DNA extracts, amplifications were performed with different polymerases and different primers (see Section 2.3 and Section 2.4). At the same time as these molecular analyses, an analytical monitoring of metabolic substrates and products, and, in particular, of those of hydrogenotrophic methyl-dependent methanogenesis, was carried out as described below.

#### 2.5.3. Quantifications of Substrates and Metabolic Products

Headspace gas CH_4_, H_2_, N_2_, and CO_2_ were measured using a modified INFICON/Micro GC FUSION Gas Analyzer (INFICON, Basel, Switzerland) fitted with a pressure gauge and two conductivity detectors, and using argon as a carrier gas, as detailed in Text S6.

Samples for cation analyses were analyzed using a Dionex ICS-900 Ion Chromatography System (Dionex, Camberley, UK) coupled with a CERS 500 4 mm suppressor and a DS5 conductivity detector (40 °C) and fitted with an RFC-10 Reagent-Free Controller™, an ASDV autosampler, and an IonPac CS16 column maintained at 60 °C in an UltiMate™ 3000 Thermostated Column Compartment (Thermo Scientific, Waltham, MA, USA). Anions concentrations were quantified by anion chromatography as described elsewhere [46]. The detailed protocol is given in Text S6. Analysis of methanol concentrations was carried out using an Agilent 6890N Gas Chromatograph (GC) (Agilent Technologies, Santa Clara, CA, USA) coupled with a Flame Ionization Detector (FID) instrument as described in Text S6.

### 2.6. MAG Sequencing and Annotation

Metagenomic sequencing was carried out with the DNA extracted after 8 weeks of incubation of the slurry PENF (corresponding to the T8 sample of the culture-based experiment). This sample was in fact the one with the highest qPCR estimation in *Methanomassiliicoccales*. The aim was to reconstruct a MAG of *Methanomassiliicoccales* in order to learn more about the genetic, physiological and adaptive potential of the ‘free-living’ *Methanomassiliicoccales* cluster. A comparison of this MAG with genomes of ‘host-associated’ representatives was also intended to identify potential adaptations to the environment. Short read DNA sequencing was performed by Fasteris SA (Plan-les Ouates, Switzerland), using the NovaSeq 6000 technology (2 × 100 bp, 100,000,000 paired-end reads; Protocol genomic Nano, 250 bp insert size section). Libraries constructions and quality controls were performed by the sequencing facility and verified with FastQC (v. 0.11.8—https://www.bioinformatics.babraham.ac.uk/projects/fastqc/).

MAGs were reconstructed using the Let-it-bin pipeline (https://github.com/QuentinLetourneur/Let-it-bin). Reads were first trimmed with AlienTrimmer [47]. Redundant reads were then removed using khmer [48]. Filtered reads were assembled with Megahit [49] and metaSpades [50]. Binning was performed with MaxBin2 [51], MetaBAT [52], and MetaBAT2 [52]. MAGs assemblies’ statistics were obtained with Quast (v. 5.0.2; https://github.com/ablab/quast). MAG completeness and potential contamination were controlled with CheckM (v. 1.1.2—https://ecogenomics.github.io/CheckM/) [53]. *Methanomassiliicoccales* MAGs were identified by phylogenetic approaches described below. Two *Methanomassiliicoccales* MAGs obtained from metaSpades and Megahit assemblies contained contigs with identical sequences but interrupted at different loci. In order to improve the contig length, the contigs from these two MAGs were assembled with cap3 [54], leading to MXMAG1 MAG. A second MAG, MXMAG2, was obtained with metaSpades. Both were analyzed and annotated with the fast annotation software Prokka (v. 1.14.6—https://github.com/tseemann/prokka), Dfast (v. 1.2.5—https://github.com/nigyta/dfast_core), the online version of the RAST software (v. 2.0—http://rast.theseed.org/FIG/rast.cgi), and the MicroScope Microbial Genome Annotation and Analysis Platform (MaGe) (https://mage.genoscope.cns.fr/microscope/home/index.php), using KEGG and BioCyc databases with default parameters and databases for all of the five software/pipelines [55,56,57,58]. Functional annotations of predicted CDSs were further compared with NCBI (v. 2.10.0+), and UniProtKB database (release 2020_07).

### 2.7. Phylogenetic Position of the MAGs and Comparative Genomics

The archaeal MAGs obtained from the T8 sample metagenome were first placed in the archaeal phylogeny, including all major lineages of archaea to identify *Methanomassiliicoccales* representatives. The two *Methanomassiliicoccales* MAGs were then placed in a tree, including 17 other *Methanomassiliicoccales* genomes and 14 of the closest non-*Methanomassiliicoccales* genomes. Both phylogenies were based a concatenation of 40 conserved phylogenetic markers [8] were constructed in IQ-TREE with Maximum Likelihood (LG + F + G4) [59]. Average Nucleotide Identity (ANI) scores were calculated using the ANI calculator tool provided by the EzBioCloud web server (https://www.ezbiocloud.net/tools/ani) [60] and using the JSpeciesWS (http://jspecies.ribohost.com/jspeciesws/) [61], between the high-quality MAG and its closest relative organisms *Methanomassiliicoccus luminyensis* B10^T^ (Accession number NZ_CAJE00000000.1) and *Candidatus* ‘Methanomassiliicoccus intestinalis’ Issoire-Mx1 (Accession number: NC_021353.1). DNA-DNA hybridization (DDH) estimate values were calculated using the genome-to-genome distance calculator (GGDC, v. 2.1, formula 2) [62] between the high-quality MAG and two closest relative organisms cited above.

Representative *Methanomassiliicoccales* genomes from the ‘host-associated’ and ‘free-living’ clades were compared to reconstructed MAGs using the MaGE platform’s Pan-genome Analysis tool (https://mage.genoscope.cns.fr/microscope/home/index.php), based on the clustering algorithm SiLiX (http://lbbe.univ-lyon1.fr/-SiLiX-.html) which clustered genomic CDSs by 50% or/and 80% amino-acid identity and 80% amino-acid alignment coverage, with permissive parameters. Resulting CDSs were blasted against the UniprotKB database, and hypothetical protein CDSs were analyzed with the InterProScan webserver (https://www.ebi.ac.uk/interpro/) for functional predictions.

### 2.8. Co-Occurrence Network Analysis

Network analysis was used to describe co-occurrences/putative associations between taxa within the environmental samples and in the substrate-amended slurries, respectively. On the basis of the co-occurrences of taxa found in the different samples and cultures, a matrix of correlation was computed between all the previously assigned OTUs, defined as mentioned previously (non-parametric Spearman). In this network, each node represented an OTU, and the links between nodes represented the positive correlation between them. The strategy used to analyze its topology was the same as described elsewhere [63]. In order to catch the most relevant information embedded in these co-occurrences, we started to analyze the networks at their respective percolation thresholds, which are the values at which the overall organization of the networks is a minimal giant cluster bearing the minimum number of links required for an overall connectivity of the system [64]. The visualization and statistical analysis of the network at these thresholds was done with Gephi (v. 0.9.2) [65]. Notably, the hierarchical structure was explored through a modularity algorithm [66]. An in-depth bibliographical analysis of the metabolic characteristics of known representatives of the taxa co-occurring in this network was carried out to focus on potential physiological characteristics described for the cultured taxa of these groups and determine if a putative physiology could be inferred for a given group (Table S5). The connectivity of *Methanomassiliicoccales* in each of the modules was characterized according to the metabolic characteristics of each taxon belonging to the same module.

### 2.9. Availability of Data and Materials

The MAGs’ bins generated and analyzed in this study are available under the Whole Genome Shotgun project deposited at DDBJ/ENA/GenBank under the accession numbers JAEILR000000000 v.1 (MXMAG1) and JAEILS000000000 v.1 (MXMAG2). The 16S rRNA gene sequences k119_35148 and k119_91804 are, respectively, available under the accession numbers MW383280 and MW383279. The 16S rRNA gene sequence generated by metabarcoding are available under the accession numbers MW386093-MW386158 in DDBJ/ENA/GenBank databases.

## 3. Results and Discussion

### 3.1. Diversity and Abundance of Methanomassiliicoccales in a Wide Range of Habitats

#### 3.1.1. Molecular Pre-Screening of Various Environmental Samples

**Figure 1:**
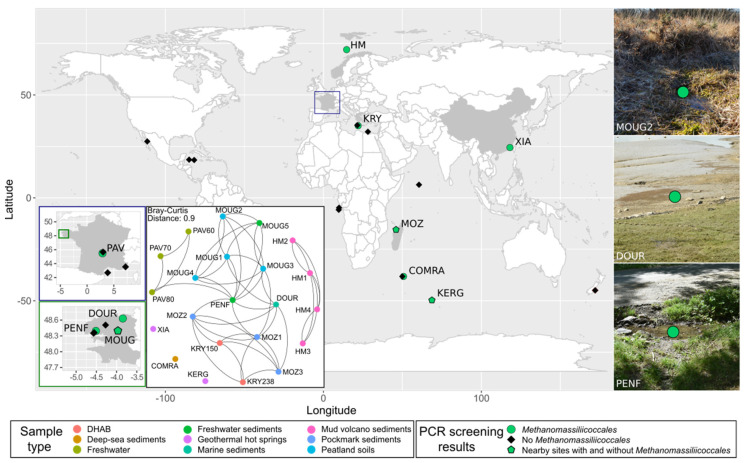
Distribution of environmental samples screened for *Methanomassiliicoccales*. On the map, green dots indicate the presence of Operational taxonomic units (OTUs) assigned to *Methanomassiliicoccales* in the sample; black diamonds indicate samples in which no *Methanomassiliicoccales* was detected by PCR with primers targeting *Methanomassiliicoccales*. Green and black pentagons show the position of nearby sites characterized by the presence of *Methanomassiliicoccales* for some, and their absence for others. The Network based on Bray-Curtis index shows the dissimilarity in the composition of total microbial communities between samples; pictures on the right represent, respectively, the MOUG2, DOUD, and PENF sampling sites.

In order to explore the distribution of *Methanomassiliicoccales* associated with terrestrial and aquatic ecosystems a first molecular screening was carried out on 86 anoxic samples collected worldwide. These samples comprised a wide diversity of habitats (marine sediments, deep-sea hydrothermal vents, marine pockmarks, submarine mud volcano, deep-sea hypersaline anoxic brine, peatland soils, lakes, hot springs, river sediments) displaying a large range of physico-chemical conditions (pH, temperature, salinity, pressure). *Methanomassiliicoccales* were detected using PCR-based metabarcode screening (with the GoTaq^®^ G2 DNA polymerase) in 26% (*n* = 22) of all samples, including coastal and deep-sea marine sediments, submarine mud volcano samples, anoxic lake waters, freshwater sediments, deep-sea brine samples, hot spring sediments, and peatland soils (Figure 1). A Bray-Curtis index calculated between these 22 samples revealed that the microbial community compositions were mainly structured according to sample type and by sampling site (see network topology in Figure 1; Table 1). So far, some of the anoxic habitats where *Methanomassiliicoccales* have been detected were reported to contain fermentation products and methylated compounds [67,68,69,70,71]. The methylated compounds result from the anaerobic degradation of organic compounds, such as pectin (for methanol) or *N*-methylated compounds (e.g., choline, betaine or trimethylamine-*N*-oxide), which are particularly represented in marine habitats [72], where they mainly serve as osmoregulators in organisms. A large part of this work has focused on marine habitats where the terminal degradation of organic matter results from microbial sulfate-reduction or methanogenesis reactions [68].

This work highlighted the presence of *Methanomassiliicoccales* in new geographical areas (Kerguelen archipelago in the Indian Ocean and the Brittany region in France, respectively), in environments where their presence had not yet been reported (submarine mud volcanos) and in habitats sometimes characterized by extreme conditions, such as high pressures, high salinities, high temperatures, or low pH (Table 1). As previously reported, the presence of *Methanomassiliicoccales* was detected in various ecosystems with various physical-chemical parameters, such as (i) in terrestrial hot springs where temperatures were between 50 and 82 °C (KERG, XIA) [31,73,74], (ii) in a large number of samples where temperatures were more moderate (between 8 and 30 °C), as in peatland soils, where the pH was slightly acidic (MOUG2, MOUG4) [23,75], (iii) in submarine mud volcanoes (HM1-4) and subseafloor sediments (COMRA) located at significant depths (between 1200 and 3338 m) [32,76], (iv) in non-saline (PAV60, PAV70, PAV80) [22,77] (v) to hypersaline (KRY150, KRY238) [45] environments, and (vi), finally, in a habitat subject to anthropogenic pollution and freshwater inputs (DOUR) [78]. These results reinforce the hypothesis that *Methanomassiliicoccales* might have a physiological diversity and potentially encompass mesophilic, thermophilic, halophilic, neutrophilic, acidophilic, and maybe even piezophilic taxa, as long as the amplified sequences were derived from active cells, and not from free DNA or dormant cells.

Metabarcode sequencing targeting variable regions of 16S rRNAs gene sequences from *Bacteria* and *Archaea* and qPCR targeting *Methanomassiliicoccales* were then carried out to confirm these results and determine the diversity and abundance of *Methanomassiliicoccales* in these samples.

#### 3.1.2. Diversity and Abundance of *Methanomassiliicoccales*

**Figure 2:**
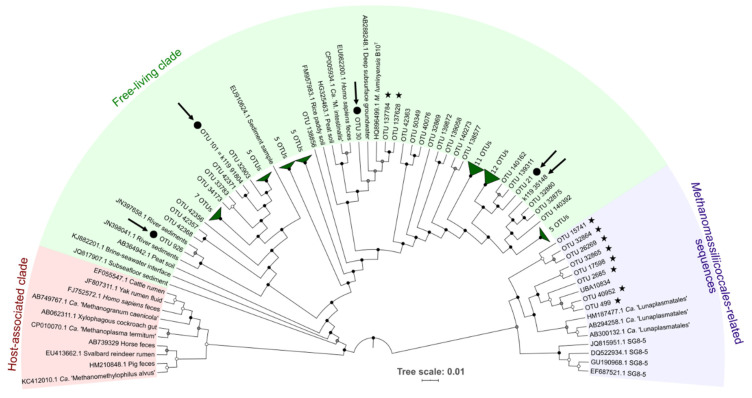
Dendrogram showing the relationships between 16S rRNA gene sequences (253 bp) detected in environmental samples, in substrate-amended slurry (PENF slurry) and inferred to metagenome-assembled genomes (MAGs), among *Methanomassiliicoccales* [22]. The 85 OTUs of *Methanomassiliicoccales* detected in this study are shown on this dendrogram. OTUs detected only in environmental samples are indicated by a black star. OTUs without a symbol were detected in the culture-based experiment performed with the sediment PENF. OTUs indicated by a circle were detected both in bulk samples and in the culture-based experiment. The 16S rRNA gene sequences k119_35148 and k119_91804 were extracted from the metagenome sequences of the culture-based experiment PENF, after 8 weeks of incubation. Sequences discussed in the text are indicated by black arrows. The *Methanomassiliicoccales*-related sequences encompassed representative 16S rRNA gene sequences of *Ca.* ‘Lunaplasmatales’, UBA10834 and SG8-5 [80]. This reconstruction was performed on partial 16S rRNA gene sequences (253 bp), using the BIONJ method [81], with the modifications of Jukes and Cantor and using 1000 bootstrap replicates. Black-filled dots indicate nodes with bootstrap supports <50%, grey-filled dots 50–75%, and white-filled dots show support values between 75 and 100%. The top-right insert indicates the relative abundance of the main *Methanomassiliicoccales*-affiliated OTUs in the substrate-amended slurry PENF, after 8 weeks of incubation.

Metabarcoding analyses confirmed the presence of *Methanomassiliicoccales* in the 22 samples where the presence of *Methanomassiliicoccales* was initially detected. The diversity of *Methanomassiliicoccales* in these samples was low. Indeed, out of the 14,809 prokaryotic OTUs identified by processing the dataset, only 6 OTUs were confirmed to belong to *Methanomassiliicoccales* based on the automatic assignment made by SILVA 138 database and by finer phylogenetic analyses performed with a set of *Methanomassiliicoccales* sequences and closely related lineages (Tables S2 and S3). These 6 OTUs (OTUs 21, 30, 101, 926, 137,628, and 137,784) fell into the ‘free-living’ cluster of the *Methanomassiliicoccales* (Figure 2), in the *Methanomassiliicoccaceae* family. Among them, OTU30 was tightly related to *M. luminyensis* B10^T^ 16S rRNA sequence (99.6% sequence similarity) and was present in all of the 22 selected samples, supporting the hypothesis that *M*. *luminyensis* might be inferred to a wide range of environmental conditions (Table 1) [24,79]. Eight other OTUs of the dataset were found to belong to very closely related lineages of *Methanomassiliicoccales* referenced as *Ca.* ‘Lunaplasmatales’ and UBA10834 [80]. 

The total abundance of *Methanomassiliicoccales* within samples was determined by quantitative PCR (Table S4). For samples where quantification has been possible, abundance of *Methanomassiliicoccales* was generally very low, ranging from 1.62 × 10^3^ 16S rRNA gene copies per gram of wet matrix in MOZ1 samples, to 7.5 × 10^4^ 16S rRNA gene copies per gram of wet weight soil in the peatland soil sample MOUG2. Many samples did not contain *Methanomassiliicoccales* abundances falling within the range set in this study as being biologically relevant for crude environmental samples (≥1 × 10^3^ gene copies per ml or gram of wet weight of sample). These results were congruent with the literature, which indicate that these methanogenic archaea are very scarce in the environment [14,23]. However five samples contained non-negligible effectives of *Methanomassiliicoccales*: (i) soil samples from the Mougau peatland (MOUG1 and MOUG2) with respective abundances of 7.5 × 10^4^ and 6.2 × 10^4^ 16S rRNA gene copies of *Methanomassiliicoccales* per gram of wet weight (and 34.4% and 1.7% of total known methanogen’s sequences in the metabarcoding dataset, respectively); (ii) sediments from the stream tributary to the Penfeld river (PENF; 5.3 × 10^4^ 16S rRNA gene copies per gram of wet weight, 69.8% of total methanogen’s sequences); (iii) a sample from the Pavin lake collected at 60 m depth (PAV60; 1.3 × 10^4^ 16S rRNA gene copies per ml; 60.0% of total methanogen’s sequences), and iv) a sediment sample from the Dourduff-en-Mer marine Bay (DOUR, 6.5 × 10^3^ 16S rRNA gene copies per gram of wet weight, 38.6% of total methanogen’s sequences) and a hot spring water sample from the Kerguelen Island (KERG, 6.4 × 10^3^ 16S rRNA gene copies per gram of wet weight, 79.5% of total methanogen’s sequences).

### 3.2. Enrichment Cultures Targeting Methanomassiliicoccales

Six samples were selected for enrichment cultures of *Methanomassiliicoccales*. Sample selection was based on: (i) qPCR abundance values of *Methanomassiliicoccales* exceeding 1 × 10^3^ 16S rRNA gene copy numbers per ml or gram of wet weight sample (DOUR, MOUG2, PAV60, PENF), and/or (ii) corresponding to ecosystems different from those in the gastrointestinal tract from which the *Methanomassiliicoccales* strains were isolated to date (KRY150—MgCl_2_-rich brine subjected to more than 33 MPa pressure; KERG—hot spring). To reach this objective, a medium was developed in this work to promote growth of *Methanomassiliicoccales*. This medium contained methyl compounds (MeOH and TMA, 40 mM as described by Reference [15,43]) and dihydrogen in order to promote methyl-dependent hydrogenotrophic methanogenesis. 

**Figure 3:**
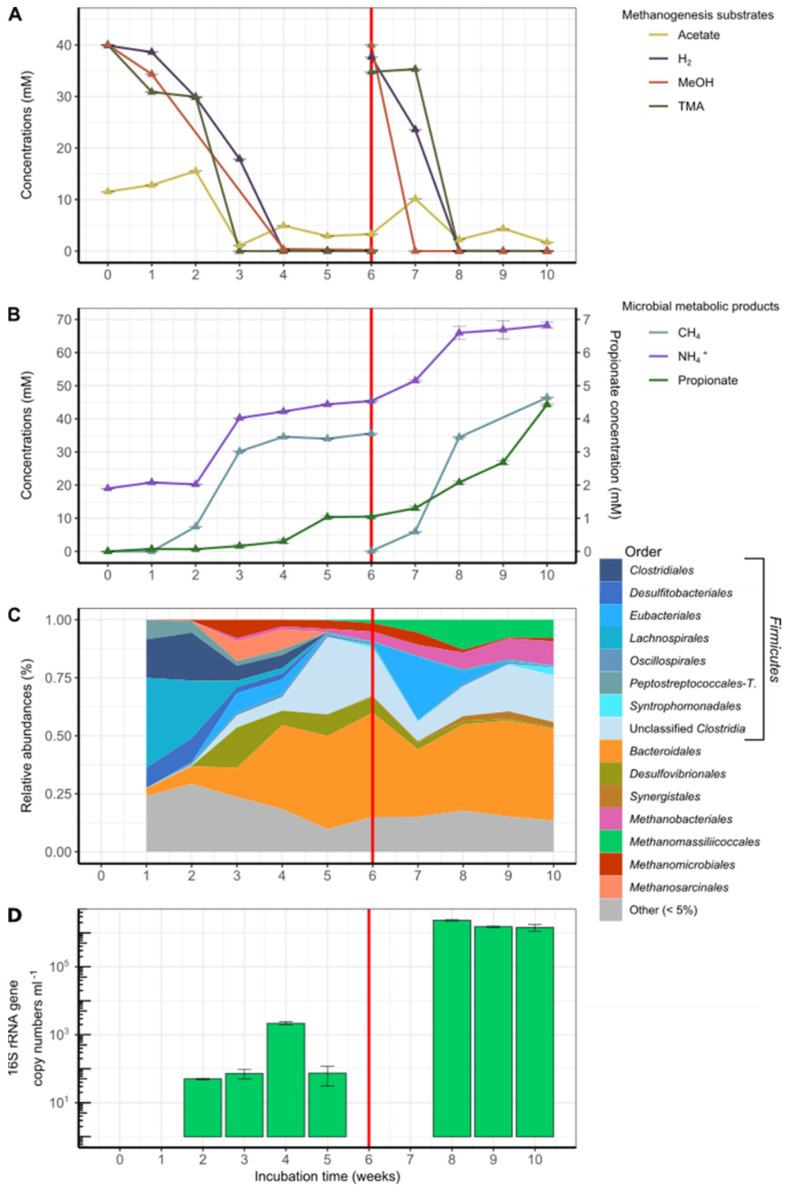
(**A**) Consumption kinetics of methanogenesis substrates during the weeks of incubation of the substrate-amended slurry performed with the PENF sample; (**B**) Consumption kinetics of some metabolic products (methane, ammonia, propionate) during the weeks of incubation of the substrate-amended slurry performed with the PENF sample. Concentrations of propionate are indicated on the right vertical axis of the figure and other microbial products (CH_4_ and NH_4_^+^) are indicate on the left vertical axis. The vertical red line indicates a novel addition of trimethylamine (TMA), methanol and H_2_/CO_2_ at T6 to stimulate methanogenesis; (**C**) Trends in microbial diversity revealed by metabarcoding; (**D**) *Methanomassiliicoccales* abundances determined by qPCR (logarithmic scale). *Methanomassiliicoccales* could not be quantified by qPCR in 4 samples (T0, T1, T6, and T7). Average copy numbers are given on the chart bars. Renewal of methanogenesis substrates (H_2_, CO_2_, MeOH, TMA) after six weeks of incubation is represented by the vertical red lines. *Peptostreptococcales*-*T*.: *Peptostreptococcales-Tissierellales*.

Of the 6 substrate-amended slurries incubated and monitored using geochemical and molecular approaches for 10 weeks, 3 did not produce methane de novo (PAV, KERG, KRY150). Two others led to methane and ammonium production but without *Methanomassiliicoccales* enrichment (DOUR and MOUG2) (Figure S1). No methane production or cell growth was detected in the uninoculated control culture. The only culture that showed production of methane associated with an increase in the copy number of *Methanomassiliicoccales* 16S rRNA sequences over time was the PENF freshwater sediment sample (Figure 3).

After 6 weeks of incubation, the added substrates were consumed. Hence, slurries were reamended with MeOH, TMA, and H_2_ in order to overcome potential growth limitation of *Methanomassiliicoccales*. A constant increase in methane concentrations followed the consumption of these substrates in the first phase of the culture, as well as after the renewal of the substrates and the gas phase, suggesting that methanogenesis was occurring in this culture (Figure 3A,B). It is noteworthy that small amounts of dimethylamine (DMA) and monomethylamine (MMA) were detected one week after the beginning of the incubation (T1) (0.31 and 0.08 mM, respectively) and also one week after the new addition of TMA (T7) (0.16 and 0.10 mM, respectively). These products are intermediates in the degradation of TMA and probably reflect the progressive degradation of this substrate [72]. Additionally, a small amount (0.01 mM) of choline, a quaternary amine known to be degradable to TMA, ethanol, and acetate, by some fermentative microorganisms [68], was also detected at T6. A steady increase in propionate concentration over time also suggests that several fermentative microorganisms were active in this culture. In addition, variations in acetate concentrations were also observed throughout the incubation period, increasing from 11.53 to 15.53 mM between T0 and T2 before stabilizing between 1.08 and 4.94 mM between T3 and T10, interspersed with a sharp increase at T7 (10.14 mM), one week after the new addition of substrates. These variations reflect high microbial activity throughout the incubation period, since this compound can be a product and also a substrate of microbial metabolism (e.g., acetogenesis). Considering that the production of methane (respectively, 35.61 and 46.42 mol L^−1^ at T6 and T10) was higher than that of the initial available dihydrogen (respectively, 31.74 and 37.66 mol L^−1^ at T0 and T6), the stoichiometry of the methyl-dependent hydrogenotrophic methanogenesis was not conserved, confirming that several metabolisms were at work in this culture. In accordance, the metabarcoding analysis performed over time on this culture showed a large fraction of 16S rRNA gene sequences of methanogenic lineages (*Methanobacteriales*, *Methanomassiliicoccales*, *Methanomicrobiales*, and *Methanosarcinales*) and of taxa mainly reported in animal GITs [82,83] (*Firmicutes* (*Clostridiales*, *Desulfitobacteriales*, *Eubacteriales*, *Lachnospirales*, *Peptostreptococcales-Tissierellales*, and unclassified *Clostridia*), *Bacteroidales*, *Synergistales*, and taxa in lower relative abundance (<5%)), indicating that it was a complex microbial community, even after 10 weeks of incubation. Among the *Methanomassiliicoccales*-affiliated OTUs, sequences belonging to the OTUs 21, 30, 101, and 926 were detected throughout the incubation period and were predominant in the dataset. 

In terms of overall diversity, the species richness slightly decreased over incubation time (from T1 to T10: Shannon index, 3.11 to 2.61; Simpson index, 0.88 to 0.82) (Figure S2). It is noteworthy that the majority of lineages in which relative abundance increases over time (*Bacteroidales*, *Clostridiales*, *Desulfovibrionales*, and *Synergistales*), as well as those of *Methanomassiliicoccales*, were similar to cohorts associated with *Methanomassiliicoccales* previously described that had been inoculated under mesophilic conditions with bovine rumen fluid, kitchen wastes, or mixtures of *Gracilaria* sp. algae and marine sediments [20,23,84]. In this culture, *Desulfovibrionales* were dominated by sequences affiliated to the genus *Desulfovibrio*, a genus within which certain species are known to produce TMA, ethanol, and acetate from choline fermentation [85]. A predominance of *Bacteroidetes*, *Firmicutes*, and *Methanomassiliicoccales* was also reported for an environmental sample, in this case, a birch forest soil in winter [86]. Apart from methanogenic *Archaea*, other *Archaea* represented a marginal fraction of the total prokaryotic diversity, accounting for 46.2% and 2.3% of total read sequences at T1 and T2, respectively, to less than 0.1% of total sequences after the second week of incubation (Figure S3A,B).

A decrease in the number of sequences affiliated with the genera *Methanosarcina* (mainly composed of representative strains using H_2_/CO_2_, or R-CH_3_ or acetate) and *Methanomicrobiales* (using H_2_/CO_2_/formate) was observed over time, while the number and relative fraction of sequences affiliated with the genus *Methanobacterium* (*Methanobacteriales*; mainly growing on H_2_/CO_2_) and the order *Methanomassiliicoccales* (using H_2_ + R-CH_3_) increased (Figure 3C). Between T1 and T5, the proportion of *Methanomassiliicoccales* sequences within total methanogenic archaea decreased from 29.2% to 4.6%. However, their relative abundance among methanogens increased after 6th week of incubation (16.4%) to reach a maximum value at T8 (63.1%) (Figure S3C). From the 8th week of incubation (T8), the relative fraction of *Methanomassiliicoccales* decreased slightly and that of *Methanobacterium* spp. (*Methanobacteriales*) increased slightly, to both stabilize at about 50%. Interestingly, some *Methanobacterium* representatives are able to use ammonium as the only source of nitrogen [87]. At 8 weeks incubation time (T8), 85 *Methanomassiliicoccales* OTUs were detected in the incubated slurry and were all affiliated to the ‘free-living’ environmental clade, forming various clusters of sequences within this clade (Figure 2), and representing, 63.1% of methanogens-affiliated sequences, 63.1% of all archaeal sequences (Figure S3D) and 14.9% of total sequences (Figure S3E). Interestingly, 22 OTUs, including OTU101 and OTU926, formed a cluster not related to the genus *Methanomassiliicoccus*. With the exception of 4 OTUs (OTUs 21, 30, 101, and 926, respectively), the majority of these sequences detected in the culture-based incubation experiment have not been detected by metabarcoding on the environmental sample, suggesting that these taxa were extremely scarce in situ.

The total abundance of *Methanomassiliicoccales* increased over the incubation period, from 5.00 × 10^1^ at T2, to 2.35 × 10^6^ 16S rRNA gene copies of *Methanomassiliicoccales* mL^−1^ at 8 weeks of incubation (T8), before decreasing slightly to 1.43 × 10^6^ copies at T10 (Figure 3D). Unfortunately, no qPCR quantification of *Methanomassiliicoccales* could be obtained at T1, T6, and T7. Nevertheless, this nearly 5-log increase in the abundance of *Methanomassiliicoccales* demonstrated that there was enrichment (*p*-value < 0.0001, in ANOVA and Student’s t-test) and, therefore, growth of the *Methanomassiliicoccales* during incubation, on the medium developed in this study with the freshwater sediment sample PENF. This result is reinforced by the fact the number of *Methanomassiliicoccales* sequences also increased gradually and concomitantly over the incubation period, until time T8. 

The highest levels of *Methanomassiliicoccales* abundance results were obtained after 8 weeks of incubation, both in terms of copy number of the 16S rRNA gene sequences quantified by qPCR and among the total methanogenic community detected by metabarcoding (63.1% of the total methanogenic sequences). At that time, OTU21 was the predominant OTU of *Methanomassiliicoccales*, representing 86.5% of the total sequences of *Methanomassiliicoccales*, followed by OTU101 (13.4%) and then by the sequences of the other *Methanomassiliicoccales* OTUs (<1% in total) (Table S2). This result guided us to retain the T8 point for a metagenomic analysis.

### 3.3. Methanomassiliicoccales Genomic Features

Two *Methanomassiliicoccales*-affiliated MAGs were reconstructed from the T8 point of the substrate-amended slurry made from the PENF sample: one of high-quality (MXMAG1) and the other of low-quality (MXMAG2). 

#### 3.3.1. Taxonomic Position and General Features of the MAGs

**Figure 4:**
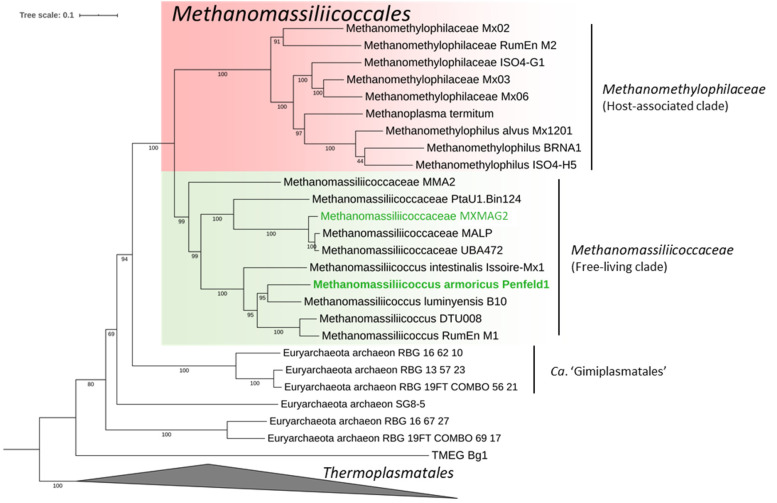
Maximum Likelihood phylogenomic tree (LG + F + G4) showing the position of the two *Methanomassiliicoccales* MAGs obtained in this study (in green) with respect to closely related MAGs and taxa. This phylogeny is based on a concatenation of 40 conserved phylogenetic markers (10,111 positions). The scale bar represents the average number of substitutions per site, and numbers indicate bootstrap replicates.

Following genome-wide taxonomic analysis (with IQ-TREE) based on concatenated protein reference trees, both MAGs were robustly placed in the order *Methanomassiliicoccales* (Figure 4). ANI scores for the MXMAG1 MAG calculated against *M. luminyensis* B10^T^ and *Ca.* ‘Methanomassiliicoccus intestinalis’ Issoire-Mx1 had OrthoANIu values of 74.73% and 66.54%, respectively. These values are much lower than the threshold criterion for prokaryotic species delineation proposed to be 95–96% [88], suggesting that this MAG corresponds to a different *Methanomassiliicoccales* representative than those previously described. 

The digital DNA-DNA hybridization (DDH) estimate values between MXMAG1 MAG and *M. luminyensis* B10^T^ was 19.60%, and the one with *Ca.* ‘Methanomassiliicoccus intestinalis’ Issoire-Mx1 was 21.40%, both of which were values well below the standard criterion (70%) for the delineation of a prokaryotic species [89], confirming that the MXMAG1 MAG belongs to a different genomic species than *M. luminyensis* and *Ca.* ‘Methanomassiliicoccus intestinalis’. Thus, we propose that MXMAG1 represents the type material of a novel *Methanomassiliicoccus* species, for which we propose the name *Candidatus* ‘Methanomassiliicoccus armoricus MXMAG1’ (ar.mo’ri.cus L. pl. fem. n. Armoricae, part of Gaul between the Seine and the Loire, including Brittany, where the sample used for the slurry were taken from; N.L. masc. adj. armoricus MXMAG1, pertaining to Brittany).

Apart from the 16S and 23S rRNA genes which were absent, the sequence of the MAG called *Ca.* ‘Methanomassiliicoccus armoricus MXMAG1’ met all of the criteria described elsewhere [90] to be considered a high-quality draft. This MAG consisted of 37 contigs with an overall size of 2,230,712 bp and a G+C content of 62.13 mol%. This size was intermediate between the genome of closest species, *M. luminyensis* and *Ca.* ‘M. intestinalis’, that are 2.6 Mbp and 1.9 Mbp, respectively [79]. CheckM estimated the genome to be 94.4% complete based on the presence of single-copy marker genes (9 markers were missing) and without contamination. Annotation with MaGe of *Ca.* ‘Methanomassiliicoccus armoricus MXMAG1’ resulted in the prediction of 2448 genomic objects, among which 2407 were protein-coding sequences. This reconstructed genome was relatively streamlined, with coding sequences covering approximately 90.87% of the entire genome. However, slightly different results were obtained with other annotation software: 2271 CDSs were found with RAST (745/1526 were not integrated to subsystem categories), 2261 CDSs with Prokka, and 2243 CDSs with Dfast. 

Sequence of MXMAG2 consisted of 203 contigs with an overall size of 1,040,932 bp and a G + C content of 57.78 mol%. CheckM estimated the genome to be 48.2% complete based on the presence of single-copy marker genes (82 markers were missing) and with 2.1% contamination (4 markers were duplicated). Annotation with MaGe of MXMAG2 resulted in the prediction of 1305 genomic objects, among which 1281 were protein-coding sequences. MXMAG2 had a relatively streamlined genome with coding sequences covering approximately 91.34% of the entire genome. However, slightly different results were obtained with other annotation software: 1149 CDSs with Prokka and 954 CDSs with Dfast. 

As for other *Methanomassiliicoccales* MAGs published previously, both MAGs did not contain one full complete 5S-16S-23S rRNA genes operon [79]. One and two 5S rRNA genes were found for MXMAG2 and *Ca.* ‘Methanomassiliicoccus armoricus MXMAG1’MAGs, respectively. A partial 542 bp 23S rRNA gene was found in MXMAG2. We detected 24 and 38 tRNA for MXMAG2 and *Ca.* ‘Methanomassiliicoccus armoricus MXMAG1’ MAGs, respectively, according to ARAGORN RNA finder (http://130.235.244.92/ARAGORN/) associated to 18 standard amino acids for *Ca.* ‘Methanomassiliicoccus armoricus MXMAG1’. Cysteine and tryptophan tRNA appeared to be missing, although the CDSs of the cysteine-tRNA and tryptophan-tRNA ligases were found. 

The two *Methanomassiliicoccales* MAGs reconstructed did not contain any 16S rRNA sequences, probably due to the high degree of conservation of this gene and the closeness between 16S rRNA sequences of related organisms which make their binning challenging [91]. However, two 16S rRNA sequences referenced as k119_35148 and k119_91804 (1448 and 1397 bp, respectively) were independently reconstructed from the metagenomes and assigned to *Methanomassiliicoccales* (Table S3). Thus, it seems reasonable to assume that they correspond to the same 2 *Methanomassiliicoccales* genomes reflected by the MAGs. The similarity of the 16S rRNA gene sequence with the sequences of the closest cultured relatives, calculated using the “16-based ID” tool provided by the EzBioCloud web server (https://www.ezbiocloud.net/) showed that the sequence k119_35148 (probably the one corresponding to *Ca.* ‘Methanomassiliicoccus armoricus MXMAG1’) was quite closely related to *M. luminyensis* (98.23% pairwise similarity) and *Ca.* ‘M. intestinalis’ (97.61%), indicating the enrichment of an undescribed representative of *Methanomassiliicoccus*. This sequence, even if it diverges at a nucleotide base (probably due to a sequencing error in the 16S rRNA gene sequence extracted from the metagenome), most likely corresponds to that of OTU21 present in the metabarcode dataset throughout the PENF cultivation period, and also present in natural samples KERG, MOUG3, MOZ1–3, PAV60, and PENF. Since the level of similarity of the 16S rRNA gene sequence between sequence k119_35148 and those of its closest relatives was below the threshold value (98.7–99%) currently recommended for testing the genomic uniqueness of a new species, this value alone was sufficient to conclude that *Archaea* forming a new genomic species within the genus *Methanomassiliicoccus* have been enriched in this culture [92,93]. These results were congruent with the ANI and digital DDH scores calculated on *Ca.* ‘Methanomassiliicoccus armoricus MXMAG1’, reinforcing the idea that this sequence corresponds well to the *Ca.* ‘M. armoricus MXMAG1’. The nearly complete sequence of the 16S rRNA gene bearing the reference k119_91804 (probably the one corresponding to MXMAG2), covered completely identically the representative sequence OTU101 (Figure 2), which was present in the environmental samples KERG, MOZ1–3, PAV70, PAV80, and PENF. This sequence was more distant from the sequences of the species and genera of *Methanomassiliicoccales* described to date, with a pairwise similarity ranging from 88.96 to 91.84%, respectively, to the closest relatives *Ca.* ‘Methanogranum caenicola’ and *M. luminyensis*, respectively. Although these sequences were incomplete, these 16S rRNA gene sequence similarity scores are far below the standard threshold level (<94.5%) generally accepted for the delineation of a new genus [93]. Thus, an archaeon belonging to an undescribed genus of *Methanomassiliicoccales*, which would be widely distributed in natural ecosystems, has likely been enriched in this culture-based incubation experiment.

#### 3.3.2. Putative Metabolic Pathways

**Figure 5:**
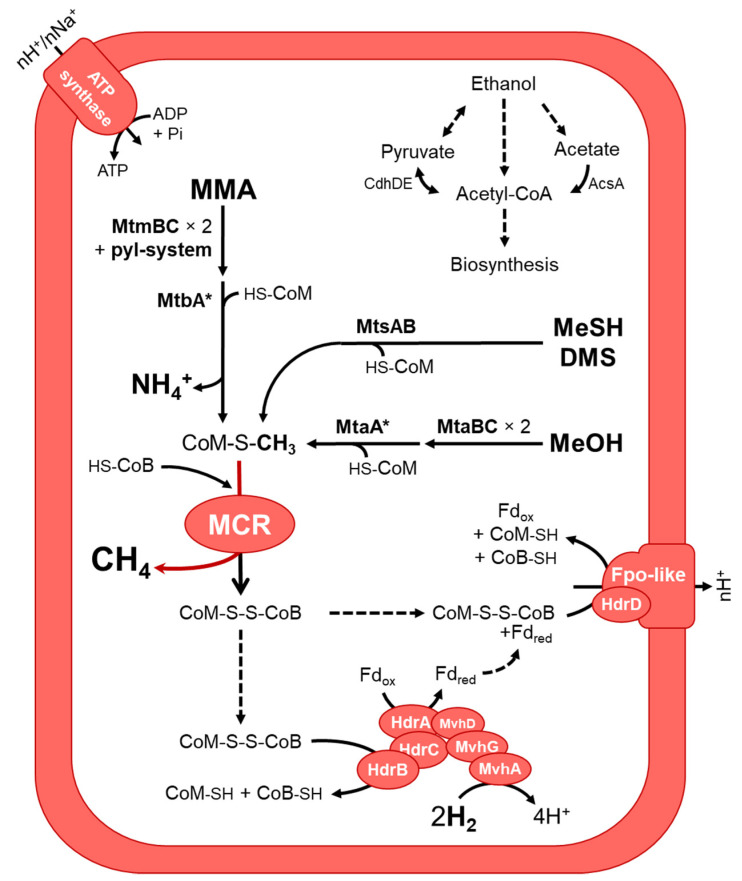
Predicted metabolic pathways for methanogenesis, carbon assimilation, and energy conservation in *Ca.* ‘Methanomassiliicoccus armoricus MXMAG1’, belonging to the ‘free-living clade’ of *Methanomassiliicoccales*. Genes encoding the MtmBC and MtaBC enzymes were found in two copies in the MAG. The “*” symbol indicates the presence of enzyme homologous to the MtaA/MtbA which are found in other *Methanomassiliicoccales* for MeOH and MMA incorporation, as described elsewhere [22]. Legend: CdhDE, Acetyl-CoA decarbonylase/synthase complex subunits; DMS, dimethylsulfide; MeOH, methanol; MeSH, methanethiol, MMA, monomethylamine.

An extensive genomic characterization has been performed on the high-quality MXMAG1 *Ca.* ‘Methanomassiliicoccus armoricus MXMAG1’ to explore the pathways of methanogenesis, the biology, and the possible adaptations of the ‘free-living’ clade *Methanomassiliicoccales Archaea* from which this MAG originates, which appear to be widespread in natural ecosystems (Figure 5). 

Consistent with previous studies conducted on *Methanomassiliicoccales*, the methyl-dependent hydrogenotrophic methanogenesis pathway was predicted in *Ca.* ‘Methanomassiliicoccus armoricus MXMAG1’ MAG. Hence, the metabolism of the *Archaea*, from which this MAG was derived, was also probably similar to the other described *Methanomassiliicoccales* [16,22,23,26]. Methyltransferases for the production of methane from methanol (MtaA/MtaBC), monomethylamine (MtbA/MtmBC), dimethylsufide and methanethiol (MtsAB) and HdrABC-MvhAGD electron-bifurcating complex (including an (NiFe)-hydrogenase) have been identified, suggesting that *Ca.* ‘Methanomassiliicoccus armoricus MXMAG1’ would be capable of reducing such methyl-compounds using H_2_ as electron donor. As for *Ca.* ‘Methanoplasma termitum’ [26], the genes encoding the methyltransferases involved in the use of di- and trimethylamine (*mtbB* and *mttB*, respectively) are absent from the sequence of *Ca.* ‘M. armoricus MXMAG1’. However, several non-binned *Methanomassiliicoccales*-affiliated *mttB* sequences have been found in the metagenome (Figure S4), suggesting that some *Methanomassiliicoccales* in this culture, and possibly even this one, were capable of reducing methyl-groups of TMA into methane. As in *Methanomassiliicoccales* from digestive environments, the presence of a complete pyl system (pylBCD/pylS), involved in the biosynthesis of the 22nd amino acid pyrrolysine, was found, and the presence of the amber codon was found in methyltransferase genes, as in other *Methanomassiliicoccales* methylamine methyltransferases [16,94]. A complete methyl-coenzyme M reductase (MCR) complex (*mcrABG*) gene set was present in the MAG. Of the *N*^5^-methyltetrahydromethanopterin: coenzyme M methyltransferase (MTR) complex, only *mtrH* gene was present, and none of the genes of the methyl branch of the H_4_MPT-type Wood-Ljungdahl pathway. Altogether, the presence of the identified genes in MXMAG1 strongly suggests that *Ca.* ‘M. armoricus’ produced energy from a methyl-dependent methanogenesis. 

As described in all known *Methanomassiliicoccales*, *Ca.* ‘Methanomassiliicoccus armoricus MXMAG1’ harbored two heterodisulfide reductases systems (the methyl viologen-dependent hydrogenase/heterodisulfide reductase (MVR/HDR) complex (MvhADG/HdrABC) and the HdrD-liked bonded to the Fpo-like complex) involved in the regeneration of the heterodisulfide complex (CoM-S-S-CoB) and the energy-conserving [23,26,95]. *Ca.* ‘M. armoricus MXMAG1’ encoded also an ADP-forming acetyl-CoA synthetase (AscA) [13,23,26], suggesting that acetate could be used as a carbon source. The MAG sequence also encodes the enzymes that allow the synthesis of acetyl-CoA from pyruvate (a pyruvate synthase complex; a pyruvate dehydrogenase complex), and a complete ethanol degradation pathway to acetyl-CoA, suggesting that pyruvate and ethanol may also serve as a carbon source for cellular biosynthesis.

Fourteen out of the 22 proteinogenic amino acid biosynthesis pathways were predicted as complete in *Ca.* ‘Methanomassiliicoccus armoricus MXMAG1’ MAG: Ala, Arg, Asn, Asp, Cys, Glu, Gly, His, Ile, Lys (biosynthesis VI), Pyl, Thr, Trp, and Val. The presence of numerous genes coding for cobalamin (vitamin B12) ATP-binding transporters involved in cobalamin import (BtuD) suggests that this genomic strain was capable to import this vitamin. Its de novo synthesis pathway is not present on the 94.4% complete reconstructed MAG. Several complete central metabolic pathways were also identified in the MAG, including: (i) gluconeogenesis/glycolysis, (ii) anaplerotic CO_2_ fixation into oxaloacetate, (iii) the non-oxidative branch of the pentose phosphate pathway, and (iv) a fatty acid activation pathway. The presence of these complete paths, as well as those mentioned above, does not necessary imply that all of them were used.

The poor quality of the MXMAG2 MAG reconstruction allowed only partial metabolic pathways to be predicted. This MAG had genes encoding a nearly complete MCR complex (the *mcrB* subunit was missing) and several genes encoding subunits of a Fpo-like complex (*fpoMN*) and an MVR/HDR complex (*mvhAB* and *hdrBC*, respectively). The presence of the *mtbC* gene coding for the dimethyltransferase suggests that DMA could be used for methanogenesis. The presence of the *Methanomassiliicoccales mtaA*/*mtbA* gene encoding for a methyltransferase involved in methanol and methylamines incorporation was also in line with this assumption [22]. Unlike the high-quality MAG, the MXMAG2 encoded a choline trimethylamine-lyase activating enzyme (*cutD*). This enzyme may be involved in the production of TMA from choline, possibly allowing the utilization of choline as substrate for methanogenesis. As described in *Ca.* ‘Methanomassiliicoccus armoricus MXMAG1’, this MAG encoded the ADP-forming acetyl-CoA synthetase, as well as the pyruvate dehydrogenase complex.

#### 3.3.3. Putative Physiological and Stress Response Functions

Several putative adaptive mechanisms known to be involved in microbial stress responses have been identified in *Ca.* ‘Methanomassiliicoccus armoricus MXMAG1’ MAG. Among them, complete biosynthesis and degradation pathways of trehalose were predicted, as well as 8 genes coding for a sub-unit (*malK*) of a trehalose/maltose importer. This could suggest that trehalose might be used to cope with external factors of the environment, such as temperatures, dehydration, or osmotic/ oxidative stresses, or might be used as a storage compound, as described in other prokaryotes [96,97]. However, functional analyses will be useful to confirm this hypothesis.

As described in many microbial lineages including some methanogens, *Ca.* ‘M. armoricus MXMAG1’ MAG encoded a pyruvoyl-dependent arginine decarboxylase [98,99]. The presence of the gene encoding this enzyme suggests that *Ca.* ‘M. armoricus MXMAG1’ might regulate intracellular pH by converting a L-arginine to agmatine and CO_2_ under acidic conditions [100]. In addition, this MAG encodes enzymes involved in (i) the degradation of agmatine to putrescine via an agmatinase; (ii) possessed a complete pathway for putrescine biosynthesis; and (iii) possessed numerous copies of the gene encoding the pH-dependent bidirectional putrescine-ornithine transporter PotE [100,101]. Putrescine is a common polyamine that could promote the growth of most living cells. Although present in most methanogenic archaea, the function of putrescine remains unclear [102,103]. The presence of complete pathways involved in putrescine incorporation and biosynthesis suggests that this compound might play a role in *Ca.* ‘M. armoricus MXMAG1’ physiology.

Several genes involved in arsenic metabolism were also found in this MAG. Arsenic is a harmful toxic pollutant at low concentrations that is widely distributed in natural environments (i.e., groundwater, sediments, soils), with the potential to inhibit methanogenesis depending on arsenic speciation and concentration [104,105]. *Ca.* ‘Methanomassiliicoccus armoricus MXMAG1’ genome contains a complete *arsRDABC* operon, similar to the one found in *Escherichia coli* [106]. The *arsRDABC* operon is involved in the arsenic detoxication and the active release of this compound outside the cell. The only difference with the canonical arsenic detoxification pathway is that the *arsB* gene is replaced in MXMAG1 by the *acr*3 gene, which codes for a protein with the same function as ArsB (arsenite efflux pump). It has been shown that various *Bacteria* and *Archaea* prevent arsenic toxicity by using this pathway which, ironically, convert arsenate to the more toxic arsenite [106,107,108]. It should be noted that some MAGs of the sister lineage of *Methanomassiliicoccales* named *Candidatus* ‘Gimiplasmatales’ have also been reported to encode genes of this operon [109]. Interestingly, a comparative genomic analysis conducted on 5 genomes/MAGs belonging to the ‘host-associated’ clade and on 20 genomes/MAGs belonging to the ‘free-living’ clade showed that the complete *arsRDABC* operon was only present in MXMAG1 and MXMAG2. Several genes of this operon were also present in the genome of *M. luminyensis* strain B10^T^ and in the MAG referenced as RumEn M1. Genes coding for the arsenical pump-driving ATPase were not present in the genomes/MAGs from the host-associated clade analysed in this study. Furthermore, genes involved in arsenic methylation (e.g., *arsM*) by other methanogens were not found in *Ca.* ‘M. armoricus MXMAG1’ MAG nor in *Methanomassiliicoccales* genome comparisons [110]. Thus, reduction and excretion of arsenite could represents the only arsenic detoxification pathway available for *Methanomassiliicoccales*. It is noteworthy that the detoxication pathway of arsenate to arsenite was also present in MXMAG2. Finally, in opposition to *M. luminyensis*, *Ca.* ‘Methanomassiliicoccus armoricus MXMAG1’ and *Ca.* ‘Methanomassiliicoccus intestinalis’ do not harbor the *hgcAB* operon [24,111]. This operon encodes an enzyme involved in the methylation of inorganic mercury (Hg) to form the highly toxic methylmercury [112,113]. Among methanogens, this metabolic capacity has only been confirmed for *M. luminyensis* and 7 species from various origins distributed among the *Methanomicrobia* class [113]. Therefore, if confirmed by functional approaches on isolates, this would demonstrate that this ability would not be uniformly present within the genus *Methanomassiliicoccus*, but only in some species of the genus.

### 3.4. Co-Occurrence Network Analysis

**Figure 6:**
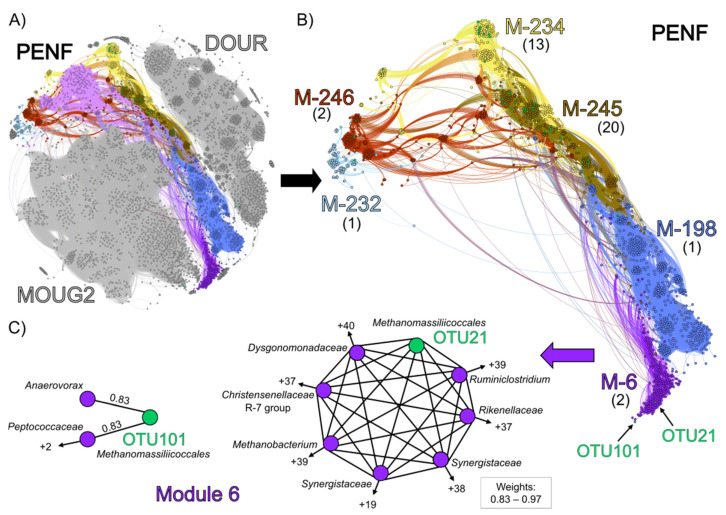
Co-occurrence network reconstructed based on 16S rRNA gene-sequencing data from substrate-amended slurries (DOUR, PENF, and MOUG2, respectively), based on a Spearman rank’s correlation between OTUs and calculated with a percolation threshold of 0.79. (**A**) Clusters in slurries DOUR, PENF, and MOUG2, respectively. Modules in PENF network are colored to highlight them; (**B**) modules (M-6, module 6; M-198, module 198; M-232, module 232; M-234, module 234 and M-245, module 245, respectively) containing *Methanomassiliicoccales* in the cluster from the PENF culture-based incubation experiment. Modules consisting of a single *Methanomassiliicoccales* (modules 22 and 68, respectively) were excluded. The number of *Methanomassiliicoccales* in each module is shown in brackets; (**C**) non-random co-occurrences between *Methanomassiliicoccales* and other prokaryotes in module 6 showing co-occurrences of *Methanomassiliicoccales* (OTU21 and OTU101, respectively) with putative acetate- and H_2_-producing bacteria. Black arrows followed by a plus and a number indicate the number of taxa non-randomly co-occurring with other prokaryotes outside the groups represented. The weights of the co-occurrences are written above the link (OTU101) or in the insert below the cluster of the OTU21.

To explore potential interactions between *Methanomassiliicoccales* and other microorganisms, co-occurrence network analyses of 16S rRNA genes sequences were performed. These analyses were carried out on the 22 environmental samples and on culture-based incubation experiments performed with high substrate concentrations (Figure 6). 

Four modules were identified, each of which comprised *Methanomassiliicoccales* sequences sharing strong non-random associations (weight >0.7) with other taxa: module 1104 (OTU21), module 117 (OTU101), module 0 (OTU30), and module 927 (OTU926) (Table S5). Module 117 included 119 other OTUs distributed in more than 20 order-level lineages. Among others, this module contained 8 *Woesearchaeales*, 7 *Aminicenantales*, 18 *Omnitrophales*, and 13 Marine Benthic Group D/Deep-sea Hydrothermal Vent Euryarchaeota Group-1 (MBGD/DHVEG-1), as well as 3 *Methanofastidiosales* (hydrogenotrophic methyl-dependent methanogenesis) and a *Methanothermus* (*Methanobacteriales*; hydrogenotrophic methanogenesis). Interestingly, *Woesearchaeotales* and *Aminicentales* present in this module have recently been proposed to maintain a nutritional relationship with various hydrogenotrophic methanogens, notably *Methanobacteria*, *Methanofastidiosales*, *Methanomicrobia*, and/or *Methanomassiliicoccales* [34,114,115,116]. When using a percolation threshold with a high-value of 0.83, OTU101 showed the highest non-random co-occurrence with two MBGD/DHVEG-1 sequences (weights: 0.85 and 0.87, respectively), and OTU30 showed significant non-random-co-occurrences with the poorly described bacterial phylum NKB15 and with a *Microgenomatia* (weights: 0.84 and 0.85, respectively) (Table S5). At this threshold of 0.83, no OTU was branched with OTU21 or OTU926. Thus, to observe their associated connectivity, a slightly lower threshold was used (0.7) (Figure S5) [117]. At this value, the *Methanomassiliicoccales* OTUs 21, 30, 101, and 926 showed significant non-random co-occurrences (weights: 0.70–0.87) with, respectively, 3, 5, 6, and 11 OTUs. Under these conditions, *Anaerolineaceae*, *Anaerovorax*, *Aminicenantales*, *Geobacteraceae*, MBGD/DHVEG-1, *Sporomusaceae*, and *Woesearchaeales* were the main lineages sharing strong connections with *Methanomassiliicoccales*. Interestingly, *Anaerolineaceae*, *Anaerovorax*, *Geobacteraceae*, and *Sporomusaceae* have also been reported to form syntrophic relationships with acetoclastic and hydrogenotrophic methanogens, notably *Methanobacteriales*, *Methanofastidiosales*, and *Methanomassiliicoccales* [118,119,120,121,122]. In contrast, if active, the *Desulfatiglans* and the *Methanoregulaceae* (*Methanomicrobia*) associated with OTU30 and OTU926, respectively, could represent hypothetical competitors for the acetate and/or H_2_ resources of *Methanomassiliicoccales* [123,124,125]. 

Similarly, a co-occurrence network analysis was also performed (percolation threshold: 0.79) on the substrate-amended slurries incubated over 10 weeks (DOUR, MOUG2 and PENF) to reveal the narrow potential interactions between *Methanomassiliicoccales* and other microbial taxa (Figure 6). Our aim was to assess whether the microbial composition of the cultured community could be likely correlated with the enrichment of *Methanomassiliicoccales* in the PENF slurry. The overall topology of the network highlighted 3 clusters of nodes that clearly corresponded to the different culture-based incubation experiments (Figure 6A): (i) the cluster of nodes over-represented in the Dourduff-en-Mer slurry sampled throughout the incubation, is located at the top of the network, (ii) the cluster of nodes over-represented in the Mougau samples is located at the bottom, and (iii) the cluster of nodes over-represented in the Penfeld samples is located in between. The modularity analysis showed that 40 of the 42 the *Methanomassiliicoccales*-affiliated OTUs were detected inside modules from the Penfeld Cluster. This is congruent with the absence of *Methanomassiliicoccales* detected by metabarcoding approach in the DOURD and MOUG2 incubated slurries (Figure S1). In terms of overall community composition (Figure 6B), modules 232, 234, 245, and 246 showed close taxonomic patterns dominated by *Firmicutes* and *Bacteroidetes*, taxa similar to those found in animal GITs from which most of the known *Methanomassiliicoccales* have been enriched or isolated so far [20,21,22,26]. These modules were predominantly composed of *Bacteroidales* (*Dysgonomonadaceae* and *Rikenellaceae*) and various *Clostridiales* (*Christensenellaceae* R-7 group, *Gracilibacteraceae*, *Hungateiclostridiaceae*, *Oscillospiraceae*, *Ruminococcaceae*) mainly reported to be able to produce acetate and/or H_2_ as fermentative end products and sometimes to form partnerships with hydrogen-consuming methanogens (i.e., *Methanospirillum hungatei*, *Methanobrevibacter smithii*) (Table S5) [82,126,127,128,129,130,131,132]. Such bacteria pattern could possibly be explained by the utilization of a medium rich in organic compounds which could be used as substrates for fermentation or acetogenesis. As we used a complex culture medium comprising various substrates for methanogenesis, several methanogenic *Archaea* mainly affiliated to *Methanobacteriales*, *Methanomicrobiales*, and/or *Methanosarcinales* were maintained throughout the incubation process and were also found in these modules. Despite strong similarities in microbial community composition with the four other modules (modules 232, 234, 245 and 246), module 6 contained a slightly higher taxonomic diversity among *Firmicutes*. This module contained the OTU21 and OTU101, which co-occurred strongly with *Ca.* ‘Methanomassiliicoccus armoricus MXMAG1’ and MXMAG2, respectively. Since these OTUs were predominant throughout the PENF culture-based incubation experiment and were also present in various environmental samples examined in this study, we focused on these two OTUs to determine whether *Methanomassiliicoccales* would significantly co-occur with given taxa (Figure 6C). OTU101 showed high non-random associations with an *Anaerovoracaceae* OTU (*Peptostreptococcales*-*Tissierellales*) (weight: 0.83) and a *Peptococcaceae* OTU (weight: 0.82). Several representatives from these lineages have been reported to ferment amino acids and to produce acetate or H_2_ and to carry out a functional or syntrophic relationships with hydrogenotrophic methanogens that scavenge hydrogen to keep fermentation of fatty acids and alcohols energetically favorable (e.g., *Methanospirillum hungatei*) (Table S5) [118,120,133,134]. OTU21 showed strong non-random co-occurrences with 43 taxa (weights: 0.79–0.95). The six strongest non-random associations (weights: >0.9) occurred with taxa known to produce acetate and dihydrogen by fermentation: A *Dysgonomonadaceae* (weight: 0.96), two *Synergistaceae* (weights: 0.95 and 0.93), a *Ruminiclostridium* (weight: 0.95), a *Christensenellaceae* R-7 group (weight: 0.92) and a *Rikenellaceae* (weight: 0.91). Members of the *Synergistaceae* family and the *Ruminiclostridium* genus have been reported to be able to produce short-chain fatty acids from several proteins and peptides, and acetate from various complex substrates (Table S5) [135,136]. A representative of the genus *Methanobacterium,* potentially a competitor for H_2_, was also strongly associated with OTU21 (weight: 0.91). 

**Figure 7:**
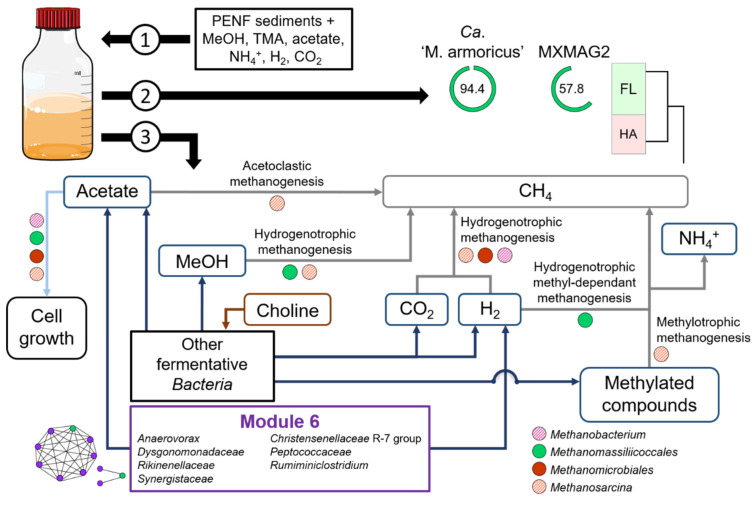
Schematic diagram representing the substrates amended to the original slurry in the culture-based incubation experiment PENF (1) which led to the reconstruction of two *Methanomassiliicoccales* MAGs (*Ca.* ‘M. armoricus MXMAG1′ and MXMAG2) affiliated to the ‘free-living’ clade (2). Tentative reconstruction of the microbial metabolic interactions and substrate functions in this culture-based incubation experiment (3). The purple frame represents the module 6 of the co-occurrence network analysis, composed of reported fermentative *Bacteria* which maintained strong non-random co-occurrences with the dominant *Methanomassiliicoccales* OTUs sequences; FL: ‘Free-living’ clade; HA: ‘Host-associated’ clade; numbers in green rings indicate the completeness of the two *Methanomassiliicoccales* MAGs; dark blue arrows indicate fermentative products; grey arrows indicate the methanogenesis pathways probably occurring in this slurry; brown arrow indicates a fermentative substrate found in the enrichment slurry; light blue arrow indicates that acetate is potentially consume for methanogens cell growth. The colored circles indicate the dominant methanogenic taxa during the enrichment process and their known metabolic properties. The hatched color indicates that this property is present in only a part of the taxa of the lineage.

The results of these network analyses reinforce the hypothesis that *Methanomassiliicoccales* of the ‘free-living’ clade appear to display strong co-occurrences with acetate- and H_2_-producing bacteria, suggesting that they might maintain a metabolic interaction similar to that described between fermentative bacteria and other hydrogen-utilizing methanogens, by scavenging H_2_ and promoting acetate production [136,137,138] (Figure 7). 

Based on large-scale metagenome analysis, co-occurrence between the *Methanomassiliicoccales* genera *Ca.* “Methanomethylophilus” and *Methanomassiliicoccus* with various TMA-producing *Bacteria* in a GIT context was recently proposed, supporting the hypothesis of an interaction between these taxa and gut bacteria to earn TMA and H_2_ [26]. However, metabolic interactions, such as those suggested here as a hypothesis, have also recently been highlighted between *Ca.* ‘Methanomethylophilaceae’ and various acetogenic *Bacteria* in another GIT context [111]. Thus, these results reinforce the idea that *Methanomassiliicoccales* could develop relationships with acetogenic *Bacteria*, possibly to access acetate for cell growth in both GIT and environmental contexts [12,44]. Nevertheless, it cannot be excluded that another type of functional interaction would be at work between *Methanomassiliicoccales* and their associated microbial cohorts. One or more other metabolite(s) produced by associated microorganisms could stimulate the growth of *Methanomassiliicoccales* [24,26]. It also cannot be excluded that the interaction promoting the growth of *Methanomassiliicoccales* is simply functional/metabolic. Finally, a strong metabolic relationship, close to syntrophy, based on H_2_ moderation between *Methanomassiliicoccales* and various fermentative bacteria could explain why the isolation of *Methanomassiliicoccales* representatives in clonal culture, from natural environments, has not been successful so far.

## 4. Conclusions

Among the diverse marine and terrestrial ecosystem samples screened in this study, *Methanomassiliicoccales* never appeared to represent a significant fraction of the microbial diversity. Nevertheless, cultural approaches allowed us to obtain a slurry significantly enriched in *Methanomassiliicoccales*, from freshwater sediments. This enrichment allowed the reconstruction of a high-quality MAG (MXMAG1) corresponding to a new candidate genomic species named *Candidatus* ‘Methanomassiliicoccus armoricus MXMAG1’. This genomic species seems to be present in several ecosystems and is affiliated to the ‘free-living’ clade of *Methanomassiliicoccales*. Analysis of its genome showed that the archaea from which this genome is derived have probably an energy-producing metabolism similar to previously described *Methanomassiliicoccales*, based on the consumption of methylated compounds and hydrogen to produce methane. Furthermore, the comparison of this MAG with previously published genomes of the two functional clades of *Methanomassiliicoccales* revealed that representatives from the ‘free-living’ clade, to which MXMAG1 belongs, encoded for predicted various mechanisms involved in intracellular equilibrium to counteract certain environmental parameters. Finally, network analyses showed that *Methanomassiliicoccales* from the ‘free-living’ clade were strongly co-occurring with some putative acetate and dihydrogen-producing bacteria previously reported to form syntrophic relationships with methanogens in animal GITs.

## Figures and Tables

**Table 1:** Origin and main characteristics of the environmental samples selected after a pre-screening molecular stage targeting *Methanomassiliicoccales*. Environmental parameters were measured in situ during cruises/expeditions. –, not determined.

Sample Acronym	Sample Type	Geographical Origin	Water Depth (m)	Depth Below Subsurface/Subseafloor (cm)	pH	Temperature (°C)
**COMRA**	Deep-sea sediments	South-West Indian ocean	2267	0–20	7.5	–
**DOUR**	Coastal sediments	France (Bay of Morlaix, France)	0	5–10	7.25	8
**HM1**	Mud from a mud volcano	Barents sea, Norway	1285	0–1	–	–
**HM2**	Mud from a mud volcano	Barents sea, Norway	1285	1–6	–	–
**HM3**	Mud from a mud volcano	Barents sea, Norway	1285	6–11	–	–
**HM4**	Mud from a mud volcano	Barents sea, Norway	1282	1–6	–	–
**KERG**	Water from a geothermal hot spring	Kerguelen Island, Indian Ocean (French Southern and Antarctic lands)	–	–	7.0	50
**KRY150**	Deep-Sea Hypersaline Anoxic Basin	Mediterranean sea (Kryos basin)	~3338	–	7.5	15
**KRY238**	Deep-Sea Hypersaline Anoxic Basin	Mediterranean sea (Kryos basin)	~3338	–	6.5	15
**MOUG1**	Peatland soil	Peatland in France (Commana, France)	–	0–10	6.4	4
**MOUG2**	Peatland soil	Peatland in France (Commana, France)	–	–	4.78	2
**MOUG3**	Peatland soil	Peatland in France (Commana, France)	–	0–10	5.22	6
**MOUG4**	Peatland soil	Peatland in France (Commana, France)	–	0–10	4.75	2.3
**MOUG5**	Freshwater sediments	Peatland in France (Commana, France)	0.2	5–15	6.7	8
**MOZ1**	Sediments from a pockmarck area	Mozambique Channel (Madagascar shore)	762	2–4	8.0	8
**MOZ2**	Sediments from a pockmarck area	Mozambique Channel (Madagascar shore)	762	4–6	8.0	8
**MOZ3**	Sediments from a pockmarck area	Mozambique Channel (Madagascar shore)	762	6–11	8.0	8
**PAV60**	Anoxic water from a meromictic lake	Pavin lake (France)	60	–	5.3	4.2
**PAV70**	Anoxic water from a meromictic lake	Pavin lake (France)	70	–	5.3	4.75
**PAV80**	Anoxic water from a meromictic lake	Pavin lake (France)	80	–	5.3	5
**PENF**	Freshwater sediments	Tributary of the French river Penfeld (Brest, France)	0.15	0–5	6.0	8
**XIA**	Water from a geothermal hot spring	Hot spring in China (Xiamen Botanical Garden)	–	0	6.3	82

## Supplementary Materials

The following are available online at https://www.mdpi.com/2076-2607/9/1/30/s1: Supplementary Material: Text S1. Pre-metabarcoding screening. Text S2. Origin and detailed description of environmental samples. Text S3. Total DNA extraction for metabarcoding screening. Text S4. Details of the barcoding and the phylogenetic analyses. Text S5. Media composition. Text S6. Quantification of substrates and metabolic products. Supplementary Tables and Figures: Figure S1. Trends in microbial diversity revealed by metabarcoding in DOUR (top) and MOUG2 (bottom) substrate-amended slurries over time. Figure S2. Evolution of the prokaryotic alpha-diversity during the incubation of the PENF sample, in the culture-based incubation experiment. Figure S3. Microbial diversity patterns revealed by metabarcoding (16S rRNA gene copy numbers) in the PENF substrate-amended slurry over time. Figure S4. Phylogenetic tree showing the relationships between the trimethylamine methyltransferases (*mttB*) sequences extracted from the metagenome (in red), and closest *mttB* sequences in public databases. Figure S5. Co-occurrence network reconstructed based on 16S rRNA gene sequencing data from bulk environmental samples, based on a Spearman rank’s correlation between OTUs and calculated with a percolation threshold of 0.70. Table S1. Origin and location of the 86 environmental samples screened as a first approach for the research of *Methanomassiliicoccales*. Table S2. Abundance of *Methanomassiliiccoccales*, other methanogens and total prokaryotic OTU 16S rRNA, representative sequences generated by metabarcoding sequencing in environmental samples and enrichment cultures PENF, DOUR, and MOUG2. Table S3. List of the *Methanomassiliicoccales* 16S rRNA gene sequences generated by metagenomics sequencing from the PENF enrichment culture (8th week of incubation), and 16S rRNA gene representative sequences of OTUs generated by metabarcoding sequencing. Table S4. Results of the quantification of *Methanomassiliicoccales* by qPCR in the different samples. Table S5. Taxonomic assignation of the taxa non-randomly associated with *Methanomassiliicoccales* OTUs and their reported metabolic features, within the PENF substrate-amended slurry.

## Abbreviations

ADP	Adenosine diphosphate
Ala	Alanine
ANI	Average Nucleotide Identity
ANOVA	Analysis of variance
Arg	Arginine
ASCII	American Standard Code for Information Interchange
ATP	Adenosine triphosphate
Asp	Asparagine
BIOM	Biological Observation Matrix
Bp	Base pair
BSA	Bovine Serum Albumin Acetylated
*Ca.*	*Candidatus*
CERS	Cation Electrolytically Regenerated Suppressor
CDS	Coding DNA sequences
CSS	Cumulative Sum Scaling
Cys	Cystine
DES	Deionized Elution Buffer
DDH	DNA-DNA Hybridization
Dfast	DDBJ Fast Annotation and Submission Tool
DHAB	Deep-sea Hypersaline Anoxic Brine
DHVEG-1	Deep-sea Hydrothermal Vent Euryarchaeota Group-1
DMA	Dimethylamine
DMS	Dimethylsulfide
DNA	Desoxyribonucleic Acid
DSMZ	Deutsche Sammlung von Mikroorganismen und Zellkulturen
EB	Elution Buffer
FID	Flame Ionization Detector
GC	Gas Chromatograph
GGDC	Genome to Genome Distance Calculator
GIT	Gastro-Intestinal Tract
Glu	Glutamine
Gly	Glycine
GTDB-Tk	Genome Taxonomy Database Toolkit
HDR	HeteroDisulfide Reductase
His	Histidine
Ile	Isoleucine
iTOL	interactive Tree Of Life
KEGG	Kyoto Encyclopedia of Genes and Genomes
Lys	Lysine
MAG	Metagenome-Assembled Genome
MaGe	MicroScope Microbial Genome Annotation and Analysis Platform
MBGD	Marine Benthic Group D
MCR	Methyl-Coenzyme M Reductase
MeOH	Methanol
MeSH	Methanethiol
MetaBAT	Metagenome Binning with Abundance and Tetra-nucleotide frequencies
MMA	Monomethylamine
MUSCLE	Multiple Sequence Comparison by Log- Expectation
NCBI	National Center for Biotechnology Information
OTU	Operational Taxonomic Unit
PAMELA	Passive Margins Exploration Laboratories
PCI	Phenol Chloroform Isoamyl alcohol
PCR	Polymerase Chain Reaction
Pyl	Pyrrolysine
Prokka	rapid prokaryotic genome annotation
qPCR	Quantitative Polymerase Chain Reaction
Quast	Quality Assessment Tool
RAST	Rapid Annotations using Subsystems Technology
RDP	Ribosomal Database Project
rRNA	ribosomal Ribonucleic Acid
SDS	Sodium Dodecyl Sulfate
SSU	Small Subunit
TE	Tris-Ethylene diamine tetra acetic acid
Thr	Threonine
TMA	Trimethylamine
tRNA	transfer RNA
Trp	Tryptophane
UniProtKB	Universal Protein resource Knowledgebase
Val	Valine
