# Supplementary material for: New Insights into the Ecology and Physiology of Methanomassiliicoccales from Terrestrial and Aquatic Environments
Source: Microorganisms. 2020 Dec 24;9(1):30. doi: 10.3390/microorganisms9010030 (PMC7824343; doi:10.3390/microorganisms9010030)
Supplement: Supplementary file 1 [file microorganisms-09-00030-s001.pdf]

microorganisms

Supplementary Material

New insights into the ecology and physiology of *Methanomassiliicoccales* from terrestrial and aquatic environments.

Marc Cozannet[1], Guillaume Borrel[2], Erwan Roussel[1], Yann Moalic[1], Maxime Allioux[1], Amandine Sanvoisin[1], Laurent Toffin[1] & Karine Alain[1,*]

[1] Univ Brest, CNRS, IFREMER, IRP 1211 MicrobSea, Laboratoire de Microbiologie des Environnements Extrêmes LM2E, UMR 6197, IUEM, Rue Dumont d'Urville, F-29280 Plouzané, France; Marc.Cozannet@univ-brest.fr (M.C.); Erwan.Roussel@ifremer.fr (E.R.); Yann.Moalic@univ-brest.fr (Y.M.); Maxime.Allioux@univ-brest.fr (M.A.); Laurent.Toffin@ifremer.fr (L.T.); Karine.Alain@univ-brest.fr (K.A.)

[2] Department of Microbiology, Unit Evolutionary Biology of the Microbial Cell, Institut Pasteur, Paris, France; guillaume.borrel@pasteur.fr (G.B.)

* Correspondence: Karine.Alain@univ-brest.fr; Tel.: +33-0298-4988-53

Received: date; Accepted: date; Published: date

Supplementary Text

Text S1. Pre-metabarcoding screening

Section 1.1. Total DNA extraction

In order to search for natural samples containing *Methanomassiliicoccales*, an initial large-scale molecular screening was carried out on a wide range of anoxic samples (86 in total).

DNA extractions were carried out in triplicate with a protocol adapted to each type of matrix. Two types of DNA extraction procedures have been implemented depending on the nature of the matrices: (i) Nucleic acids were extracted from sediments, mud and peatland soils by combining a mechanical lysis step to a commercial DNA extraction kit for soil type matrices. The first extraction step consisted of mechanical cell lysis of 0.5 g of sample by bead-beating (5500 rpm, 30 s), using the Precellys 24® tissue homogenizer (Bertin instruments, France). Then, the FastDNA™ SPIN Kit for Soil (MP Biomedicals, Thermo Fisher Scientific) was used following manufacturer's instructions. In order to optimize the precipitation of small amounts of DNA and to improve extraction yields, 10 µl of linear acrylamide (5 mg ml^{-1}, Ambion, Thermo Fisher Scientific) was added to the sample. After a chemical lysis step, DNA was purified on a silica column and eluted in 50 µl of DES buffer. (ii) Nucleic acids were extracted from liquid samples (brines, lake waters, geothermal hot spring fluids) using a phenol/chloroform/isoamyl alcohol (25/24/1; pH 8.0; Sigma-Aldrich) (PCI) DNA extraction protocol. Fifteen ml of sample were first centrifuged (20 min, 13 000 rpm, 4 °C) to pellet the cells. Cell pellets were then resuspended in 1 ml TE-Na-1X lysis buffer, with 10 µl of linear acrylamide (5 mg ml^{-1}). Cell lysis and deproteinization were performed by addition of 100 µl sarkosyl (10% w/v), 100 µl sodium dodecyl sulfate (SDS; 10% w/v) and 20 µl proteinase K (20 mg ml^{-1}, Thermo Fisher Scientific, Illkirch, France), followed by a 3h incubation at 55 °C in a water bath. After incubation, 1 volume of PCI was added to 1 volume of lysis solution, solutions were mixed by gentle inversion and then centrifuged (15 min, 13 000 rpm, 4 °C) to extract the nucleic acids. After centrifugation, the upper aqueous phase was collected and extracted with chloroform. After centrifugation (15 min, 13 000 rpm, 4 °C), the upper aqueous phase was collected and nucleic acid precipitation was carried out by addition of 40 µl sodium acetate (3 M, pH 5.2) and 0.8 volume of isopropanol. In order to favour nucleic acids precipitation, each tube was placed 30 min at –80 °C. After thawing at room temperature, the solutions were centrifuged (20 min, 13 000 rmp, 4 °C) to collect DNA pellets and these were washed with 70% (v/v) ethanol. Finally, DNA were resuspended in 50 µl TE-1X buffer.

Section 1.2. PCR amplifications of regions of 16S rRNA gene sequences with Methanomassiliicoccales-*specific primers*

In order to select samples containing *Methanomassiliicoccales*, a 478 bp fragment of the V4 region of *Methanomassiliicoccales* 16S rRNA gene was selectively amplified using primers AS1_Fw (5′-CAG CAG TCG CGA AAA CTT C-3′) [1] and AS2_Rv mod. (5′-AAC AAC TTC TCT CCG GCA CT-3′) (this study) (this primer corresponds to a modified version of the AS2 primer described elsewhere [1]). These *Methanomassiliicoccales*-targeting primers specificity were retested *in silico* using online software's ProbeMatch (https://rdp.cme.msu.edu/probematch/search.jsp) (RDP2, ribosomal database project 2) and TestPrime (https://www.arb-silva.de/search/testprime/) (against the 132 version of the SILVA SSU database). This primer pair covered 87% of the diversity of correctly affiliated *Methanomassiliicoccales* sequences of the SILVA 138 SSU database without any mismatch, and 100% of the *Methanomassiliicoccales* sequences by allowing 1 mismatch. *In vitro* analyses were also performed by using genomic DNA from *Methanomassiliicoccus luminyensis* B10^T (DSM 25720) strain (positive control) and two negative controls: *Methanococcoides vulcani* SLH33^T (DSM 26966) strain (a methylotrophic methanogenic *Archaea* belonging to the *Methanosarcinales* [2] and *Thermococcus* sp. strain (an archaeal species belonging to the non-methanogenic order *Thermococcales*) [3], respectively. Positive amplifications were obtained only when using *M. luminyensis* genomic DNA as a template. The reaction mixtures used for DNA amplifications, carried out in triplicates, contained, for a volume of 25 µl (for one reaction): 15.86 µl molecular biology grade water, 5 µl buffer (10× GreenGoTaq® buffer, Promega, USA), 1 µl of each primer (at 10 mM), 0.5 µl dNTPs (10 mM of each primer), 0.12 µl GoTaq® G2 DNA Polymerase (5 U µl^{-1}, Promega), 0.12 µl Bovine Serum Albumin Acetylated (BSA, 10 mg ml^{-1}, Promega), 0.4 µl $MgCl_2$ (10 mM, Thermo Fisher Scientific ™) and 1 µl of DNA template. Amplifications were performed in a PCR apparatus (GeneAmp®, PCR System 9700, Thermo Fisher Scientific™) using the following cycling program: 95 °C for 5 min, 34 to 40 cycles of 95 °C for 1 min, 60 °C for 1 min, 72 °C for 1 min, and a final extension period of 6 min at 72 °C. Genomic DNA solutions from *Methanomassiliicoccus luminyensis* were used as positive controls. Amplicons quality were then checked by electrophoretic migration. Twenty-two samples that led to a positive amplification of the sequences of interest (in ≥ 2 replicates) were selected for further investigation.

Text S2. Origin and detailed description of environmental samples

The eighty-six samples collected from anoxic zones of various ecosystems (marine sediments, deep-sea hydrothermal vents, marine pockmarks, submarine mud volcano, deep-sea hypersaline anoxic brine, peatland soils, lakes, hot springs, river sediments) from 40 worldwide locations, for the pre-screening stage are described in Table A1.

The 22 samples used for an in-depth study were the following ones: (i) five samples, collected in January 2017, were originating from the ombrotrophic Mougau peatland area (Brittany, France). They consisted of four peatland soil samples (MOUG1–MOUG4) collected in January 2017, in the peat surface layer (0–10 cm), and one sediment sample from a freshwater stream (MOUG5) collected in the upper sediment (5–10 cm); (ii) four samples were collected from the center of the deep-sea methane-emitting Håkon Mosby mud volcano, located at 1280 m water depth, in the Barents Sea (Arctic Ocean), during the 2009 ARK-XXIV/2 (PS74) oceanographic cruise. Sedimentary horizons selected after the pre-screening stage were the 0–1 cm (HM1), 1–6 cm (HM2) and 6–11 cm (HM3) sediments from the PS74/168 core, and the 1–6 cm (HM4) surface layer sediments from the PS74/189 core; (iii) three anoxic samples were collected in December 2018, in the water column of the meromictic crater Lake Pavin (Auvergne, France) at 60, 70 and 80 m water depth corresponding, respectively, to the mixolimnion-chemocline interface (PAV60), the chemocline-subchemocline interface (PAV70) and the subchemocline water (PAV80) [4]; (iv) one geothermal hot spring sediment sample was collected, in November 2013, from the Xiada reservoir of the Xiamen botanical garden (China) (XIA); (v) one geothermal spring sediment sample was taken from an ephemeral hot spring located in the tidal sway zone on the beach called Feu de Joie, at the Kerguelen islands (Kerguelen

archipelago, France, Indian Ocean) (KERG), in December 2016; (vi) two samples were originating from the athalassic ($MgCl_2$-rich) deep-sea hypersaline anoxic brine (DHAB) Kryos, in the Mediterranean Sea. They were respectively collected from layers at 150 g l^{-1} salts (mainly $MgCl_2$) (KRY150) and 238 g l^{-1} (mainly $MgCl_2$) (KRY238) [5], in May 2016; (vii) three sediments samples were collected in a pockmark field during the 2015 PAMELA-MOZ4 oceanographic survey in the Mozambique Channel (off the Madagascar island). Those samples consisted in the first 2–4, 4–6 and 6–11 cm layers of the CSF4 sediment cores referenced as MOZ1, MOZ2 and MOZ3, respectively; (viii) one surficial marine sediment sample (15–20 cm) was collected from the Dourduff-en-Mer marine bay (DOUR), in February 2017, on the coast of Brittany (English Channel, France); (ix) one deep-sea sediment sample was collected from the South West Indian Ocean (COMRA) during the April 2015 COMRA oceanographic cruise; (x) finally, a freshwater sediment sample was taken from a stream (0–10 cm) feeding the coastal river Penfeld in February 2017 (PENF) (Brittany, France). Given that the ultimate goal of this study was to grow *Methanomassiliicoccales*, all the investigated samples were stored at 4 °C.

Text S3. Total DNA extraction for metabarcoding screening

The standardized DNA extraction protocol which was used on the 22 samples selected for an in-depth characterization combined mechanical and chemical lysis. It was applied in triplicate to each sample (+ one negative control). In summary, each DNA extraction was performed onto 0.5 g of environmental matrix. A mechanical lysis of the sample was first carried out by bead-beating (5000 rpm, 15 s), using the Precellys 24® tissue homogenizer (Bertin instruments, France), in a FastDNA® Lysing Matrix tube E (FastDNA® SPIN kit for soil, MP Biomedicals) containing 1 ml of TE-Na-1× lysis buffer (100 mM Tris, 50 mM EDTA, 100 mM NaCl, pH 8.0) in addition to the sample. Then, the lysate supernatants were subjected to a DNA extraction protocol combining chemical and enzymatic lysis procedures, and a classical phenol/chloroform/isoamyl alcohol (PCI: 25/24/1; pH 8.0) extraction of nucleic acids, as described elsewhere [6]. Nucleic acids were then precipitated with 0.8 volumes of icecold isopropanol, in the presence of 400 µl Na-acetate (3M) and 5 µl linear acrylamide (Ambion, Applied Biosystems). Elution was performed in 40 µl EB buffer (Qiagen) before pooling. DNA extractions from enrichment cultures were performed as described above, except that the mechanical lysis stage has been omitted. For these extracts, elution was performed in 100 µl EB buffer.

Nucleic acid solution quality was determined using the NanoDrop™ 8000 (Thermo Scientific) spectrophotometer. Double-strand DNA concentration was measured using the kit Quantifluor™ dsDNA system (Promega), following the manufacturer's instructions.

Text S4. Details of the barcoding and the phylogenetic analyses

Section 4.1. FASTQ generation

Amplicons from bulk samples and enrichment cultures were generated by the company Molecular Research-MrDNA (Shallowater, Texas, USA), using the Illumina MiSeq (2 × 300 bp, paired-end reads) technology, with the kits and according to the manufacturer's instructions. FASTQ were generated using the "Combine FASTA and QUAL into FASTQ (Galaxy v. 1.0.1)" [7] tool on the FROGS pipeline. FASTA and Quality Score files were joined according to a mapping file and the ASCII force quality score to create a single FASTQ block for each read. The quality of raw sequence data were then checked using the "FastQC Read Quality reports" (v. 0.11.4) tool (http://www.bioinformatics.babraham.ac.uk/projects/fastqc/).

Section 4.2. OTU generation and comparison

Once data from enrichment cultures were generated by the company and in order to facilitate OTU comparison between prokaryotic diversity in bulk samples and in enrichment cultures, raw FASTQ files from both sequencing runs were merged into a unique file before processing as described

in 2.3. *Sequencing and sequence analysis* (main manuscript). This resulted on the generation of a unique BIOM file comprising representative 16S rRNA OTU abundance, and taxonomy into each samples.

Section 4.3. Consensus sequences workflow

Extraction of potential reagent contaminants and normalization of representative 16S rRNA gene OTUs were performed using the R studio program (v. 1.3.5001), the phyloseq (v. 1.28.0 R), the decontam (v. 1.4.0) and the metagenomSeq (v. 1.26.3) packages. As the metadata between the bulk samples and in the enrichment cultures were different, two distinct sequence treatments were performed from the previously generated single BIOM file. The BIOM files were converted to a .csv file before being implemented in the R program. Our scripts were based on workflows described elsewhere (https://bioconductor.org/help/course-materials/2017/BioC2017/Day1/Workshops/Microbiome/MicrobiomeWorkflowII.html and https://benjjneb.github.io/decontam/vignettes/decontam_intro.html, respectively). Workflow used to decontaminate and normalize 16S rRNA gene sequences representatives of the OTUs is listed below. Additional information about the script is preceded by a "#".

Step 1. Loading tables into R program

```
setwd("File_path")
OTUtable <- read.table("OTUs_file.csv",header = T, row.names = 1, sep = ";")
Metadata <- read.table("Bulk_Metadata_file.csv", header = T, row.names = 1, sep = ";")
Taxo <- read.table("Taxonomy_Table_file.csv", header = T, row.names = 1, sep = ";")
Taxo <- as.matrix(Taxo)
```

Step 2. Create a Phyloseq object

```
library(phyloseq)
OTU <- otu_table(OTUtable, taxa_are_rows = TRUE)
Meta <- sample_data(Metadata)
Tax <- tax_table(Taxo)
Phymatrice <- phyloseq(OTU, Meta, Tax)
Phymatrice
#Here, "Phymatrice" refers to the Phyloseq object.
```

Step 3. Remove unwanted taxa

Potential contaminant taxa such as *Chloroplast*, *Mitochondria*, *Eukaryota* or unclassified Kingdom level taxa are removed.

```
subset_taxa(Phymatrice, Order!="Chloroplast" )
Phy <- subset_taxa(Phymatrice, Order!="Chloroplast" )
Phy2 <- subset_taxa(Phy, Kingdom!="Unclassified" )
Phy3 <- subset_taxa(Phy2, Family!="Mitochondria" )
Phymatrice <- subset_taxa(Phy3, Kingdom!="Eukaryota" )
Phymatrice
```

Step 4. Sequence decontamination using the "Decontam" tool

```
# Library size inspection
library(ggplot2)
library(decontam)
df <- as.data.frame(sample_data(Phymatrice))
df$LibrarySize <- sample_sums(Phymatrice)
df <- df[order(df$LibrarySize),]
df$Index <- seq(nrow(df))
ggplot(data=df, aes(x=Index, y=LibrarySize, color=Sample_or_Control)) + geom_point()
```

Contaminant identification – Frequency approach, based on Picogreen technology DNA assay.
```
contamdf.freq <- isContaminant(Phymatrice, method="frequency", conc="quant_reading")
head(contamdf.freq)
table(contamdf.freq$contaminant)
head(which(contamdf.freq$contaminant))
plot_frequency(Phymatrice, taxa_names(Phymatrice)[c(1,3)], conc="quant_reading") + xlab("DNA Concentration (PicoGreen fluorescent intensity)")
set.seed(100)
plot_frequency(Phymatrice,taxa_names(Phymatrice)[sample(which(contamdf.freq$contaminant),3)], conc="quant_reading") + xlab("DNA Concentration (PicoGreen fluorescent intensity)")
Phymatrice.noncontam <- prune_taxa(!contamdf.freq$contaminant, Phymatrice)
Phymatrice.noncontam = filter_taxa(Phymatrice.noncontam, function(x) sum(x) > 0, TRUE)
Phymatrice <- Phymatrice.noncontam
```

<u>Step 5. Data normalisation using the metagenomSeq tool</u>
```
library(Biostrings)
library(ggplot2) + theme_set(theme_bw())
library("metagenomeSeq")
all_MR <- phyloseq_to_metagenomeSeq(Phymatrice)
all_MR_nor <- cumNorm(all_MR)
all_df_nor <- MRcounts(all_MR_nor, norm = T)
Phymatrice_norm <- phyloseq(otu_table(all_df_nor, taxa_are_rows = TRUE), tax_table(Phymatrice), sample_data(Phymatrice))
rm(all_MR, all_MR_nor)
Phymatrice_norm #Normalised phyloseq object
```

<u>Step 6. Export OTU, taxonomy and metadata tables</u>
```
#Exporting OTU, taxonomy and metadata tables to separate .csv files could be useful for further investigations.
library(microbiome)
TaxoMarcEnrich = write_phyloseq(Phymatrice_norm, "TAXONOMY")
TaxoMarcEnrich = write_phyloseq(Phymatrice_norm, "OTU")
TaxoMarcEnrich = write_phyloseq(Phymatrice_norm, "METADATA")
```

Section 4.4. Ecological Network analysis

Beta-diversity between bulks samples was assessed by Network analysis using a Bray-Curtis index, as ecological distance. First, the distance matrix was computed with a script Perl, then the percolation threshold was computed (see methods section) and the Gephi was used to visualize it.

Section 4.5. Phylogenetic analyses of Methanomassiliicoccales OTUs

All 16S rRNA gene sequences representative of *Methanomassiliicoccales* OTUs, generated from metabarcoding and metagenomic analyses, have been aligned and refined manually with other *Methanomassiliicoccales* sequences from both *Methanomassiliicoccales* clades published elsewhere [8–15] using MUSCLE (with 16 iterations maximum) and GBlocks (without contiguous non-conserved positions). Sequences affiliated to various methanogen lineages and sequences belonging to the clades RG8 and *Ca*. 'Thermoplasmatota' were also added to the dataset, and used as outgroup. The BIONJ method [16] was then applied on sequences with the modifications of Jukes and Cantor and using 1000 resampling bootstrap replicates to generate the final dendrogram.

Text S5. Media composition

Enrichment cultures targeting *Methanomassiliicoccales* were performed in a medium based on previously published recipes [8,17–19] and https://www.dsmz.de/microorganisms/medium/pdf/DSMZ_Medium141.pdf) that included a basal medium described in the body of the text, and a number of additives (fatty acids solution, hemin solution, selenite-tungstate solution, polyvitamin solution, K3 vitamin solution and coenzyme M solution) described in this section.

The fatty-acid solution contained, per litre: 25 g valeric acid, 25 g isovaleric acid, 25 g 2-methylbutanoic acid and 25 g isobutyric acid. Its pH was adjusted to 7.5 with concentrated NaOH. It was then filter-sterilized (pore-size, ∅ 0.2 μm), placed under a N_2 gas phase, and stored at 4 °C.

The porcine hemin solution was prepared as follows, for 100 ml: 50 mg porcine hemin (PanReac AppliChem, Barcelona, Spain) was dissolved into 1 ml NaOH before adjusting the volume to 100 ml with distilled water. The solution was then sterilized by filtration (pore-size, ∅ 0.2 μm) before to be stored at 4 °C, under a N_2 atmosphere.

The tungstate-selenite solution contained, per litre: 0.5 g NaOH, 3 mg $Na2SeO_3.5H_2O$, 4 mg $NaWO_4.2H_2O$. Solution was sterilized by autoclaving and stored under N_2 at 4 °C, until use.

The polyvitamin solution (from the DSMZ141 medium) contained, per litre: 2 mg biotin, 2 mg folic acid, 10 mg pyrodoxine-HCl, 5 mg thiamine-HCl.$2H_2O$, 5 mg riboflavin, 5 mg nicotinic acid, 5 mg D-Ca-pantothenate, 0.10 mg cobalamin (= vitamin B12), 5 mg *p*-aminobenzoic acid and 5 mg lipoic acid. It was filter-sterilized (pore-size, ∅ 0.2 μm), kept under a N_2 gas phase, and stored at 4 °C in the dark, preferentially in a bottle of brown glass.

The coenzyme-M solution contained, for 100 ml: 0.164 g Na-2-mercaptoethanesulfonate dissolved into 100 ml sterile water before filtering onto a ∅ 0.2 μm membrane. Headspace was filled with pure N_2 gas and stored at 4 °C, in a bottle of brown glass, until use.

The K3 vitamin solution was prepared as follows: 5 mg/ml vitamin K3 was dissolved in 95% (v/v) ethanol and then diluted 1‰th in distilled water (final concentration 0.05 mg/ml). The solution was then filter-sterilized and stored at 4 °C under N_2, in an opaque flask, until use.

Text S6. Quantification of substrates and metabolic products

Headspace CH_4, H_2, N_2 and CO_2 were measured using a modified INFICON/Micro GC FUSION Gas Analyzer (INFICON, Basel, Switzerland) fitted with a pressure gauge and two conductivity detectors. Separation was performed using two columns: a 10m molecular sieve column at 65 °C and Ar as a carrier gas; and a 12m RT-Q at 60 °C using He as a carrier gas. Gas concentrations were calculated using the method of Mah and colleagues [20] and the results corrected for the decrease in culture medium volume caused by the withdrawal of samples for analysis by ion chromatography.

Samples for cation, anion and methanol analyses were collected in 2 ml tubes and stored at –20 °C. The dilution of each sample was optimized prior to analysis. Sodium, methylated amines (methylamine, dimethylamine, trimethylamine, choline) and ammonium were analyzed using a Dionex ICS-900 Ion Chromatography System (Dionex, Camberley UK) coupled with a CERS 500 4 mm suppressor and a DS5 conductivity detector (40 °C) and fitted with a RFC-10 Reagent-Free Controller™, an ASDV autosampler, and an IonPac CS16 column maintained at 60 °C in a UltiMate™ 3000 Thermostated Column Compartment (Thermo Scientific, Waltham, MA, USA). The gradient program was as follows: 18 mM methanesulfonic acid (MSA) for 40 min, increase from 10.4 mM MSA min^{-1} to 70 mM (10 min), and then decrease from 52 mM MSA min^{-1} to 18 mM (9 min). Acetate, nitrate, thiosulfate and sulfate concentrations were quantified by anion chromatography as described elsewhere [21]. Analysis of methanol concentrations was carried out using an Agilent 6890N Gas

Chromatograph (GC) (Agilent, Santa Clara, USA) coupled with a Flame Ionization Detector (FID) instrument. 1 ml of a mixture composed of 400 µl of culture supernatant, 450 µl of ultrapure water and 150 µl of internal standard (Butanol-1) were placed into a 10 ml vial for heating desorption. Then, 500 µl of sample from the gaseous space were injected into the GC-FID using a CTC Combipal autosampler Headspace (Agilent, Santa Clara, USA). Compounds were separated using a Cp-Sil 8CB VARIAN column (30 m, 0.25 mm, *i.d*; 0.25 µm stationary film thickness) (Agilent, Santa Clara, USA) using the following program: 3 min at 40 °C, followed by an increase of 10 °C min^{-1} up to 70 °C, held for 15 min; injector and detector temperatures, 250 °C; Nitrogen (2.9 ml min^{-1}) was used as a carrier gas.

Supplementary Tables and Figures

Table S1. Origin and location of the 86 environmental samples screened as a first approach for the research of *Methanomassiliicoccales*.

Sample acronym	Sample formal name	Sample type	Geographical Origin	Sampling expedition	Latitude	Longitude	Sampling date	Other characteristics	References
**COMRA**	COMRA 34-11 (TB;OI)	Deep-sea sediments	SW Indian ocean	COMRA 2015	38°11'31.2S	50°42'57.6" E	12/14/2016	Marine sediments	
**DOURD**	DOURD	Coastal sediments	Morlaix bay (France)		48°37'47" N	03°50'47" W	01/28/2017	River-influenced marine sediments	
**HM1**	HM168 0-1	Volcano mud	Barents sea (Norway)	ARK-XXIV/2 (PS74)	72°0'N	14°44'E	07/23/2007	Methane emitting mud volcano	
**HM2**	HM168 1-6	Volcano mud	Barents sea (Norway)	ARK-XXIV/2 (PS74)	72°0'N	14°44'E	07/23/2007	Methane emitting mud volcano	
**HM3**	HM168 6-11	Volcano mud	Barents sea (Norway)	ARK-XXIV/2 (PS74)	72°0'N	14°44'E	07/23/2007	Methane emitting mud volcano	
**HM4**	HM189 1-6	Volcano mud	Barents sea (Norway)	ARK-XXIV/2 (PS74)	72°0'N	14°44'E	Sept. 2007	Methane emitting mud volcano	
**KERG**	Kerguelen - Rallier du Baty Source PFJ	Geothermal water	Kerguelen Island (French TAAF)	IPEV n° 1077, TALISKER program	49°38'40.4" S	68°47'02.1" E	12/14/2016	Hot spring located at the "Feu de Joie" beach	
**KRY150**	Kryos150	Deep-sea hypersaline anoxic brine	Kryos basin (Mediterranean sea)	-	35°02'N	22°01'E	05/17/2016	150 $g.l^{-1}$ salts ($MgCl_2$-rich)	
**KRY238**	Kryos238	Deep-sea hypersaline anoxic brine	Kryos basin (Mediterranean sea)	-	35°02'N	22°01'E	05/17/2016	238 $g.l^{-1}$ salts ($MgCl_2$-rich)	
**MOUG1**	TB3	Peatland soil	Commana peatland (France)	-	48°23'56" N	3°57'56" W	01/25/2017	Slightly acidic	
**MOUG2**	TB4	Peatland soil	Commana peatland (France)	-	48°23'53" N	3°57'56" W	01/25/2017	Acidic	
**MOUG3**	TB5	Peatland soil	Commana peatland (France)	-	48°23'48" N	3°57'51" W	01/25/2017	Acidic	
**MOUG4**	TB7	Peatland soil	Commana peatland (France)	-	48°23'53.16" N	3°57'55.8" W	02/29/2018	Acidic	
**MOUG5**	Riv1	Freshwater sediments	Commana peatland (France)	-	48°23'55" N	3°57'6'" W	01/25/2017	Stream running through a bog	
**MOZ1**	MOZ4 MTB03 2-4 cm culture anaérobie 11/15	Sediments from a pockmarck area	Mozambique Channel (Madagascar shore)	PAMELA-MOZ4	15°22'14" S	45°57'06" E	Nov. 2015	Area of cold fluid migration	
**MOZ2**	MOZ4 MTB03 4-6 cm culture anaérobie 11/15	Sediments from a pockmarck area	Mozambique Channel (Madagascar shore)	PAMELA-MOZ4	15°22'14" S	45°57'06" E	Nov. 2015	Area of cold fluid migration	
**MOZ3**	MOZ4 MTB03 6-11 cm culture anaérobie 11/15	Sediments from a pockmarck area	Mozambique Channel (Madagascar shore)	PAMELA-MOZ4	15°22'14" S	45°57'06" E	Nov. 2015	Area of cold fluid migration	
**PAV1**	PAVIN60	Anoxic water from a meromictic lake	Pavin lake (France)	-	45°29'45" N	2°53'18" E	12/17/2018	Ferrugineous and sulfidic waters	

Sample acronym	Sample formal name	Sample type	Geographical Origin	Sampling expedition	Latitude	Longitude	Sampling date	Other characteristics	References
**PAV2**	PAVIN70	Anoxic water from a meromictic lake	Pavin lake (France)	-	45°29'45" N	2°53'18" E	12/17/2018	Ferrugineous and sulfidic waters	
**PAV3**	PAVIN80	Anoxic water from a meromictic lake	Pavin lake (France)	-	45°29'45" N	2°53'18" E	12/17/2018	Ferrugineous and sulfidic waters	
**PENF**	Sed2 Penfeld	Freshwater sediments	Brest Kervallon garden (France)	-	48°23'38.82" N	4°30'36.12" W	02/06/2017	Coarse sand	
**XIA**	XIA	Water from a geothermal hot spring	Xiamen Botanical Garden (China)	-	24°26'25" N	118°6'17" E	12/09/2013	Iron-rich hot spring with a salinity of 1.3%	
	Grand-Dellec	Coastal sediments	Brest bay (France)	-	48°21'5" N	4°34'12" W	Jan. 2017	Marine sediments	
	Sainte-Anne	Coastal sediments	Brest bay (France)	-	48°21'41" N	4°33'12" W	Jan. 2017	Marine sediments	
	COMRA 34-9 (TB;OI)	Deep-sea sediments	South West Indian ocean	COMRA 2015				Marine sediments	
	COMRA 34-10 (TB;OI)	Deep-sea sediments	South West Indian ocean	COMRA 2015	38°11'32" S	50°42'56" E	Avr. 2015	Marine sediments	
	COMRA 34-12 (TB;OI)	Deep-sea sediments	South West Indian ocean	COMRA 2015	38°11'32.2" S	50°42'56.4" E	Avr. 2015	Marine sediments	
	COMRA 34-28 (TB;OI)	Deep-sea sediments	South West Indian ocean	COMRA 2015	38°10'62.65" S	49°59'42.28" E	Mai 2015	Marine sediments	
	BIG, 4, CT4, S1 0-5 cm	Deep-sea hydrothermal vent	Guaymas basin (Mexico)	BIG	27°25.484' N	111° 30.074' W	06/14/2010		https://campagnes.flotteoceanographique.fr/prl?id=BFBGX-43350
	BIG, 4, CT4, S1 15-20 cm	Deep-sea hydrothermal vent	Guaymas basin (Mexico)	BIG	27°25.484' N	111° 30.074' W	06/14/2010		https://campagnes.flotteoceanographique.fr/prl?id=BFBGX-43350
	YK13-05 MCR 6K 1350 BEEBE WOOD Ifremer	Deep-sea hydrothermal vent	Mid-Cayman Rise	YK13-05	18°32'76.41" N	81°43'08.86" W	06/22/2013		
	YK13-05 MCR 6K 1356 Hole to Hell	Deep-sea hydrothermal vent	Mid-Cayman Rise	YK13-05	18°22'58" N	81°47'89" W	06/28/2013		
	YK13-05 MCR 6K 1354 BVF #Ifremer	Deep-sea hydrothermal vent	Mid-Cayman Rise	YK13-05	18°32'78.77" N	81°43'09.55" W	06/26/2013		
	YK13-056K FH1349A BEEBE# Left	Deep-sea hydrothermal vent	Mid-Cayman Rise	YK13-05	18°32'47" N	81°43'7.1" W	06/21/2013		
	Urania	Deep-sea mud volcano	Mediterranean Sea		35°60'N	22°01'E	05/18/2016		
	KESC 9 - 14 50 cm	Deep-sea sediments	Var ridge, Mediterranean Sea (France)	RHOSOS	43°32'26" N	7°11'13.999" E	10/07/2008		
	KESC 9 - 14 180 cm	Deep-sea sediments	Var ridge, Mediterranean Sea (France)	RHOSOS	43°32'26" N	7°11'13.999" E	10/07/2008		
	KESC 9 - 14 140 10/08	Deep-sea sediments	Var ridge, Mediterranean Sea (France)	RHOSOS	43°23′.016 N	07°44′.187 E	10/07/2008		[22]
	KESC 9 - 14 180 10/08	Deep-sea sediments	Var ridge, Mediterranean Sea (France)	RHOSOS	43°23′.016 N	07°44′.187 E	10/07/2008		[22]
	KESC 9 - 14 200 10/08	Deep-sea sediments	Var ridge, Mediterranean Sea (France))	RHOSOS	43°23′.016 N	07°44′.187 E	10/07/2008		[22]

Sample acronym	Sample formal name	Sample type	Geographical Origin	Sampling expedition	Latitude	Longitude	Sampling date	Other characteristics	References
	KESC 9 - 14 240 10/08	Deep-sea sediments	Var ridge (France)	RHOSOS	43°23′.016 N	07°44′.187 E	10/07/2008		[22]
	KESC 9 - 14 280 10/08	Deep-sea sediments	Var ridge (France)	RHOSOS	43°23′.016 N	07°44′.187 E	10/07/2008		[22]
	Leg 317 3H1	Deep-sea sediments	Canterbury ridge (New-Zealand)		44°56'26.62" S	172°1'36" E	Nov. 2009		
	RHS-KS-33-50 10/08	Deep-sea sediments	Gulf of Lion, Western Mediterranean Sea (France)	RHOSOS	42°41′.596 N	03°50′.493 E	Oct. 2008		[22]; https://campagnes.flotteoceanographique.fr/prl?id=BFBGX-86654
	RHS-KS-33-150 10/08	Deep-sea sediments	Gulf of Lion, Western Mediterranean Sea (France)	RHOSOS	42°41′.596 N	03°50′.493 E	Oct. 2008		[22]; https://campagnes.flotteoceanographique.fr/prl?id=BFBGX-86654
	RHS-KS-33-350 10/08	Deep-sea sediments	Gulf of Lion, Western Mediterranean Sea (France)	RHOSOS	42°41′.596 N	03°50′.493 E	Oct. 2008		[22]; https://campagnes.flotteoceanographique.fr/prl?id=BFBGX-86654
	RHS-KS-33-450 10/08	Deep-sea sediments	Gulf of Lion, Western Mediterranean Sea (France)	RHOSOS	42°41′.596 N	03°50′.493 E	Oct. 2008		[22]; https://campagnes.flotteoceanographique.fr/prl?id=BFBGX-86654
	RHS-KS-33-550 10/08	Deep-sea sediments	Gulf of Lion, Western Mediterranean Sea (France)	RHOSOS	42°41′.596 N	03°50′.493 E	Oct. 2008		[22]; https://campagnes.flotteoceanographique.fr/prl?id=BFBGX-86654
	RHS-KS-33-710 10/08	Deep-sea sediments	Gulf of Lion, Western Mediterranean Sea (France)	RHOSOS	42°41′.596 N	03°50′.493 E	Oct. 2008		[22]; https://campagnes.flotteoceanographique.fr/prl?id=BFBGX-86654
	TVG10 - 20V - S20 Jaigo 3.12	Deep-sea sediments	North West Indian ocean	-					
	Sed3 Penfeld	Freshwater sediments	Brest Kervallon garden (France)	-	48°23'39.5" N	4°30'35.9" W	02/06/2017		
	Kerguelen - Rallier du Baty Source 15	Water from a geothermal hot spring	Kerguelen Island (French TAAF)	IPEV n° 1077, TALISKER program	49°36'S	68°56'E	12/13/2016		
	Kerguelen - Rallier du Baty Source 18	Water from a geothermal hot spring	Kerguelen Island (French TAAF)	IPEV n° 1077, TALISKER program	49°36'S	68°56'E	12/13/2016		
	Kerguelen - Rallier du Baty Source 107	Water from a geothermal hot spring	Kerguelen Island (French TAAF)	IPEV n° 1077, TALISKER program	49°36'S	68°56'E	12/14/2016		
	XIA2	Water from a geothermal hot spring	Xiamen Botanical Garden (China)	-	24°26'25" N	118°6'17" E	12/09/2013		
	PAVIN60-2	Anoxic water from a meromictic lake	Pavin lake (France)	-	45°29'45" N	2°53'18" E	10/24/2016	Ferrugineous and sulfidic waters	

Sample acronym	Sample formal name	Sample type	Geographical Origin	Sampling expedition	Latitude	Longitude	Sampling date	Other characteristics	References
	PAVIN70-2	Anoxic water from a meromictic lake	Pavin lake (France)	-	45°29'45" N	2°53'18" E	10/24/2016	Ferrugineous and sulfidic waters	
	Aydat	Anoxic water from a meromictic lake	Aydat lake (France)	-	45°39'50" N	02°59'12" E	10/24/2016		
	TREM1	Peatland soil	Trémaouézan peatland(France)	-	48°30'19" N	04°16'21" W	01/25/2017	Slightly acidic	
	TREM2	Peatland soil	Trémaouézan peatland (France)	-	48°30'27" N	04°16'23" W	01/25/2017	Slightly acidic	
	TB6	Peatland soil	Commana peatland (France)	-	48°23'56.8" N	3°57'56.3" E	01/25/2017	Slightly acidic	
	TB8	Peatland soil	Commana peatland (France)	-	48°23'53.16" N	3°57'55.8" W	02/26/2018	Slightly acidic	
	TB9	Peatland soil	Commana peatland (France)	-	48°23'53.88" N	3°57'55.8" W	02/26/2018	Slightly acidic	
	TB10	Peatland soil	Commana peatland (France)	-	48°23'56.76" N	3°57'56.52" W	02/26/2018		
	131.18.1.S2	Sediments from a pockmark area		-	04°45'605" S	9°56'423" E			[23]
	GeoB 13114-2-S1	Sediments from a pockmark area	Hydrate Hole (Guinea)	M76-/3a	04°48'57" S	09°54'50" E	06/26/2008		[23]
	GeoB 13114-2-S2	Sediments from a pockmark area	Hydrate Hole (Guinea)	M76/3a	04°48.581 S	09°54.496 E	06/26/2008		[23]
	GeoB 13114-2-S4	Sediments from a pockmark area	Hydrate Hole (Guinea)	M76/3a	04°48.581 S	09°54.496 E	06/26/2008		[23]
	GeoB 13118-1-S1	Sediments from a pockmark area	Worm Hole (Guinea)	M76/3a	04°45'605" S	09°56'423" E	06/29/2008		[23]
	GeoB 13118-1-S2	Sediments from a pockmark area	Worm Hole (Guinea)	M76/3a	04°45'605" S	09°56'423" E	06/29/2008		[23]
	GeoB 13121-2-S3	Sediments from a pockmark area	REGAB (Guinea)	M76/3a	05°47.881 S	09°42.805 E	07/06/2008		[23]
	GeoB 13121-1-S4	Sediments from a pockmark area	REGAB (Guinea)	M76/3a	05°47.881 S	09°42.805 E	07/06/2008		[23]
	GeoB 14-2-S1	Sediments from a pockmark area		M76/3a					
	GeoB 14-2-S2	Sediments from a pockmark area		M76/3a					
	GeoB 14-2-S4	Sediments from a pockmark area		M76/3a					
	MOZ4 MTB03 11-16 cm culture anaérobie 11/15	Sediments from a pockmark area	Mozambique Channel (Madagascar shore)	PAMELA-MOZ4	15°22'14" S	45°57'06" E	Nov. 2015	Area of cold fluid migration	
	MOZ4 MTB03 16-21 cm culture anaérobie 11/15	Sediments from a pockmark area	Mozambique Channel (Madagascar shore)	PAMELA-MOZ4	15°22'14" S	45°57'06" E	Nov. 2015	Area of cold fluid migration	
	MOZ4 MTB03 21-24 cm culture anaérobie 11/15	Sediments from a pockmark area	Mozambique Channel (Madagascar shore)	PAMELA-MOZ4	15°22'14" S	45°57'06" E	Nov. 2015	Area of cold fluid migration	
	MOZ4 CZSF04 S1-25 culture anaérobie 11/2015	Sediments from a pockmark area	Mozambique Channel (Madagascar shore)	PAMELA-MOZ4			Nov. 2015	Area of cold fluid migration	

Sample acronym	Sample formal name	Sample type	Geographical Origin	Sampling expedition	Latitude	Longitude	Sampling date	Other characteristics	References
	MOZ4 CZSF04 S2-38 culture anaérobie 11/2015	Sediments from a pockmark area	Mozambique Channel (Madagascar shore)	PAMELA-MOZ4			Nov. 2015	Area of cold fluid migration	
	MOZ4 CZSF04 S3-25 culture anaérobie 11/2015	Sediments from a pockmark area	Mozambique Channel (Madagascar shore)	PAMELA-MOZ4			Nov. 2015	Area of cold fluid migration	
	MOZ4 CZSF04 S5-25 culture anaérobie 11/2015	Sediments from a pockmark area	Mozambique Channel (Madagascar shore)	PAMELA-MOZ4				Area of cold fluid migration	
	PAMELA MOZ01 MTB7 7-12	Sediments from a pockmark area	Mozambique Channel (Madagascar shore)	PAMELA-MOZ1			Nov. 2015	Area of cold fluid migration	
	PAMELA MOZ01 KGS03	Sediments from a pockmark area	Mozambique Channel (Madagascar shore)	PAMELA-MOZ1			Nov. 2015	Area of cold fluid migration	
	MD-PL-20-TI1 - 341-20-Ti1 10ml	Submarine brine	Chefren mud volcano, Nile Deep Sea fan, Eastern Mediterranean Sea (Egypt shore)	MEDECO	32° 6.584' N	28° 10.655' E	11/17/2007		
	MD-PL-20-MBS1 - 34-20-MBS-1	Submarine brine	Chefren mud volcano, Nile Deep Sea fan, Eastern Mediterranean Sea (Egypt shore)	MEDECO	31° 59.498' N	28° 3.512' E	11/17/2007		

Table S2. Number of 16S rRNA gene sequence reads of the various *Methanomassiliiccoccales*'OTUs, other methanogens OTUs and total prokaryotic OTUs generated by metabarcoding, in the various studied environmental samples and in culture-based experiments PENF, DOUR and MOUG2. Other OTUs: sum of the reads in the scarce OTUs of *Methanomassiliicoccales*.

Origin	Sample	*Methanomassiliicoccales*								Other methanogens	Total Prokaryotes
		OTU21	OTU101	OTU499	OTU30	OTU926	OTU1789	OTU2685	Other *Methanomassiliicoccales* OTUs		
Environmental samples	COMRA	0	0	0	10	0	0	0	0	16	33543
	DOUR	0	0	0	26	1	0	2	1	70	27577
	HM1	0	0	0	19	0	0	0	0	1153	83723
	HM2	0	0	0	22	0	0	0	1	1152	115163
	HM3	0	0	4	27	0	0	0	0	2795	135293
	HM4	0	0	0	13	0	0	0	0	312	76722
	KERG	1	98	1	64	0	0	0	0	205	112160
	KRY150	0	0	0	4	0	0	0	0	199	33073
	KRY238	0	0	0	9	0	0	0	0	17	32803
	MOUG1	0	0	1	11	0	0	0	0	32	21147
	MOUG2	0	0	1	20	3	0	0	0	1323	42023
	MOUG3	4	0	1	18	3	0	0	0	425	19965
	MOUG4	0	0	0	24	1	0	0	0	2519	51257
	MOUG5	0	0	0	7	0	0	0	0	65	43768
	MOZ1	3	3	23	131	4	0	2	5	1042	51432
	MOZ2	2	3	396	141	12	0	39	3	952	82240
	MOZ3	1	3	590	187	6	0	27	6	1147	77662
	PAV60	1	0	3	26	0	0	0	0	45	133308
	PAV70	0	9	1	50	22	0	0	0	328	151718
	PAV80	0	0	4	46	62	0	0	0	425	128262
	PENF	34	5	2	72	115	0	0	1	324	42386
	XIA	0	0	0	4	0	0	0	0	15	60330
Enrichment culture	PF T1	13	1	0	2	31	0	0	0	47	113032
	PF T2	57	3	0	0	1	0	0	0	61	128451
	PF T3	74	12	0	1	0	0	0	0	87	158370
	PF T4	28	5	0	0	10	0	0	0	43	237339
	PF T5	603	21	0	0	2	0	0	1	627	255024
	PF T6	5039	262	0	3	5	0	0	10	5319	339383
	PF T7	10365	1254	0	1	5	0	0	15	11640	188034
	PF T8	41550	6435	0	17	2	1	0	56	48061	321670

	**PF T9**	24161	3654	0	0	4	0	0	35	27854	319970
	**PF T10**	30291	3070	0	2	0	1	0	27	33391	384057
	**DF T1**	7	0	0	0	0	0	0	0	7	115420
	**DF T3**	25	0	0	0	0	3	0	0	28	248964
	**DF T6**	17	0	14	0	0	19	0	0	50	178985
	**DF T8**	20	6	0	0	0	28	0	0	54	212050
	**DF T10**	14	64	0	1	0	56	0	0	135	202382
	**TB T1**	10	0	0	0	17	0	0	0	27	153856
	**TB T4**	26	1	0	0	41	0	0	0	68	350070
	**TB T6**	18	1	0	0	10	0	0	0	29	203877
	**TB T8**	16	0	0	0	6	0	0	0	22	205008
	**TB T11**	20	1	0	3	8	1	0	0	33	284002

Table S3. List of the *Methanomassiliicoccales* 16S rRNA gene sequences identified in the metagenome of the culture-based experiment PENF T8 (8th week of incubation), and 16S rRNA gene representative sequences of OTUs identified as *Methanomassiliicoccales* by the SILVA 138 database in in the metabarcoding analysis.

>k119-35148<br>GCCACCGCTATTGGAATACGATTAAGACATGCGAGTCGAGAGACGTAAGGTCTCGGCGGACCGCTCAGTAACACGTGGATAACATGCCCTAAGGTGGG<br>GGATAATCTCGGGAAACTGAGGATAATACCCCATAGGTCTGATCTGCTGGAATGCTTTCAGGCCGAAAACTCCGGTGCCTTAGGATTGGTCTGCGGTCTA<br>TCAGGCTGTAGTGGGTGTAACGGACCCACTAGCCCGTGACGGATATGGGCCTTGAGAGAGGGAGCCCAGAGATGGACTCTGAGACATGAGTCCAGGCC<br>CTACGGGGCGCAGCAGTCGCGAAAACTTCGCAATGGGGGCAACCCCGACGAGGGAATTCCAAGTGCCAGCACTTTGTGTTGGCTGTTCTCCTGTCTAAA<br>AAACAGGAGGAGTAAGGGGCGGGTAAGACGGGTGCCAGCCGCCGCGGTAATACCCGCGCCCCTAGTGGTGGTCGATATTATTGAGCCTAAAACGTCCG<br>TAGCCGGTCTTCTAAATCCTTGGGTAAATCGGGCAGCTTAACTGTCCGAAGTCCGGGGAGACTGGGAGACTTGGGACCGGGAGAGGTCAGAGGTACTTC<br>TGGGGTAGGGGTAAAATCCTGTAATCCTGGAAGGACCACCGGTGGCGAAGGCGTCTGACTAGAACGGATCCGACGGTGAGGGACGAAGCCCTGGGGCG<br>CAAACGGGATTAGATACCCCGGTAGTCCAGGGTGTAAACGCTGCGGGCTTGGTGTTGGGGGTCCTTAGTGGGCGCCCAGTGCCGGAGAGAAGTTGTTAA<br>GCCTGCTGCTTGGGGAGTATGTCCGCAAGGATGAAACTTAAAGGAATTGGCGGGGGAGCACCGCAACGGGAGGAGCGTGCGGTTCAATTGGATTCAAC<br>GCCGAAAAACTCACCAGGAGCGACGGATATATGAAGGTCAAGCTGATGACTTTACCTGATTTTCCGAGAGGTGGTGCATGGCCGTCGTCAGTTCGTACC<br>GTAAGGCGTTCTCTTAAGTGAGATAACGAACGAGACCCTCACCATTAATTGCTAGTCCCTTCTCCGGAGGGGGCGCACATTAATGGACCGCTGGCGCTA<br>AGTCAGAGGAAGGAGAGGTCAACGGTAGGTCCGTATGCCCCGAATCCCCTGGGCAACACGCGCGCTACAAAGGGTGGGACAATGGGCTTCGACACCGA<br>AAGGTGAAGACAATCCTGAAACCCATCCATAGTTCGGATTGGGGGCTGTAACCCGCCCCCATGAAGCTGGATTCCGTAGTAATCGCTTGTCAACATCAA<br>GCGGTGAATATGCCCCTGCTCCTTGCACACACCGCCCGTCAAACCACCCGAGTTGGGTTTCAGTAAGGATGTCTCTAATTGGGGCCTTCGAACTGAGATT<br>CAGCAAGGAGGGTTAAGTCGTAACAAGGTATCTGTAGGGGAACCTGCAGATGGATCACCTCCTAC
>k119-91804<br>GCCACCGCTATTGGAATTCGATTAAGACATGCGAGTCGAGAGTCGCAAGGACTCGGCGGACCGCTCAGTAACACGCGGATAACATGCCCTTGGGTGGG<br>GGATAACCTCGGGAAACTGAGAATAATACCCCATAGATCTAGAAAGCTGGAATGCTTTTAGATCCAAAACTCCGGTGCCCAAGGATTGGTCTGCGGCGT<br>ATCAGGTTGTAGTGGGTGTAACGGACCCCCTAGCCTATGACGCGTAAGGGCCTTGAGAGAGGGAGCCCTGAGATGGACTCTGAGACATGAGTCCAGGCC<br>CTACGGGGCGCAGCAGTCGCGAAAACTTCACAATGGGGGAAACCCCGATGAGGGAATTCCAAGTGCCAGCACATTGTGTTGGCTGTTCTTTGGTCTAAA<br>AAACCAGAGAAGTAAGGGCCGGGTAAGACGGGTGCCAGCCGCCGCGGTAATACCCGCGGCCCGAGTGGTGGTCGATATTATTGAGCCTAAAACGTCCG<br>TAGCCGGTTTTGTAAATCCTTGGGTAAATCGACCAGCTTAACTGTTCGAAGTCCGGGGAGACTGCAAGACTTGGGATCGGGAGAGGTGAGAGGTACTTCT<br>GGGGTAGGGGTAAAATCCTGTAATCCTAGAAGGACCACCGGTGGCGAAGGCGTCTCACTAGAACGAATCCGACGGTGAGGGACGAAGCCCTGGGGCGC<br>AAACGGGATTAGATACCCCGGTAGTCCAGGGTGTAAACGCTGCGGGCTTGGTGTTGGGGGTCCTTTGAGGGCGCCCAGTGCCGGAGAGAAGTTGTTAAG<br>CCTGCTGCTTGGGGAGTACGTCCGCAAGGATGAAACTTAAAGGAATTGGCGGGGGAGCACCGCAACGGGAGGAGCGTGCGGTTCAATTGGATTCAACA<br>CCGGACAACTCACCAGGAGCGACGGTTACATGAAGGCCAGGCTGATGACCTTGCCTGATTTTCCGAGAGGTGGTGCATGGCCGTCGTCAGTTCGTACCG<br>TAAGGCGTTCTCTTAAGTGAGATAACGAACGAGACCCTCACCGCTAATTGCTAGTGTTCTCTCCGGAGAGCACGCACATTAACGGGACCGCTGTCGCTAA<br>GACAGAGGAAGGAGAGGTCAACGGTAGGTCCGTATGCCCCGAATCTCCTGGGCTACACGCGCGCTACAAAGGTTGGGACAATGGGCTCCGACATCGAA<br>AGGTGAAGGCAATCTCGAAACCCAATCGTAGTTCGGATTGAGGGTTGTAACTCACCCTCATGAAGCTGGATTCCGTAGTAATCGCGGATCAACAATCCG<br>CGGTGAATATGCCCCTGCTCCTTGCACACACCGCCCGTCAAACCACCCGAGTTGGGTTTCAGTAAGGATATCTCATTTTGGGGTCTTCGAACTGAGATTCA<br>GCAAGGAGGGTT
>OTU21<br>TACCCGCGCCCCTAGTGGTGGTCGATATTATTGAGCCTAAAACGTCCGTAGCCGGTCTTCTAAATCCTTGGGTAAATCGGGCAGCTTAACTGTCCGAAGT<br>CCGGGGAGACTGGAAGACTTGGGACCGGGAGAGGTCAGAGGTACTTCTGGGGTAGGGGTAAAATCCTGTAATCCTGGAAGGACCACCGGTGGCGAAGG<br>CGTCTGACTAGAACGGATCCGACGGTGAGGGACGAAGCCCTGGGGCGCAAACGGG
>OTU30<br>TACCCGCGCCCCAAGTGGTGGTCGATATTATTGAGCCTAAAACGTCCGTAGCCGGTCTTCTAAATCCTTGGGTAAATCGGGCAGCTCAACTGTCCGAATT<br>CCGGGGAGACTGGGAGACTTGGGACCGGGAGAGGTCAGAGGTACTTCTGGGGTAGGGGTAAAATCCTGTAATCCTGGAAGGACCACCGGTGGCGAAGG<br>CGTCTGACTAGAACGGATCCGACGGTGAGGGACGAAGCCCTGGGGCGCAAACGGG
>OTU101<br>TACCCGCGGCCCGAGTGGTGGTCGATATTATTGAGCCTAAAACGTCCGTAGCCGGTTTTGTAAATCCTTGGGTAAATCGACCAGCTTAACTGTTCGAAGT<br>CCGGGGAGACTGCAAGACTTGGGATCGGGAGAGGTGAGAGGTACTTCTGGGGTAGGGGTAAAATCCTGTAATCCTAGAAGGACCACCGGTGGCGAAGG<br>CGTCTCACTAGAACGAATCCGACGGTGAGGGACGAAGCCCTGGGGCGCAAACGGG
>OTU499<br>TACCCGCGGCCCGAGTGGTGCTCATTATTATTGAGCTTAAAGCGTTCGTAGCCGGCCCTGTAAATCCTTGGGTAAATCGGACAGCTCAACTGTTCGAATTC<br>CGAGGAGACTGCAGGGCTCGGGACCGGGAGAGGTTCGAGGTACTCTCGGGGTAGGGGTAAAATCCTGTAATCCTGAGAGGACCACCAGTGGCGAAGGC<br>GTCGAACTAGAACGGATCCGACGGTGAGGGACGAAGCCCTGGGGCGCAAACGGG
>OTU926<br>TACCCGCGGCCCGAGTGGTGGTCGATATTATTGAGCCTAAAACGTTCGTAGCCGGTCTTGTAAATCCTTGGGTAAATCGGCCAGCTTAACTGTCCGAAGT<br>CCGGGGAGACTGCAAGACTTGGGATCGGGAGAGGTCAGAGGTACTTCTGGGGTAGGGGTAAAATCCTGTAATCCTAGAAGGACCACCGGTGGCGAAGG<br>CGTCTGACTAGAACGAATTCGACGGTGAGGAACGAAGCCCTGGGGCGCAAACGGG
>OTU1789<br>TACCCGCAGCTCGAGTGGTGGTCGATATTATTGAGCCTAAAACGTCCGTAGCCGGATTCATAAATCCTTGGGTAAATCGGCTAGCTTAACTAACCGAATT<br>CCGAGGAGACTGTGGATCTTGGGACCGGGAGAGGTCAGAGGTACTTCTGGGGTAGGGGTAAAATCCTGTAATCCTAGAAGGACCACCGGTGGCGAAGG<br>CGTCTGACTAGAACGGATCCGACGGTGAGGGACGAAGCCCTGGGGCGCAAACGGG
>OTU2685<br>TACCCGCGGCCCGAGTGGTGTTCATTATTATTGAGCTTAAAGCGTTCGTAGCCGGCCTTGTAAATCCTTGGGTAAATCGGGCAGCTCAACTGTTCGAATTC<br>CGAGGAGACTGCAAGGCTCGGGACCGGGAGAGGTTCGAGGTACTCTCGGGGTAGGGGTAAAATCCTGTAATCCTGAGAGGACCACCAGTGGCGAAGGC<br>GTCGAACTAGAACGGATCCGACGGTGAGGGACGAAGCCCTGGGGCGCAAACGGG
>OTU16699<br>TACCCGCGCCCCTAGTGGTGGTCGATATTATTGAGCCTAAAACGTCCGTAGCCGGTCTTCTAAATCCTTGGGTAAATCGGGCAGCTTAACTGTCCGAAGT<br>CCGGGGAGACTGGGAGACTTGGGACCGGGAGAGGTGAGAGGTACTCCAGGGGTAGGAGTGAAATCCTGTAATCCTGGAAGGACCACCGGTGGCGAAG<br>GCGTCTGACTAGAACGGATCCGACGGTGAGGGACGAAGCCCTGGGGCGCAAACGGG
>OTU16700<br>TACCCGCGGCCCGAGTGGTGGTCGATATTATTGAGCCTAAAACGTCCGTAGCCGGTTTTGTAAATCCTTGGGTAAATCGACCAGCTTAACTGTTCGAAGT<br>CCGGGGAGACTGCAAGACTTGGGATCGGGAGAGGTGAGAGGTACTTCTGGGGTAGGGGTAAAATCCTGTAATCCTAGAAGGACCACCGGTGGCGAAGG<br>CGTCTCACTAGAACGAATCCGACGCTGAGGCGCGAAAGCGTGGGGAGCAAACAGG

>OTU15634<br>CACCGGCAGCTCAAGTGGTGGTCGATATTATTGAGCCTAAAACGTCCGTAGCCGGTTTTGTAAATCCTTGGGTAAATCGACCAGCTTAACTGTTCGAAGT<br>CCGGGGAGACTGCAAGACTTGGGATCGGGAGAGGTGAGAGGTACTTCTGGGGTAGGGGTAAAATCCTGTAATCCTAGAAGGACCACCGGTGGCGAAGG<br>CGTCTCACTAGAACGAATCCGACGGTGAGGGACGAAGCCCTGGGGCGCAAACGGG
>OTU15741<br>TACCCGCAGCCCGAGTGGTGGTCATTTTTATTGAGCCTAAAGCGTCCGTAGCCGGACTAATAAATCCTTGGGTAAATCGAACGGCTTAACCGTTCGAATT<br>CCGGGGAGACTGTTAGTCTTGGAACCGGGAGAGGTCAGAGGTACTTCGGGGGTAGGGGTAATATCCTGTAATCCTCGAAGGACCACCAGTGGCGAAGG<br>CGTCTGACTTGAACGGATTCGACGGTGAGGGACGAAGCCCAGGGGCGCAAACGGG
>OTU17456<br>CACCGGCAGCTCAAGTGGTGGCCATTTTTATTGAGCCTAAAACGTCCGTAGCCGGTTTTGTAAATCCTTGGGTAAATCGACCAGCTTAACTGTTCGAAGTC<br>CGGGGAGACTGCAAGACTTGGGATCGGGAGAGGTGAGAGGTACTTCTGGGGTAGGGGTAAAATCCTGTAATCCTGGAAGGACCACCGGTGGCGAAGGC<br>GTCTGACTAGAACGGATCCGACGGTGAGGGACGAAGCCCTGGGGCGCAAACGGG
>OTU17598<br>TACCCGCAGCTCGAGTGGTGATCATTTTTACTGAGCTTAAAGCGTTCGTAGCCGGTCTAGTAAATCCTTGGGTAAATCGGGCAGCTTAACTGTTCGAATTC<br>CGAGGGGACTGCTGGACTTGGGACCGGAAGAGGTCAAAAGTACTCTTGGGGTAGGGGTAAAATCCTGTAATCCCACGAGGACTACCAGTGGCGAAGGC<br>GTTTGACTAGGACGGATCCAACGGTAAGGAACGAAACCCCGGGGCACAAACGGG
>OTU19010<br>TACCCGCGCCCCTAGTGGTGGTCGATATTATTGAGCCTAAAACGTCCGTAGCCGGTCTTCTAAATCCTTGGGTAAATCGGGCAGCTTAACTGTCCGAAGT<br>CCGGGGAGACTGGGAGACTTGGGACCGGGAGAGGTCAGAGGTACTTCTGGGGTAGGGGTAAAATCCTGTAATCCTGGAAGGACCACCGGTGGCGAAGG<br>CGGCTAACTGGAACGGATCCGACGGTGAGGGACGAAGCCCTGGGGCGCAAACGGG
>OTU21513<br>TACCCGCGCCCCTAGTGGTGGTCGATATTATTGAGCCTAAAACGTCCGTAGCCGGTCTTCTAAATCCTTGGGTAAATCGGGCAGCTTAACTGTCCGAAGT<br>CCGGGGAGACTGGAAGACTTGGGACCGGGAGAGGTCAGAGGTACTTCTGGGGTAGGGGTAAAATCCTGTAATCCTGGAAGGACCACCGGTGGCGAAGG<br>CGTCTGACTAGAACGGATCCGACGGTGAGGGACGAAGCCCTGGGGGAGCGAACCGG
>OTU23995<br>TACCCGCGCCCCTAGTGGTGGTCGATATTATTGAGCCTAAAACGTCCGTAGCCGGTCTTCTAAATCCTTGGGTAAATCGGGCAGCTTAACTGTCCGAAGT<br>CCGGGGAGACTGGAAGACTTGGGACCGGGAGAGGTCAGAGGTACTTCTGGGGTAGGGGTAAAATCCTGTAATCCTGGAAGGACCACCGGTGGCGAAGG<br>CGTCTGACTAGAACGGATCCGACGCTCATGCACGAAGGCGTGGGGATCAAACAGG
>OTU26269<br>TACCCGCAGCCCGAGTGGTGGTCACTTTTATTGAGCCTAAAGCGTCCGTAGCCGGACAGGTAAATCCTTGGGTAAATCGAACGGCTTAACCGTTCGAATT<br>CCGGGGAGACTGCCTGTCTTGAGACCGGGAGAGGTCAGAGGTACTTAGGGGGTAGGGGTAATATCCTGTAATCCTCAAAGGACCACCAGTGGCGAAGG<br>CGTCTGACTTGAACGGATTTGACGGTGAGGGACGAAGCCCAGGGGCGCAAACGGG
>OTU28842<br>TACCCGCGCCCCAAGTGGTGGTCGATATTATTGAGCCTAAAACGTCCGTAGCCGGTCTTCTAAATCCTTGGGTAAATCGGGCAGCTTAACTGTCCGAAGT<br>CCGGGGATACTGGCAATCTAGGGACCGGGAGAGGTGAGAGGTACTCCAGGGGTAGGAGTGAAATCCTGTAATCCTGGAAGGACCACCGGTGGCGAAG<br>GCGTCTGACTAGAACGGATCCGACGGTGAGGGACGAAGCCCTGGGGCGCAAACGGG
>OTU28843<br>TACCCGCGGCCCAAGTGGTGGTCGATATTATTGAGCCTAAAACGTCCGTAGCCGGTTTTGTAAATCCTTGGGTAAATCGACCAGCTTAACTGTTCGAAGT<br>CCGGGGAGACTGCAAGACTTGGGATCGGGAGAGGTGAGAGGTACTTCTGGGGTAGGGGTAAAATCCTGTAATCCTGGAAGGACCACCGGTGGCGAAGG<br>CGTCTGACTAGAACGGATCCGACGGTGAGTAACGAAAGCCAGGGGCGCGAACCGG
>OTU28844<br>TACCCGCGGCCCGAGTGGTGGTCGATATTATTGAGCCTAAAACGTCCGTAGCCGGTTTTGTAAATCCTTGGGTAAATCGACCAGCTTAACTGCTCGAAGT<br>CCGGGGAGACTGCAAGACTTGGGATCGGGAGAGGTGAGAGGTACTTCTGGGGTAGGGGTAAAATCCTGTAATCCTAGAAGGACCACCGGTGGCGAAGG<br>CGTCTCACTAGAACGAATCCGACGGTGAGGGACGAAAGCTGGGGGCACGAACCGG
>OTU28845<br>TACCCGCGGCCCGAGTGGTGGTCGATATTATTGAGCCTAAAACGTCCGTAGCCGGTTTTGTAAATCCTTGGGTAAATCGACCAGCTTAACTGTTCGAAGT<br>CCGGGGAGACTGGAAGACTTGGGACCGGGAGAGGTCAGAGGTACTTCTGGGGTAGGGGTAAAATCCTGTAATCCTGGAAGGACCACCGGTGGCGAAGG<br>CGGCTAACTGGAACGAACCTGACGGTGAGTAACGAAAGCCAGGGGCGCGAACCGG
>OTU30110<br>CACCGGCAGCTCAAGTGGTCGATATTATTGAGCCTAAAACGTCCGTAGCCGGTCTTCTAAATCCTTGGGTAAATCGGGCAGCTTAACTGTCCGAAGTCCG<br>GGGAGACTGGAAGACTTGGGACCGGGAGAGGTCAGAGGTACTTCTGGGGTAGGGGTAAAATCCTGTAATCCTGGAAGGACCACCGGTGGCGAAGGCGT<br>CTGACTAGAACGGATCCGACGGTGAGGGACGAAGCCCTGGGGCGCAAACGGG
>OTU30122<br>TACCCGCGCCCCTAGTGGTGGTCGATATTATTGAGCCTTAAACGTCCGTAGCCGGTCTTCTATCCTTGGGTAAATCGGGCAGCTTAACTGTCCGAAGTCCG<br>GGGAGACTGGGAGACTTGGGACCGGGAGAGGTCAGAGGTACTTCTGGGGTAGGGGTAAAATCCTGTAATCCTAGAAGGACCACCGGTGGCGAAGGCGT<br>CTGACTAGAACGAATTCGACGGTGAGGAACGAAGCCCTGGGGCGCAAACGGG
>OTU32864<br>TACCCGCAGCCCGAGTGGTGGTCACTTTTATTGAGCCTAAAGCGTTCGTAGCCGGATTGGTAAATCTTTGGGGAAATCGAGCGGCTCAACCGTTCGAATT<br>CCGAAGACACTGCCAATCTTGGGACCGGGAGAGGTCTGAGGTACTTCGGGGGTAGGGGTAATATCCTGTAATCCTCGAAAGACCACCAGTGGCGAAGG<br>CGTCAGACTTGAACGGATCCGACGGTGAGGGACGAAGCCCAGGGTCGCAAACGGG
>OTU32865<br>TACCCGCAGCTCGAGTGGTGGTCATTTTTATTGAGCTTAAAGCGTTCGTAGCCGGTCCAGTAAATCCTTGGGTAAATCGGGCAGCTTAACTGTTCGAATTC<br>CGAGGGGACTGCTGGACTTGGGACCGGAAGAGGTCAAAAGTACTCTTGGGGTAGGGGTAAAATCCTGTAATCCCACGAGGACTACCAGTGGCGAAGGC<br>GTTTGACTAGGACGGATTCGACGGTAAGGAACGAAACCCCGGGTCGCAAACGGG
>OTU32869<br>TACCCGCGCCCCAAGTGGTGGTCGATATTATTGAGCCTAAAACGTCCGTAGCCGGTCTTCTAAATCCTTGGGCAAATCGGGCAGCTTAACTGTCCGAAGT<br>CCGGGGAGACTGGGAGACTTGGGACCGGGAGAGGTCAGAGGTACTTCTGGGGTAGGGGTAAAATCCTGTAATCCTGGAAGGACCACCGGTAGCGAAGG<br>CGTCTGACTAGGACGGATCCGACGGTGAGGGACGAAGCCCTGGGGCGCAAACGGG
>OTU32875<br>TACCCGCGCCCCTAGTGGTGGTCAATATTATTGAGCCTAAAACGTCCGTAACCGGTCTTCTAAATCCTTGGGTAAATCGGGCAGCTTAACTGTCCGAAGT<br>CCGGGGAGACTGGGAGACTTGGGACCGGGAGAAGTCAGAGGTACTTCTGGGGTAGGGGTAAAATCCTGTAATCCTGGAAGGACCACCGGTGGCGAAGG<br>CGTCTGACTAGAACGGATCCGACGGTGAGGGACGAAGCCCTGGGGCGCAAACGGG
>OTU32876<br>TACCCGCGCCCCTAGTGGTGGTCGATATTATTGAGCCTAAAACGTCCGTAGCCGGATTCATAAATCCTTGGGTAAATCGGCTAGCTTAACTAACCGAATT<br>CCGAGGAGACTGTGGATCTTGGGACCGGGAGAGGTCAGAGGTACTTCTGGGGTAGGGGTAAAATCCTGTAATCCTAGAAGGACCACCGGTGGCGAAGG<br>CGTCTGACTAGAACGGATCCGACGGTGAGGGACGAAGCCCTGGGGCGCAAACGGG

>OTU32879<br>TACCCGCGCCCCTAGTGGTGGTCGATATTATTGAGCCTAAAACGTCCGTAGCCGGTCTTCTAAATCCTTGGGTAAATCGGGCAGCTTAACTGTTCGAAGTCCGGGGAGACTGCAAGACTTGGGATCGGGAGAGGTGAGAGGTACTTCTGGGGTAGGGGTAAAATCCTGTAATCCTAGAAGGACCACCGGTGGCGAAGGCGTCTCACTAGAACGAATCCGACGGTGAGGGACGAAAGCTGGGGGAGCAAACCGG
>OTU32880<br>TACCCGCGCCCCTAGTGGTGGTCGATATTATTGAGCCTAAAACGTCCGTAGCCGGTCTTCTAAATCCTTGGGTAAGTCGGGCAGCTTAACTGTCCGAAGTCCGGGGAGACTGGGAGACTTGGGACCGGGAGAGGTCAGAGGTACTTCTGGGGTAGGGGTAAAATCCTGTAATCCTGGAAGGGCCGCCGGTGGCGAAGGCGTCTGACTAGAACGGATCCGACGGTGAGGGACGAAGCCCTGGGGCGCAAACGGG
>OTU32881<br>TACCCGCGCCCCTAGTGGTGGTCGATATTATTGAGCCTAAAACGTCCGTAGCCGGTTTTGTAAATCCTTGGGTAAATCGACCAGCTTAACTGTTCGAAGTCCGGGGAGACTGCAAGACTTGGGATCGGGAGAGGTGAGAGGTACTTCTGGGGTAGGGGTAAAATCCTGTAATCCTAGAAGGACCACCGGTGGCGAAGGCGTCTCACTAGAACGAATCCGACGCTGAGGCGCGAAAGCCAGGGGAGCGAACGGG
>OTU32895<br>TACCCGCGGCCCGAGTGGTGGTCGATATTATTGAGCCTAAAACGTCCGTAGCCGGTTTTGTAAATCCTTGGGTAAATCGACCAGCTTAACTGTTCGAAGTCCGGGGAGACTGCAAGACTTGGGATCGGGAGAGGTGAGAGGTACTTCTGGGGTAGGGGTAAAATCCTGTAATCCTGGAAGGACCACCGGTGGCGAAGGCGTCTGACTAGAACGGATCCGACGGTGAGGCGCGAAAGCGTGGGGAGCAAACAGG
>OTU32903<br>TACCGGCGGCTCGAGTGGTGGCCACTATTACTGGGCTTAAAGCGTCCGTAGCCGGTTTTGTAAATCCTTGGGTAAATCGACCAGCTTAACTGTTCGAAGTCCGGGGAGACTGCAAGACTTGGGATCGGGAGAGGTGAGAGGTACTTCTGGGGTAGGGGTAAAATCCTGTAATCCTGGAAGGACCACCGGTGGCGAAGGCGTCTGACTAGAACGGATCCGACGGTGAGGGACGAAGCCCTGGGGCGCAAACGGG
>OTU33783<br>TACGGAGGATCCGAGCGGTGGTCGATATTATTGAGCCTAAAACGTCCGTAGCCGGTTTTGTAAATCCTTGGGTAAATCGACCAGCTTAACTGTTCGAAGTCCGGGGAGACTGCAAGACTTGGGATCGGGAGAGGTGAGAGGTACTTCTGGGGTAGGGGTAAAATCCTGTAATCCTAGAAGGACCACCGGTGGCGAAGGCGTCTCACTAGAACGAATCCGACGGTGAGGGACGAAGCCCTGGGGCGCAAACGGG
>OTU34173<br>TACGGAGGATCCGAGTGGTGGTCGATATTATTGAGCCTAAAACGTCCGTAGCCGGTTTTGTAAATCCTTGGGTAAATCGACCAGCTTAACTGTTCGAAGTCCGGGGAGACTGCAAGACTTGGGATCGGGAGAGGTGAGAGGTACTTCTGGGGTAGGGGTAAAATCCTGTAATCCTGGAAGGACCACCGGTGGCGAAGGCGTCTGACTAGAACGGATCCGACGGTGAGGGACGAAGCCCTGGGGCGCAAACGGG
>OTU40076<br>TTAGAAACCCGCGCCCCGAGTGGTGGTCGATATTATTGAGCCTAAAACGTCCGTAGCCGGTCTTCTAAATCCTTGGGTAAATCGGGCAGCTTAACTGTCCGAAGTCCGGGGAGACTGGGAGACTTGGGACCGGGAGAGGTCAGAGGTACTTCTGGGGTAGGGGTAAAATCCTGTAATCCTGGAAGGACCACCGGTGGCGAAGGCGTCTGACTAGAACGGATCCGACGGTGAGGGACGAAGCCCTGGGGCGCAAACGGG
>OTU40952<br>AACCAGCTCTTCGAGTGGTGTCCATGATTATTGAGCTTAAAGCGTTCGTAGCCGGCCCTGTAAATCCTTGGGTAAATCGGACAGCTCAACTGTTCGAATTCCGAGGAGACTGCAGGGCTCGGGACCGGGAGAGGTTCGAGGTACTCTCGGGGTAGGGGTAAAATCCTGTAATCCTGAGAGGACCACCAGTGGCGAAGGCGTCGAACTAGAACGGATCCGACGGTGAGGGACGAAGCCCTGGGGCGCAAACGGG
>OTU41089<br>CACCGGCAACTCAAGTGGTGGCCATTTTTATTGAGCCTAAAACGTCCGTAGCCGGTCTTCTAAATCCTTGGGTAAATCGACCAGCTTAACTGTTCGAAGTCCGGGGAGACTGCAAGACTTGGGATCGGGAGAGGTGAGAGGTACTTCTGGGGTAGGGGTAAAATCCTGTAATCCTAGAAGGACCACCGGTGGCGAAGGCGTCTCACTAGAACGAATCCGACGGTGAGGGACGAAGCCCTGGGGCGCAAACGGG
>OTU42355<br>TACCCGCGCCCCGAGTGGTGGTCGATATTATTGAGCCTAAAACGTCCGTAGCCGGTCTTCTAAATCCTTGGGAAATCTGTCGGCTTAACCGACAGGCGTTTAAGGGATACTGGCAATCTAGGGACCGGGAGAGGTGAGAGGTACTTCTGGGGTAGGGGTAAAATCCTGTAATCCTGGAAGGACCACCGGTGGCGAAGGCGTCTGACTAGAACGGATCCGACGGTGAGGGACGAAGCCCTGGGGCGCAAACGGG
>OTU42356<br>TACCCGCGCCCCGAGTGGTGGTCGATATTATTGAGCCTAAAACGTCCGTAGCCGGTCTTCTAAATCCTTGGGTAAATCCTACAGCTTAACTGTTCGAAGTCCGGGGAGACTGCAAGACTTGGGATCGGGAGAGGTGAGAGGTACTTCTGGGGTAGGGGTAAAATCCTGTAATCCTAGAAGGACCACCGGTGGCGAAGGCGTCTCACTAGAACGAATCCGACGGTGAGGGACGAAGCCCTGGGGCGCAAACGGG
>OTU42357<br>TACCCGCGCCCCGAGTGGTGGTCGATATTATTGAGCCTAAAACGTCCGTAGCCGGTCTTCTAAATCCTTGGGTAAATCGACCAGCTTAACTGTTCGAAGTCCGGGGAGACTGCAAGACTTGGGATCGGGAGAGGTGAGAGGTACTTCTGGGGTAGGGGTAAAATCCTGTAATCCTGGAAGGACCACCGGTGGCGAAGGCGTCTGACTAGAACGGATCCGACGGTGAGGGACGAAGCCCTGGGGATCAAACAGG
>OTU42358<br>TACCCGCGCCCCGAGTGGTGGTCGATATTATTGAGCCTAAAACGTCCGTAGCCGGTCTTCTAAATCCTTGGGTAAATCGGGCAGCTTAACTGTCCGAAGTCCGGGGAGACTGGAAGACTTGGGACCGGGAGAGGTCAGAGGTACTTCTGGGGTAGGGGTAAAATCCTGTAATCCTAGAAGGACCACCGGTGGCGAAGGCGTCTCACTAGAACGAATCCGACGCTGAGGCGCGAAAGCGTGGGGAGCAAACAGG
>OTU42363<br>TACCCGCGCCCCGAGTGGTGGTCGATATTATTGAGCCTAAAACGTCCGTAGCCGGTCTTCTAAATCCTTGGGTAAATCGGGCAGCTTAACTGTCCGAAGTCCGGGGAGACTGGGAGACTTGGGACCGGGAGAGGTCAGAGGTACTTCTGGGGTAGGGGTAAAATCCTGTAATCCTGGAAGGACCACCGGTGGCGTAGGCGTCTCACCAGAACGGCTCCGACAGTGAGGGACGAAGCCCTGGGGCGCAAACGGG
>OTU42365<br>TACCCGCGCCCCTAGTGGTGGTCGATATTATTGAGCCTAAAACGTCCGTAGCCGGTCTTCTAAATCCTTGGGTAAATCGGGCAGCTTAACTGTCCGAAGTCCGGGGAGACTGGAAGACTTGGGACCGGGAGAGGTTAGCGGTACTCCCAGGGTAGGGGTGAAATCCTGTAATCCTGGAAGGACCACCGGTGGCGAAGGCGTCTGACTAGAACGGATCCGACGGTGAGGGACGAAGCCCTGGGGCGCAAACGGG
>OTU42368<br>TACCCGCGGCCCAAGTGGTGGTCGATATTATTGAGCCTAAAACGTCCGTAGCCGGTTTTGTAAATCCTTGGGTAAATCGACCAGCTTAACTGTTCGAAGTCCGGGGAGACTGCAAGACTTGGGATCGGGAGAGGTCAGAGGTACTTCTGGGGTAGGGGTAAAATCCTGTAATCCTGGAAGGACCACCGGTGGCGAAGGCGTCTGACTAGAACGGATCCGACGGTGAGGGACGAAGCCCTGGGGATCAAACAGG
>OTU42371<br>TACCCGCGGCCCGAGTGGTGGTCGATATTATTGAGCCTAAAACGTCCGTAGCCGGTTTTGTAAATCCTTGGGTAAATCGACCAGCTTAACTGTTCGAAGTCCGGGGAGACTGCAAGACTTGGGATCGGGAGAGGTGAGAGGTACTTCTGGGGTAGGGGTAAAATCCTGTAATCCTAGAAGGACCACCGGTGGCGAAGGCGTCTCACCAGAACGGCTCCGACAGTGAGGGACGAAGCCCTGGGGCGCAAACGGG
>OTU45367<br>TACGTAGGGGGCGAGCGTTATCCGGAATTATTGAGCCTAAAACGTCCGTAGCCGGTCTTCTAAATCCTTGGGTAAATCGGGCAGCTTAACTGTCCGAAGTCCGGGGAGACTGGGAGACTTGGGACCGGGAGAGGTCAGAGGTACTTCTGGGGTAGGGGTAAAATCCTGTAATCCTGGAAGGACCACCGGTGGCGAAGGCGTCTGACTAGAACGGATCCGACGGTGAGGGACGAAGCCCTGGGGCGCAAACGGG

>OTU47307<br>TACCCGCGCCCCTAGTGGTGGTCGATATTATTGAGCCTAAAACGTCCGTAGCCGGTCTTCTAAATCCTTGGGTAAATCGGGCAGCTTAACTGTGGGAATT<br>GCTGGAGATACTATTAGGCTTGAGGTCGGGAGAGGTTAGCGGTACTCCCAGGGTAGGGGTGAAATCCTATAATCCTGGGAGGACCACCGGTGGCGAAG<br>GCGTCTGACTAGAACGGATCCGACGGTGAGGGACGAAGCCCTGGGGCGCAAACGGG
>OTU50349<br>TACCCGCGCCCCGAGTGGTGGTCGATATTATTGAGCCTAAAACGTCCGTAGCCGGTCTTCTAAATCCTTGGGTAAATCGGGCAGCTTAACTGTCCGAAGT<br>CCTGGGAGACTTGGGACCGGGAGAGGTCAGAGGTACTTCTGGGGTAGGGGTAAAATCCTGTAATCCTGGAAGGACCACCGGTGGCGAAGGCGTCTGAC<br>TAGAACGGATCCGACGGTGAGGGACGAAGCCCTGGGGCGCAAACGGG
>OTU93191<br>CACCGGCAGCTCAAGTGGTGGCCATTATTATTGAGCCTAAAACGTCCGTAGCCGGTCTTCTAAATCCTTGGGTAAATCGGGCAGCTTAACTGTCCGAAGT<br>CCGGGGAGACTGGAAGACTTGGGACCGGGAGAGGTCAGAGGTACTTCTGGGGTAGGGGTAAAATCCTGTAATCCTAGAAGGACCACCGGTGGCGAAGG<br>CGTCTCACTAGAACGAATCCGACGGTGAGGGACGAAGCCCTGGGGCGCAAACGGG
>OTU93217<br>CACCGGCAGCTCAAGTGGTGGCCATTTTTATTGAGCCTAAAACGTCCGTAGCCGGTCTTCTAAATCCTTGGGTAAATCGGGCAGCTTAACTGTCCGAAGT<br>CCGGGGAGACTGGGAGACTTGGGACCGGGAGAGGTCAGAGGTACTTCTGGGGTAGGGGTAAAATCCTGTAATCCTGGAAGGACCACCGGTGGCGAAGG<br>CGTCTGACTAGAACGGATCCGACAGTGAGGGACAAAAGCTGGGGGAGCAAACCGG
>OTU93532<br>CACCGGCGGCCCGAGTGGTGATCGTGATTATTGAGCCTAAAACGTCCGTAGCCGGTTTTGTAAATCCTTGGGTAAATCGACCAGCTTAACTGTTCGAAGT<br>CCGGGGAGACTGCAAGACTTGGGATCGGGAGAGGTGAGAGGTACTTCTGGGGTAGGGGTAAAATCCTGTAATCCTAGAAGGACCACCGGTGGCGAAGG<br>CGTCTCACTAGAACGAATCCGACGGTGAGGGACGAAGCCCTGGGGCGCAAACGGG
>OTU93561<br>CACCGGCGGCCCGAGTGGTGATCGTGATTATTGGGTCTAAAACGTCCGTAGCCGGTCTTGTAAATCCTTGGGTAAATCGACCAGCTTAACTGTTCGAAGT<br>CCGGGGAGACTGCAAGACTTGGGATCGGGAGAGGTCAGAGGTACTTCTGGGGTAGGGGTAAAATCCTGTAATCCTAGAAGGACCACCGGTGGCGAAGG<br>CGTCTGACTAGAACGAATCCGACGGTGAGGGACGAAGCCCTGGGGCGCAAACGGG
>OTU137628<br>TACCCGCGCCCCAAGTGGCGGTCGATATTATTGAGCCTAAAACGTCCGTAGCCGGTCTTCTAGATCCTTGGGTAAATCGGGCAGCTCAACTGCCCGAATT<br>CCGGGGAGACTGGGAGACTTGGGGCCGGGAGAGGTCAGAGGTACTTCTGGGGTAGGGGTAAAATCCTGTAATCCTGGAAGGACCACCGGTGGCGAAGG<br>CGTCTGACTAAAACGGATCCGACGGTGAGGGACGAAGCCCTGGGGCGCAAACGGG
>OTU137784<br>TACCCGCGCCCCAAGTGGTGGTCGATATTATTAAGCCTAAAACGTCTGTAGCCGGTCTTCTAGATCCTTGGGTAAATCGGGCAGCTCAACTGTCCGGATT<br>CCGGGGAGACTGGGAGACTTGGGACCGGGAGAGGTCAGAGGTACTTCTGGGGTAGGGGTAAAATCCTGTAATCCTGGAAGGACCACCGGTGGCGAAGG<br>CGTCTGACTAGAACGGATCCGACGGTGAGGGACGAAGCCCTGGGGCGCAAACGGG
>OTU138153<br>TACCCGCGCCCCAAGTGGTGGTCGATATTATTGAGCCTAAAACGTCCGTAGCCGGTCTTCTAAATCCTTGGGTAAATCGGGCAGCTTAACTGTCCGAAGT<br>CCGGGGAGACTGGGAGACTTGGGACCGGGAGAGGTCAGAGGTACGTCTGGGGTAGGGGGAAAATCCTGTAATCCTGGAAGGACCACCGGTGGCGAAG<br>GCGTCTGACTAGAACGGATCCGATGGGGAGGGACGAAGCCCTGGGGCGCAAACGGG
>OTU138161<br>TACCCGCGCCCCAAGTGGTGGTCGATATTATTGAGCCTAAAACGTCCGTAGCCGGTCTTCTAAATCCTTGGGTAAATCGGGCAGCTTAACTGTCCGAAGT<br>CCGGGGAGACTGGGAGACTTGGGACCGGGAGAGGTCAGAGGTACTTCTGGGGTAGGGGTAAAATCCTGTAATCCTGGACGGACGACCGGTGGCGAAGG<br>CGTCTGACTAGAACGGAGCCGACGGGGAGGGACGAAGCCCTGGGGCGCAAACGGG
>OTU138577<br>TACCCGCGCCCCACGTGGTGGTCGATCATATTGAGCCTAAAACGTCCGTAGCCGGTCTTCTAAATCCTTGGGTAAATCGGGCAGCTTAACTGTCCGAAGT<br>CCGGGGAGACTGGGAGACTTGGGACCGGGAGAGGTCAGAGGTACTTCTGGGGTAGGGGTAAAATCCTGTAATCCTGGAAGGACCACCGGTGGCGAAGG<br>CGTCTGACTAGAACGGATCCGACGGTGAGGGACGAAGCCCTGGGGCGCAAACGGG
>OTU138814<br>TACCCGCGCCCCGAGTGGTGGTCGATATTATTGAGCCTAAAACGTCCGTAGCCGGTCTTCTAAATCCTTGGGTAAATCGGCCAGCTTAACTGACCGAATT<br>CCGGGGAGACTGCCAAACTTGGGACCGGGAGAGGCTAGAGGTACTTCTGGGGTAGGGGTAAAATCCTGTAATCCTGGAAGGACCACCGGTGGCGAAGG<br>CGTCTAGCTAGAACGGATCCGACGGTGAGGAACGAAGCCCTGGGGCGCAAACGGG
>OTU138856<br>TACCCGCGCCCCGAGTGGTGGTCGATATTATTGAGCCTAAAACGTCCGTAGCCGGTCTTCTAAATCCTTGGGTAAATCGGGCAGCTTAACTGTCCGAAGT<br>CCGGGGAGACTGGAAGACTTGGGACCGGGGGAGGTCAGAGGTACTTCTGGGGTAGGGGTAAAATCCTGTAATCCTGGAAGGACCACCGGTGGCGAAGG<br>CGTCTGACTAGAACGGATCCGGCGGTGAGGGACGAAGCCCTGGGGCGCAAACAGG
>OTU139058<br>TACCCGCGCCCCGAGTGGTGGTCGATATTATTGAGCCTAAAACGTTCGTAGCCGGTCTCCTAAATCCCTGGGTAAATCGGGCAGCTTAACTGTCCGAAGT<br>CCGGGGAGACTGGGAGACTTGGGACCGGGAGAGGTCAGAGGTACTTCTGGGGTAGGGGTAAAATCCTGTAATCCTGGAAGGACCACCGGTGGCGAAGG<br>CGTCTGACTAGAACGGATCCGACGGTGAGGGACGAGGCCCTGGGGCGCAAACGGG
>OTU139250<br>TACCCGCGCCCCTAGGGGTGGTCGATATTATTGAGCCTAAAACGTCCGTAGCCGGTCGTCTAAATCCGTGGGTAAATCGGGCAGCTTAACTGTCCGAAGT<br>CCGGGGAGACTGGGAGACTTGGGACCGGGAGAGGTCAGAGGTACTTCTGGGGTAGGGGTAAAATCCTGTAATCCTGGAAGGACCACCGGTGGCGAAGG<br>CGTCTGACTAGAACGGATCCGACGGTGAGGGACGAAGCCCTGGGGCGCAAACGGG
>OTU139311<br>TACCCGCGCCCCTAGTGGTGGTAGATATTATTGAGACTAAAACGTCCGGAGCCGGTCTTCTAAATCCTTGGGTAAATCGGGCAGCTTAACTGTCCGAAGT<br>CCGGGGAGACTGGGAGACTTGGGACCGGGAGAGGTCAGAGGTACTTCTGGTGTAGGGGTAAAATCCTGTAATCCTGGAAGGACCACCGGTGGCGAAGG<br>CGTCTGACTAGAACGGATCCGACGGTGAGGGACGAAGCCCTGGGGCGCAAACGGG
>OTU139582<br>TACCCGCGCCCCTAGTGGTGGTCGATATTATTGAGCCTAAAACGTCCGTAGCCGGTCATCTAAATCCTTGGGTAAATCGGGCAGCTTAACTGTCCGAAGT<br>CCGGGGAGACTGGAAGACTTGGGACCGGGAGAGGTCAGAGGTACTTCTGGGGTAGGGGTAAAATCCTGTAATCCTGGGAGGACCACCTGTGGCGAAGG<br>CGGCTAACTGGAACGGATCCGACGGTGAGGGACGAAGCCCTGGGGCGCAAACGGG
>OTU139596<br>TACCCGCGCCCCTAGTGGTGGTCGATATTATTGAGCCTAAAACGTCCGTAGCCGGTCGTCTAAATCCTTGGGTAAATCGGGCTGCTTAACTGTCCGAAGT<br>CCGGGGAGACTGGGAGACTTGGGACCGGGAGAGGTCAGAGGTACTTCTGGGGTAGGGGTAAAATCCTGTAATCCTGGAAGGACCACCGGTGGCGAAGG<br>CGTCTGACTCGAACGGATCCGACGGTGAGGGACGAAGCCCTGGGGCGCAAACGGG
>OTU139635<br>TACCCGCGCCCCTAGTGGTGGTCGATATTATTGAGCCTAAAACGTCCGTAGCCGGTCTTCTAAATCCGTGGGTAAATCGGGCCGCTTAACTGTCCGAAGT<br>CCGGGGAGACTGGGAGACTTGGGACCGGGAGAGGTCAGAGGTACTTCTGGGGTAGGGGTAAAATCCTGTAATCCTGGAAGGACCACCGGTGGCGAAGG<br>CGTCTGACTAGAACGGATCCGACGGTGAGGGACGAAGCCCTGGGGCGCAAACAGG

>OTU139663<br>TACCCGCGCCCCTAGTGGTGGTCGATATTATTGAGCCTAAAACGTCCGTAGCCGGTCTTCTAAATCCTTGGGTAAATCAGGCAGCTTAACTGTCCGAAGT<br>CCGGGGAGACTGGGAGACTTGGGACCGGGAGAGGTCAGTGGTACTTCTGGGGTAGGGGTAAAATCCTGTAATCCTGGAAGGACCACCGGTGGCGAAGG<br>CGTCTGACTAGAACGGATCCGACGGTGAGGGACGAAGCCCTGGGGCGCAAACAGG
>OTU139667<br>TACCCGCGCCCCTAGTGGTGGTCGATATTATTGAGCCTAAAACGTCCGTAGCCGGTCTTCTAAATCCTTGGGTAAATCGACCAGCTTAACTGTTCGAAGTC<br>CGGGGAGACTGGGAGACTTGGGACCGGGAGAGGTCAGAGGTACTTCTGGGGTAGGGGTAAAATCCTGTAATCCTGGAAGGACCACCGGTGGCGAAGGC<br>GTCTGACTAGAACGGATCCGACGGTGAGGGACGAAGCCCTGGGGCGCAAACAGG
>OTU139671<br>TACCCGCGCCCCTAGTGGTGGTCGATATTATTGAGCCTAAAACGTCCGTAGCCGGTCTTCTAAATCCTTGGGTAAATCGGCTAGCTTAACTAACCGAATTC<br>CGAGGAGACTGTGGATCTTGGGACCGGGAGAGGTCAGAGGTACTTCTGGGGTAGGGGTAAAATCCTGTAATCCTAGAAGGACCACCGGTGGCGAAGGC<br>GTCTGACTAGAACGGATCCGACGGTGAGGGACGAATCCCTGGGGCGCAAACGGG
>OTU139707<br>TACCCGCGCCCCTAGTGGTGGTCGATATTATTGAGCCTAAAACGTCCGTAGCCGGTCTTCTAAATCCTTGGGTAAATCGGGCAGCTTAACTGTCCGAAGT<br>CCGGGGAGACAGGGAGACTTGGGACCGGGAGAGGTCAGAGGTACTTCTGGGGTAGGGGTAAAATCCTGTAATCCTGGAAGGACCACCGGTGGCGAAG<br>GCGTCTCACTAGAACGAATCCGACGGTGAGGGACGAAGCCCTGGGGAGCAAACAGG
>OTU139710<br>TACCCGCGCCCCTAGTGGTGGTCGATATTATTGAGCCTAAAACGTCCGTAGCCGGTCTTCTAAATCCTTGGGTAAATCGGGCAGCTTAACTGTCCGAAGT<br>CCGGGGAGACTATTAGGCTTGAGGTCGGGAGAGGTTAGCGGTACTCCCAGGGTAGGGGTGAAATCCTGTAATCCTGGAAGGACCACCGGTGGCGAAGG<br>CGTCTGACTAGAACGGATCCGACGGTGAGGGACGAAGCCCTGGGGCGCAAACGGG
>OTU139763<br>TACCCGCGCCCCTAGTGGTGGTCGATATTATTGAGCCTAAAACGTCCGTAGCCGGTCTTCTAAATCCTTGGGTAAATCGGGCAGCTTAACTGTCCGAAGT<br>CCGGGGAGACTGGAAGACTTGGGACCGGGAGAGGTCAGAGGTACTTCTGGGGTAGGGGTAAAATCCTGTAATCCTGGAAGGACCACCGGTGGCGAAGG<br>CGGCTAACTGGAACGGACCTGACGGTGAGTAACGAAGCCCTGGGGCGCAAACGGG
>OTU139857<br>TACCCGCGCCCCTAGTGGTGGTCGATATTATTGAGCCTAAAACGTCCGTAGCCGGTCTTCTAAATCCTTGGGTAAATCGGGCAGCTTAACTGTCCGAAGT<br>CCGGGGAGACTGGGAGACTTGGGACCGGGAGAGGTCAGAGGCACTTCTGGGGTAGGGGTAAAATCCTGTAATCCTGGAAGGACCACCGGTGGCGAAG<br>GCGTCTGACTAGAACGGATCCGACGGTGAGGGACGAAGCCCTGGGGCGCAAATAGG
>OTU139866<br>TACCCGCGCCCCTAGTGGTGGTCGATATTATTGAGCCTAAAACGTCCGTAGCCGGTCTTCTAAATCCTTGGGTAAATCGGGCAGCTTAACTGTCCGAAGT<br>CCGGGGAGACTGGGAGACTTGGGACCGGGAGAGGTCAGAGGTACTTCGGGGGGAGGAGTAAAATCCTGTAATCCTAGAAGGACCACCGGTGGCGAAG<br>GCGTCTCACTAGAACGAATCCGACGGTGAGGGACGAAGCCCTGGGGCGCAAACGGG
>OTU139868<br>TACCCGCGCCCCTAGTGGTGGTCGATATTATTGAGCCTAAAACGTCCGTAGCCGGTCTTCTAAATCCTTGGGTAAATCGGGCAGCTTAACTGTCCGAAGT<br>CCGGGGAGACTGGGAGACTTGGGACCGGGAGAGGTCAGAGGTACTTCTGGGGTAGGGGGAAGATCCTGTAATCCTGGAAGGACCACCGGTGGCGAAG<br>GCGTCTGACTAGAGCGGATCCGACGGGGAGGGACGAAGCCCTGGGGCGCAAACGGG
>OTU139872<br>TACCCGCGCCCCTAGTGGTGGTCGATATTATTGAGCCTAAAACGTCCGTAGCCGGTCTTCTAAATCCTTGGGTAAATCGGGCAGCTTAACTGTCCGAAGT<br>CCGGGGAGACTGGGAGACTTGGGACCGGGAGAGGTCAGAGGTACTTCTGGGGTAGGGGTAAAATCCTATAATCCTGGGAGGACCACCTGTGGCGAAGG<br>CGTCTGACTAGAACGGATCCGACGGTGAGGGACGAGGCCCTGGGGCGCAAACGGG
>OTU139890<br>TACCCGCGCCCCTAGTGGTGGTCGATATTATTGAGCCTAAAACGTCCGTAGCCGGTCTTCTAAATCCTTGGGTAAATCGGGCAGCTTAACTGTCCGAAGT<br>CCGGGGAGACTGGGAGACTTGGGACCGGGAGAGGTCAGAGGTACTTCTGGGGTAGGGGTGAAATCCTGTAATGCTGGAAGGAGCACCGGTGGCGAAG<br>GCGTCTGACTAGAACGGATCCGACGGGGAGGGACGAAGCCCTGGGGCGCAAACGGG
>OTU139906<br>TACCCGCGCCCCTAGTGGTGGTCGATATTATTGAGCCTAAAACGTCCGTAGCCGGTCTTCTAAATCCTTGGGTAAATCGGGCAGCTTAACTGTCCGAAGT<br>CCGGGGAGACTGGGAGACTTGGGACCGGGAGAGGTCAGGGGTACTTCTGGGGTAGGGGGAAAATCCTGTAATCCTGGAAGGACCACCGGTGGCGAAG<br>GCGTCTGACTAGAACGGACCCGACGGTGAGGGACGAAGCCCTGGGGCGCAAACGGG
>OTU139935<br>TACCCGCGCCCCTAGTGGTGGTCGATATTATTGAGCCTAAAACGTCCGTAGCCGGTCTTCTAAATCCTTGGGTAAATCGGGCAGCTTAACTGTCCGAAGT<br>CCGGGGAGGCTGGGAGACTTGGGACCGGGAGAGGTCAGAGGTACTTCTGGGGTAGGGGTAAAATCCTGTAATCCTGGAAGGACCACCGGTGGCGAGGG<br>CGTCTGACTAGAACGGATCCGACGGTGAGGGACGAAGCCCTGGGGCGCAAACAGG
>OTU140162<br>TACCCGCGCCCCTAGTGGTGGTCGATATTATTGAGCCTAACACGTCCGGAGCCGGTCTTCTACATCCTGGGGTAAATCGGGCAGCTTAACTGTCCGAAGT<br>CCGGGGAGACTGGAAGACTGGGGACCGGGAGAGGTCAGAGGTACTTCTGGGGTAGGGGTAAAATCCTGTAATCCTGGAAGGACCACCGGTGGCGAAG<br>GCGTCTGACTAGAACGGATCCGACGGTGAGGGACGAAGCCCTGGGGCGCAAACGGG
>OTU140273<br>TACCCGCGCCCCTAGTGGTGGTCGATCCTATTGAGCCTAAAACGTCCGTAGCCGGTCTTCTAAATCCTTGGGTAAATCGGGCAGCTTAACTGTCCGAAGT<br>CCGGGGAGACTGGGAGACTTGGGACCGGGAGAGGTCAGAGGTACGTCTGGGGTAGGGGTAAAATCCTGTAATCCTGGAAGGACCACCGGTGGCGAAG<br>GCGTCTGACTAGAACGGATCCGACGGTGAGGGACGAAGCCCTGGGGCGCAAACGGG
>OTU140392<br>TACCCGCGCCCCTAGTGGTGTTCGATATTATTGAGCCTAAAACGTCCGTAGCCGGTCTTCTAAATCCTTGGGTAAATCGGGCAGCTTAACTGTCCTAAGTC<br>CGGGGAGACTGGGAGACTTGGGACCGGGAGAGGTCAGAGGTACTTCTGGGGTAGGGGTAAAATCCTGTAATCCTGGAAGGACCACCGGTGGCGAAGGC<br>GTCTGACTAGAACGGATCAGACGGTGAGGGACGAAGCCCTGGGGCGCAAACGGG
>OTU140596<br>TACCCGCGGCCCGAGTAGTGGTCGATATTATTGAGCCTAAAACGTCCGTAGCCGGTCTTCTAAATCCTTGGGTAAATCGGGCAGCTTAACTGTCCGAAGT<br>CCGGGGAGACTGGAAGACTTGGGACCGGGAGAGGTCAGAGGTACTTCTGGGGTAGGGGTAAAATCCTGTAATCCTGGAAGGACCACCGGTGGCGAAGG<br>CGTCTGACTAGAACGGATCCGACGGTGAGGGACAAAGCCCTGGGGCGCAAACGGG
>OTU140729<br>TACCCGCGGCCCGAGTGGTGGTCGATATTATTGAGCCTAAAACGTCCGTAGCCGGTCTTCTAAATCCTTGGGTAAATCGACCAGCTTAACTGTCCGAAGT<br>CCGGGGAGACTGCAAGACTTGGGATCGGGAGAGGTGAGAGGTACTTCTGGGGTAGGGGTAAAATCCTGTAATCCTAGAAGGACCACCGGTGGCGAAGG<br>CGTCTCACTAGAACGAATCCGACGGTGAGTAACGAAGGCCAGGGGCGCGAACCGG
TU407971<br>TACCCGCGCCCCGAGTGGTGGTCGATATTATTGAGCCTAAAACGTCCGTAGCCGGTCTTCTAAATCCTTGGGTAAATCGGGCAGCTTAACTGTGGGAATT<br>GCTGGAGATACTATCAGGCTTGAGGCCGGGAGAGGTTAGGGGTACTCCCAGGGTAGGGGTGAAATCCTGTAATCCTGGAAGGACCACCGGTGGCGAAG<br>GCGTCTGACTAGAACGGATCCGACGGTGAGGGACGAAGCCCTGGGGCGCAAACGGG

Table S4. Results of the quantification of *Methanomassiliicoccales* by qPCR in the different samples. Quantification is expressed as the number of copies of the target gene × 10^3 per gram of wet matrix. Legend, NQ: non-quantifiable, *i.e.* a sample with no signal or less than 3 Ct difference from the extraction control, in qPCR experiments where 90 < PCR efficiency < 110% and r^2 of standard curve >0.99; Below the detection limit: Less than 10^3 gene copies per ml or gram of wet weight of sample.

Sample	*Methanomassiliicoccales* 16S rRNA gene copies per gram of wet weight (× 10^3)
COMRA	NQ
DOUR	6.51 ± 4.84
HM1	Below the detection limit
HM2	Below the detection limit
HM3	Below the detection limit
HM4	Below the detection limit
KERG	6.41 ± 0.93
KRY150	Below the detection limit
KRY238	NQ
MOUG1	75.02 ± 4.59
MOUG2	62.63 ± 5.10
MOUG3	1.86 ± 0.19
MOUG4	Below the detection limit
MOUG5	NQ
MOZ1	1.62 ± 1.02
MOZ2	1.76 ± 0.5
MOZ3	Below the detection limit
PAV60	13.2 ± 0.69
PAV70	0.45 ± 0.18
PAV80	3.79 ± 2.48
PENF	53.24 ± 4.27
XIA	Below the detection limit

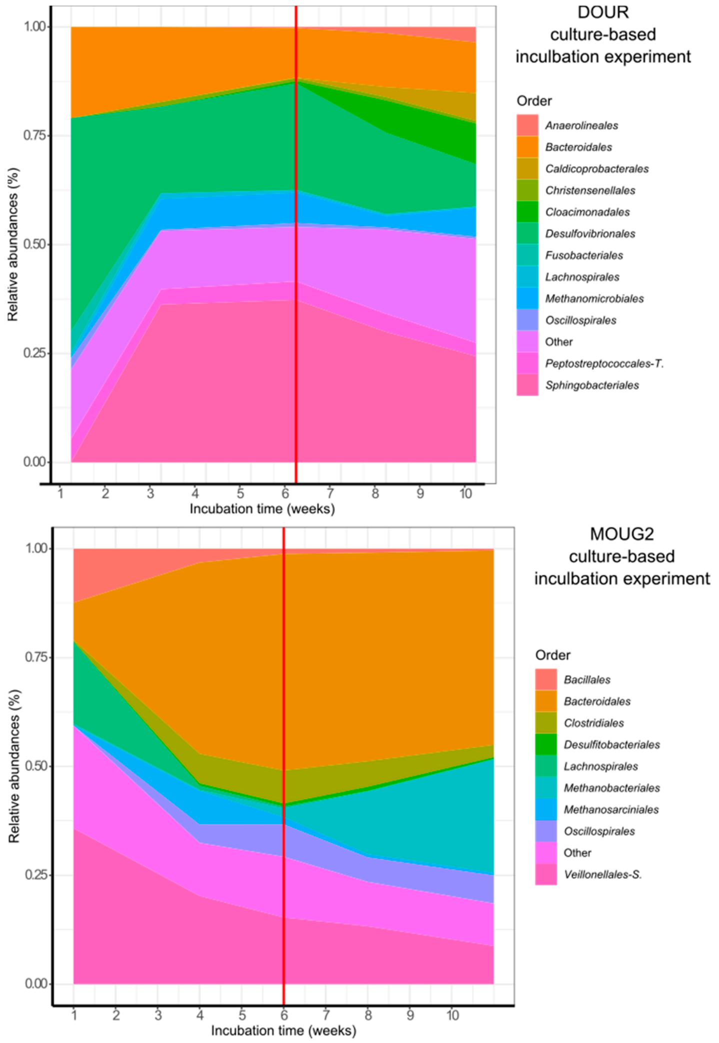

Figure S1. Trends in microbial diversity revealed by metabarcoding in DOUR (top) and MOUG2 (bottom) substrate-amended slurries, over time. Other: microbial diversity representing less than 5% of the sequences in the culture-based experiment.

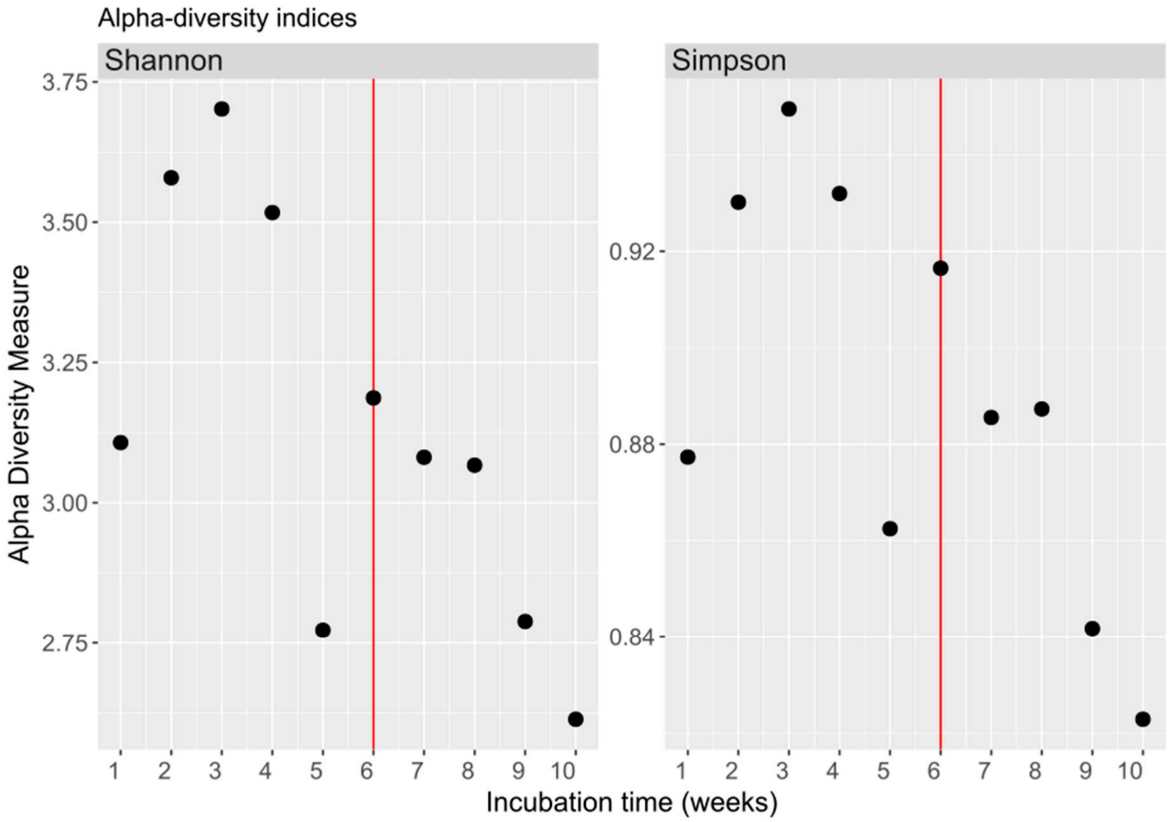

**Incubation**	**1**	**2**	**3**	**4**	**5**	**6**	**7**	**8**	**9**	**10**
Shannon	3.1072	3.5791	3.7017	3.517	2.7728	3.1868	3.0811	3.0669	2.7884	2.6138
Simpson	0.8773	0.9302	0.9495	0.932	0.8625	0.9165	0.8855	0.8873	0.8417	0.8229

Figure S2. Evolution of the prokaryotic alpha-diversity during the incubation of the PENF sample, in the culture-based incubation experiment. The vertical red line indicates an addition of TMA, methanol and H_2/CO_2 after 6 weeks of incubation.

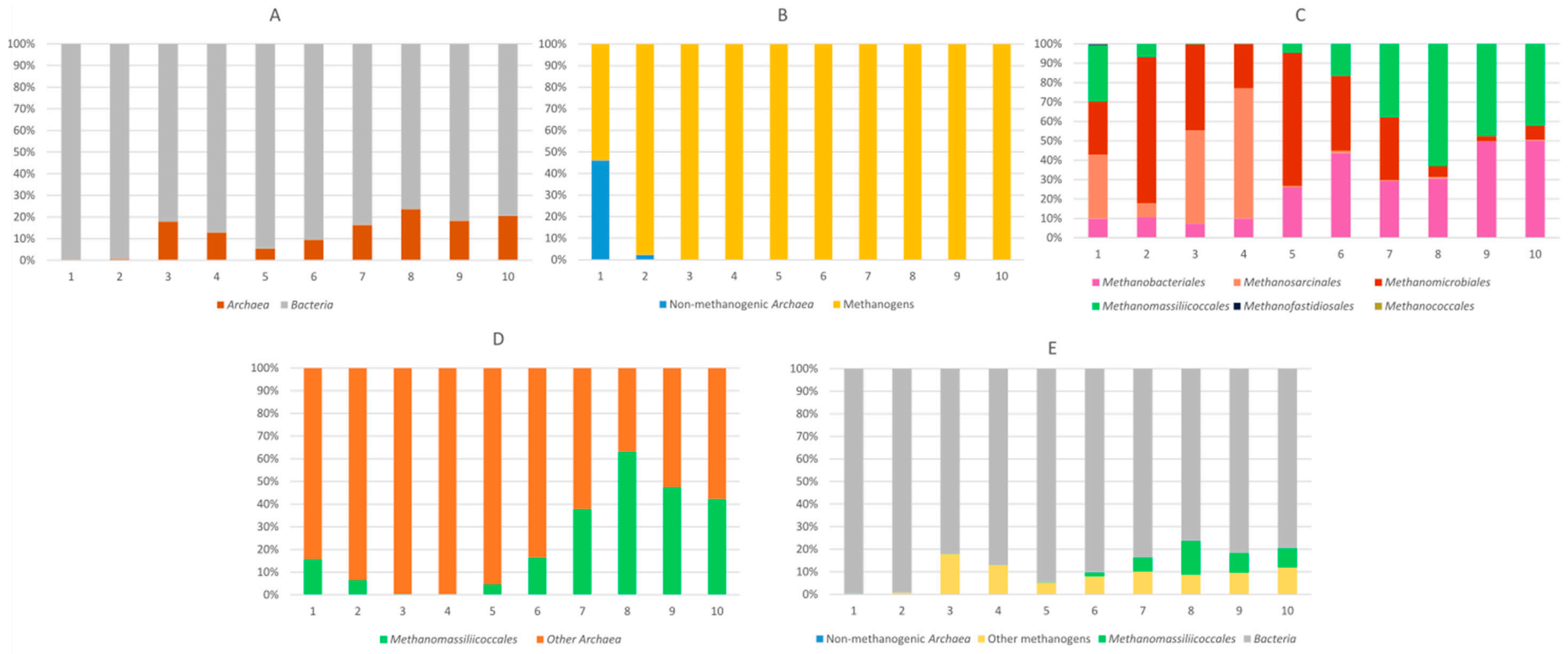

Figure S3. Microbial diversity patterns revealed by metabarcoding (16S rRNA gene copy numbers) in the PENF substrate-amended slurry over time. (A) Relative abundance of *Archaea* with respect to *Bacteria* sequences (B) Relative abundance of methanogen's and non-methanogen's archaeal sequence reads. (C) Relative abundance of sequences affiliated to the various methanogen's orders. (E) Relative abundance of sequences of *Methanomassiliicoccales* with respect to other *Archaea* sequences. (E) Relative abundance of *Methanomassiliicoccales* sequences with respect to non-methanogenic *Archaea*, other methanogens and *Bacteria* sequences..

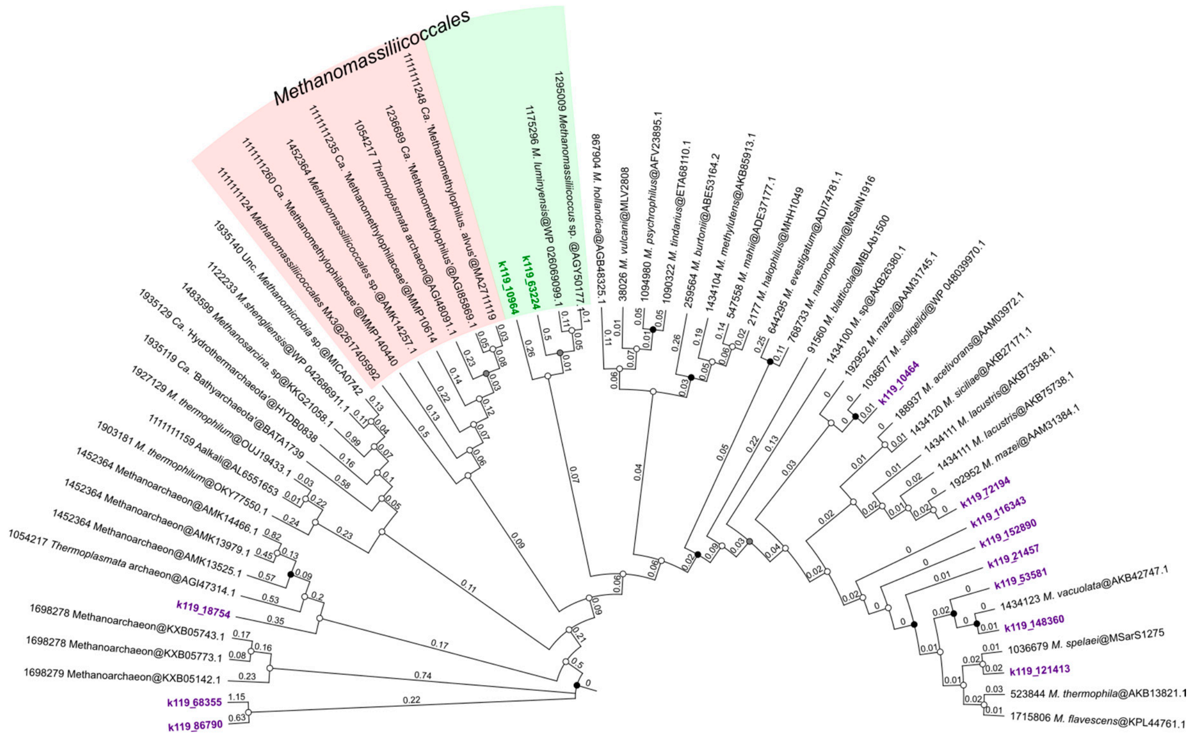

Figure S4. Phylogenetic tree showing the relationships between the trimethylamine methyltransferases (*mttB*) sequences extracted from the metagenome (in bold), and closest *mttB* sequences in public databases. This reconstruction was performed using the PhyML algorithm. Two *mttB* reconstructed sequences were affiliated to *Methanomassiliicoccales* (bold green) while the other 11 *mttB* sequences where affiliated to other methanoarchaea (bold purple). Accession numbers are preceded by a "@".

Table S5. Taxonomic assignation of the taxa non-randomly associated with *Methanomassiliicoccales* OTUs and their reported metabolic features, within the PENF substrate-amended slurry. * the taxonomic affiliation does not allow to infer any possible metabolic characteristics; ** from genome annotation; ND : Not Determined; *Peptostreptococcales-T.*: *Peptostreptococcales-Tissierellales* ; *Veillonellales-S.*: *Veillonellales-Selenomonadales*.

Origin	Percolation threshold	OTU source	Weight	Targeted OTU	Consensus Silva138, RDP database taxonomic affiliations			Representative reported to maintained (putative) partnership with methanogens	Reported metabolic products	References
					Order	Family	Genus			
Culture-based incubation experiment (PENF)	0.79	OTU21	0.96	OTU2	*Bacteroidales*	*Dysgonomonadaceae*	Multi-affiliation	No	Acids, acetate**	[24,25]
			0.95	OTU52	*Synergistales*	*Synergistaceae*	Unknown genus	Yes	Short-chain fatty acids	[26]
			0.95	OTU6	Unknown order	*Hungateiclostridiaceae*	*Ruminiclostridium*	ND	Acetate, ethanol, H_2, CO_2, lactate, propionate, butyrate, and others	[27]
			0.93	OTU90	*Synergistales*	*Synergistaceae*	Unknown genus	Yes	Short-chain fatty acids	26]
			0.92	OTU213	*Christensenellales*	*Christensenellaceae*	*Christensenellaceae* R-7 group	Yes	Acids, butyrate, H_2, CO_2	[28–31]
			0.91	OTU44	*Methanobacteriales*	*Methanobacteriaceae*	*Methanobacterium*	No	CH_4	
			0.91	OTU8	*Bacteroidales*	*Rikenellaceae*	Bact-08	No	Acetate, alcohols, succinate	[30,32]
		OTU101	0.83	OTU521	*Peptococcales*	*Peptococcaceae*	Unknown genus	Yes	Acetate	[33]
			0.83	OTU693	*Peptostreptococcales-T.*	*Anaerovoracaceae*	*Anaerovorax*	Yes	Acetate, H_2, butyrate	[34]
Bulk samples	0.70	OTU21	0.73	OTU1052	*Woesearchaeales*	Unknown family	Unknown genus	Yes	Acetate, H_2	[35]
			0.73	OTU2361	*Spirochaetales*	*Spirochaetaceae*	Unknown genus	Yes	Acetate, H_2, CO_2	[36]
			0.71	OTU1174	*Woesearchaeales*	Unknown family	Unknown genus	Yes	Acetate, H_2	[37]
		OTU30	0.85	OTU481	Unknown order	Unknown family	Unknown genus	*	*	

			0.84	OTU360	Unknown order	Unknown family	Unknown genus	*	*	
			0.80	OTU1228	*Omnitrophales*	*Omnitrophaceae*	*Candidatus* 'Omnitrophus'	*	*	
			0.79	OTU1624	*Desulfatiglandales*	*Desulfatiglandaceae*	*Desulfatiglans*	No	CO_2	[38,39]
			0.77	OTU1749	MSBL5	Unknown family	Unknown genus	*	*	
			0.77	OTU206	*Aminicenantales*	Unknown family	Unknown genus	Yes	Acetate, H_2, CO_2	[33]
			0.73	OTU2574	Napoli-4B-65	Unknown family	Unknown genus	*	*	
			0.73	OTU2864	*Omnitrophales*	*Omnitrophaceae*	*Candidatus* 'Omnitrophus'	*	*	
			0.72	OTU2675	*Babeliales*	Unknown family	Unknown genus	No	ND	[40]
			0.71	OTU308	FW22	Unknown family	Unknown genus	*	*	
			0.70	OTU2279	*Desulfobulbales*	*Desulfocapsaceae*	Multi-affiliation	ND	ND	ND
		OTU101	0.87	OTU667	MBGD/DHVEG-1	Unknown family	Unknown genus	*	*	
			0.85	OTU2691	MBGD/DHVEG-1	Unknown family	Unknown genus	*	*	
			0.76	OTU2638	*Anaerolineales*	*Anaerolineaceae*	Unknown genus	Yes	Acetate	[41]
			0.72	OTU21041	*Geobacterales*	*Geobacteraceae*	Multi-affiliation	Yes	Acetate	[33,42]
			0.72	OTU2591	MBGD/DHVEG-1	Unknown family	Unknown genus	*	*	
			0.70	OTU2584	*Veillonellales-S.*	*Sporomusaceae*	Unknown genus	Yes	Acetate, $R-CH_3$	[35]
		OTU926	0.81	OTU1049	*Methanomicrobiales*	*Methanoregulaceae*	*Methanoregula*	No	CH_4	[43]
			0.80	OTU2449	*Peptostreptococcales-T.*	*Anaerovoracaceae*	*Anaerovorax*	Yes	Acetate, H_2, butyrate	[34]
			0.71	OTU2815	*Woesearchaeales*	Unknown family	Unknown genus	Yes	Acetate, H_2	[37]
			0.71	OTU2148	RBG-13-54-9	Unknown family	Unknown genus	*	*	
			0.70	OTU2706	*Anaerolineales*	*Anaerolineaceae*	*Leptolinea*	No	Acetate	[41]
Bulk samples	0.83	OTU30	0.85	OTU481	Unknown order	Unknown family	Unknown genus	*	*	
			0.85	OTU360	Unknown order	Unknown family	Unknown genus			
		OTU101	0.87	OTU667	MBGD/DHVEG-1	Unknown family	Unknown genus	*	*	
			0.85	OTU2691	MBGD/DHVEG-1	Unknown family	Unknown genus	*	*	

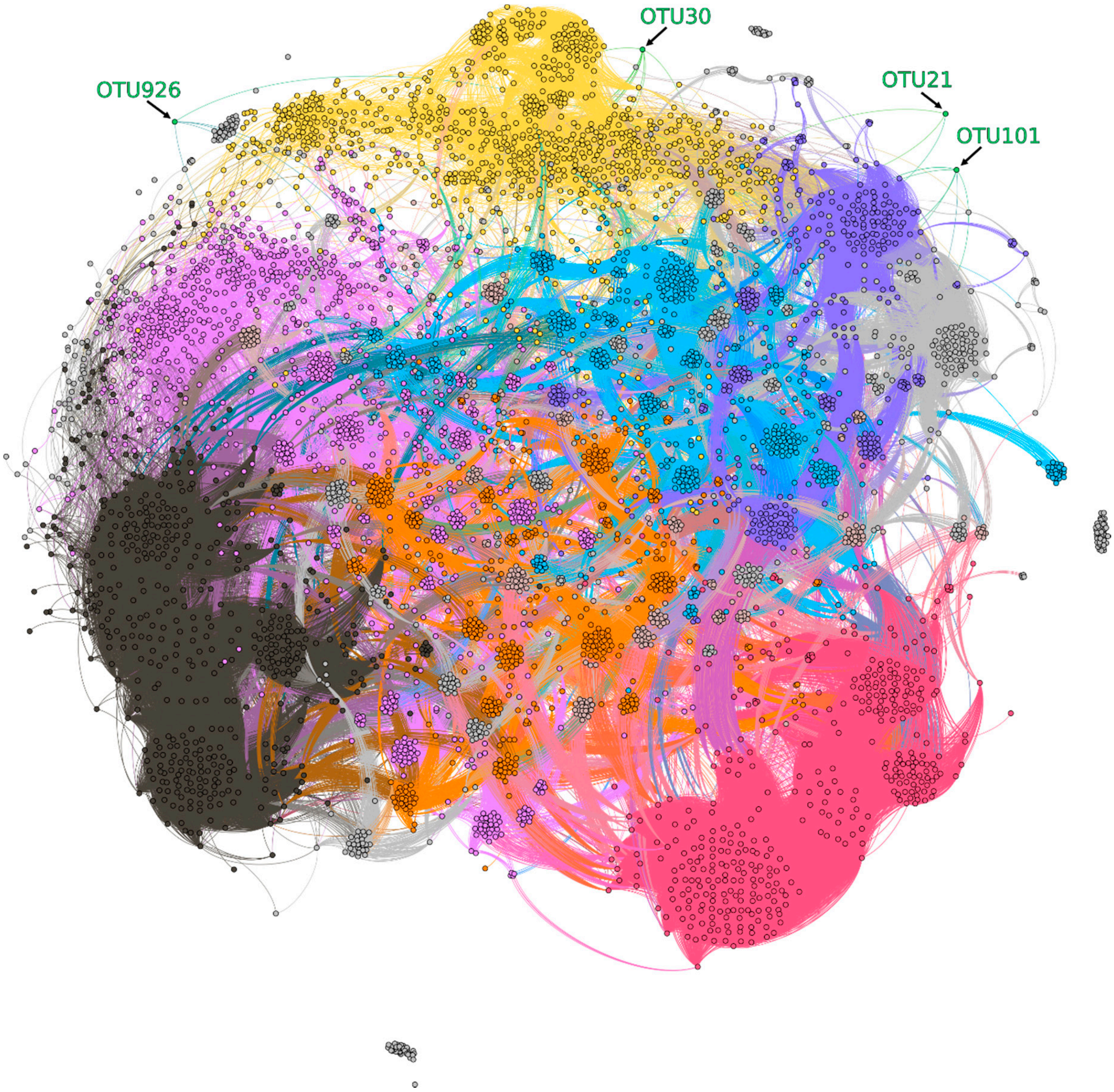

Figure S5. Co-occurrence network reconstructed based on 16S rRNA gene sequencing data from bulk environmental samples, based on a Spearman rank's correlation between OTUs and calculated with a percolation threshold of 0.70. Different colour nodes show different modules. *Methanomassiliicoccales* are in green.

References

1. Mihajlovski, A.; Doré, J.; Levenez, F.; Alric, M.; Brugère, J.-F. Molecular evaluation of the human gut methanogenic archaeal microbiota reveals an age-associated increase of the diversity. *Environ. Microbiol. Rep.* **2010**, *2*, 272–280, doi:10.1111/j.1758-2229.2009.00116.x.
2. L'Haridon, S.; Chalopin, M.; Colombo, D.; Toffin, L. *Methanococcoides vulcani* sp. nov., a marine methylotrophic methanogen that uses betaine, choline and N,N-dimethylethanolamine for methanogenesis, isolated from a mud volcano, and emended description of the genus *Methanococcoides*. *Int. Syst. Evol. Micr.* **2014**, *64*, 1978–1983, doi:10.1099/ijs.0.058289-0.
3. Zillig, W.; Holz, I.; Klenk, H.-P.; Trent, J.; Wunderl, S.; Janekovic, D.; Imsel, E.; Haas, B. *Pyrococcus woesei*, sp. nov., an ultra-thermophilic marine archaebacterium, representing a novel order, *Thermococcales*. *Syst. Appl. Microbiol.* **1987**, *9*, 62–70, doi:10.1016/S0723-2020(87)80057-7.
4. Lehours, A.-C.; Evans, P.; Bardot, C.; Joblin, K.; Gérard, F. Phylogenetic Diversity of *Archaea* and *Bacteria* in the Anoxic Zone of a Meromictic Lake (Lake Pavin, France). *Appl. Environ. Microbiol.* **2007**, *73*, 2016–2019, doi:10.1128/AEM.01490-06.
5. Yakimov, M.M.; Cono, V.L.; Spada, G.L.; Bortoluzzi, G.; Messina, E.; Smedile, F.; Arcadi, E.; Borghini, M.; Ferrer, M.; Schmitt-Kopplin, P.; et al. Microbial community of the deep-sea brine Lake Kryos seawater—Brine interface is active below the chaotropicity limit of life as revealed by recovery of mRNA. *Environ. Microbiol.* **2015**, *17*, 364–382, doi:10.1111/1462-2920.12587.
6. Alain, K.; Callac, N.; Ciobanu, M.-C.; Reynaud, Y.; Duthoit, F.; Jebbar, M. DNA extractions from deep subseafloor sediments: Novel cryogenic-mill-based procedure and comparison to existing protocols. *J. Microbiol. Methods* **2011**, *87*, 355–362, doi:10.1016/j.mimet.2011.09.015
7. Blankenberg, D.; Gordon, A.; Von Kuster, G.; Coraor, N.; Taylor, J.; Nekrutenko, A.; the Galaxy Team. Manipulation of FASTQ data with Galaxy. *Bioinformatics* **2010**, *26*, 1783–1785, doi:10.1093/bioinformatics/btq281.
8. Dridi, B.; Fardeau, M.-L.; Ollivier, B.; Raoult, D.; Drancourt, M. *Methanomassiliicoccus luminyensis* gen. nov., sp. nov., a methanogenic archaeon isolated from human faeces. *Int. J. Syst. Evol. Micr.* **2012**, *62*, 1902–1907, doi:10.1099/ijs.0.033712-0.
9. Iino, T.; Tamaki, H.; Tamazawa, S.; Ueno, Y.; Ohkuma, M.; Suzuki, K.; Igarashi, Y.; Haruta, S. *Candidatus* Methanogranum caenicola: A Novel Methanogen from the Anaerobic Digested Sludge, and Proposal of *Methanomassiliicoccaceae* fam. nov. and *Methanomassiliicoccales* ord. nov., for a Methanogenic Lineage of the Class *Thermoplasmata*. *Microbes Environ.* **2013**, *28*, doi:10.1264/jsme2.ME12189
10. Borrel, G.; O'Toole, P.W.; Harris, H.M.B.; Peyret, P.; Brugère, J.-F.; Gribaldo, S. Phylogenomic Data Support a Seventh Order of Methylotrophic Methanogens and Provide Insights into the Evolution of Methanogenesis. *Genome Biol. Evol.* **2013**, *5*, 1769–1780, doi:10.1093/gbe/evt128.
11. Söllinger, A.; Schwab, C.; Weinmaier, T.; Loy, A.; Tveit, A.T.; Schleper, C.; Urich, T. Phylogenetic and genomic analysis of *Methanomassiliicoccales* in wetlands and animal intestinal tracts reveals clade-specific habitat preferences. *FEMS Microbiol. Ecol.* **2016**, *92*, doi:10.1093/femsec/fiv149.
12. Borrel, G.; McCann, A.; Deane, J.; Neto, M.C.; Lynch, D.B.; Brugère, J.-F.; O'Toole, P.W. Genomics and metagenomics of trimethylamine-utilizing *Archaea* in the human gut microbiome. *ISME J.* **2017**, *11*, 2059–2074, doi:10.1038/ismej.2017.72.
13. Lang, K.; Schuldes, J.; Klingl, A.; Poehlein, A.; Daniel, R.; Brune, A. New Mode of Energy Metabolism in the Seventh Order of Methanogens as Revealed by Comparative Genome Analysis of "*Candidatus* Methanoplasma termitum." *Appl. Environ. Microbiol.* **2015**, *81*, 1338–1352, doi:10.1128/AEM.03389-14.
14. Borrel, G.; Parisot, N.; Harris, H.M.; Peyretaillade, E.; Gaci, N.; Tottey, W.; Bardot, O.; Raymann, K.; Gribaldo, S.; Peyret, P.; et al. Comparative genomics highlights the unique biology of *Methanomassiliicoccales*, a *Thermoplasmatales*-related seventh order of methanogenic archaea that encodes pyrrolysine. *BMC Genom.* **2014**, *15*, 679, doi:10.1186/1471-2164-15-679.
15. Zinke, L.A.; Evans, P.N.; Schroeder, A.L.; Parks, D.H.; Varner, R.K.; Rich, V.I.; Tyson, G.W.; Emerson, J.B. Evidence for non-methanogenic metabolisms in globally distributed archaeal clades basal to the *Methanomassiliicoccales*. *Environ. Microbiol.* **2020**, *n/a*, doi:10.1101/2020.03.09.984617.
16. Gascuel, O. BIONJ: An improved version of the NJ algorithm based on a simple model of sequence data. *Mol. Biol. Evol.* **1997**, *14*, 685–695, doi:10.1093/oxfordjournals.molbev.a025808.
17. Kröninger, L.; Berger, S.; Welte, C.; Deppenmeier, U. Evidence for the involvement of two heterodisulfide reductases in the energy-conserving system of *Methanomassiliicoccus luminyensis*. *FEBS J.* **2016**, *283*, 472–483, doi:10.1111/febs.13594.
18. Kemnitz, D.; Kolb, S.; Conrad, R. Phenotypic characterization of Rice Cluster III archaea without prior isolation by applying quantitative polymerase chain reaction to an enrichment culture. *Environ. Microbiol.* **2005**, *7*, 553–565, doi:10.1111/j.1462-2920.2005.00723.x.
19. Kröninger, L.; Gottschling, J.; Deppenmeier, U. Growth Characteristics of *Methanomassiliicoccus luminyensis* and expression of methyltransferase encoding genes. *Archaea* **2017**, 2756573, doi:10.1155/2017/2756573
20. Mah, R.A.; Smith, M.R.; Baresi, L. Studies on an acetate-fermenting strain of *Methanosarcina*. *Appl. Environ. Microbiol.* **1978**, *35*, 1174–1184.
21. Webster, G.; Rinna, J.; Roussel, E.G.; Fry, J.C.; Weightman, A.J.; Parkes, R.J. Prokaryotic functional diversity in different biogeochemical depth zones in tidal sediments of the Severn Estuary, UK, revealed by stable-isotope probing. *FEMS Microbiol. Ecol.* **2010**, *72*, 179–197, doi:10.1111/j.1574-6941.2010.00848.x.
22. Ciobanu, M.-C.; Rabineau, M.; Droz, L.; Révillon, S.; Ghiglione, J.-F.; Dennielou, B.; Jorry, S.-J.; Kallmeyer, J.; Etoubleau, J.; Pignet, P.; et al. Sedimentological imprint on subseafloor microbial communities in Western Mediterranean Sea Quaternary sediments. *Biogeosciences* **2012**, *9*, 3491–3512, doi:10.5194/bg-9-3491-2012.

23. Pierre, C.; Blanc-Valleron, M.-M.; Demange, J.; Boudouma, O.; Foucher, J.-P.; Pape, T.; Himmler, T.; Fekete, N.; Spiess, V. Authigenic carbonates from active methane seeps offshore southwest Africa. *Geo Mar. Lett.* **2012**, *32*, 501–513, doi:10.1007/s00367-012-0295-x.
24. Murakami, T.; Segawa, T.; Takeuchi, N.; Sepúlveda, G.B.; Labarca, P.; Kohshima, S.; Hongoh, Y. Metagenomic analyses highlight the symbiotic association between the glacier stonefly *Andiperla willinki* and its bacterial gut community. *Environ. Microbiol.* **2018**, *20*, 4170–4183, doi:10.1111/1462-2920.14420.
25. Olsen, I. *Dysgonomonas*. In *Bergey's Manual of Systematics of Archaea and Bacteria*; John Wiley & Sons, Inc: Hoboken, NJ, USA, 2015; pp. 1–8, ISBN 978-1-118-96060-8, doi:10.1002/9781118960608.gbm00243.
26. Vartoukian, S.R.; Palmer, R.M.; Wade, W.G. The division "*Synergistes*." *Anaerobe* **2007**, *13*, 99–106, doi:10.1016/j.anaerobe.2007.05.004.
27. Yutin, N.; Galperin, M.Y. A genomic update on clostridial phylogeny: Gram-Negative spore formers and other misplaced clostridia. *Environ. Microbiol.* **2013**, *15*, 2631–2641, doi:10.1111/1462-2920.12173.
28. Goodrich, J.K.; Waters, J.L.; Poole, A.C.; Sutter, J.L.; Koren, O.; Blekhman, R.; Beaumont, M.; Van Treuren, W.; Knight, R.; Bell, J.T.; et al. Human Genetics Shape the Gut Microbiome. *Cell* **2014**, *159*, 789–799, doi:10.1016/j.cell.2014.09.053.
29. Morotomi, M.; Nagai, F.; Watanabe, Y. Description of *Christensenella minuta* gen. nov., sp. nov., isolated from human faeces, which forms a distinct branch in the order *Clostridiales*, and proposal of *Christensenellaceae* fam. nov. *Int. J. Syst. Evol. Microbiol.* **2012**, *62*, 144–149, doi:10.1099/ijs.0.026989-0.
30. Lee, J.; Koo, T.; Yulisa, A.; Hwang, S. Magnetite as an enhancer in methanogenic degradation of volatile fatty acids under ammonia-stressed condition. *J. Environ. Manag.* **2019**, *241*, 418–426, doi:10.1016/j.jenvman.2019.04.038.
31. Waters, J.L.; Ley, R.E. The human gut bacteria *Christensenellaceae* are widespread, heritable, and associated with health. *BMC Biol.* **2019**, *17*, 83, doi:10.1186/s12915-019-0699-4.
32. Graf, J. The Family *Rikenellaceae*. In *The Prokaryotes: Other Major Lineages of Bacteria and The Archaea*; Rosenberg, E., DeLong, E.F., Lory, S., Stackebrandt, E., Thompson, F., Eds.; Springer: Berlin/Heidelberg, Germany, 2014; pp. 857–859, ISBN 978-3-642-38954-2, doi:10.1007/978-3-642-38954-2_134.
33. Liu, P.; Klose, M.; Conrad, R. Temperature effects on structure and function of the methanogenic microbial communities in two paddy soils and one desert soil. *Soil Biol. Biochem.* **2018**, *124*, 236–244, doi:10.1016/j.soilbio.2018.06.024.
34. Schink, B. *Anaerovorax*. In *Bergey's Manual of Systematics of Archaea and Bacteria*; John Wiley & Sons, Inc: Hoboken, NJ, USA, 2015; pp. 1–3, ISBN 978-1-118-96060-8, doi:10.1002/9781118960608.gbm00725.
35. Drake, H.L.; Gössner, A.S. *Sporomusa*. In *Bergey's Manual of Systematics of Archaea and Bacteria*; John Wiley & Sons, Inc: Hoboken, NJ, USA, 2015; pp. 1–7, ISBN 978-1-118-96060-8, doi:10.1002/9781118960608.gbm00706.
36. Fowler, S.J.; Dong, X.; Sensen, C.W.; Suflita, J.M.; Gieg, L.M. Methanogenic toluene metabolism: Community structure and intermediates. *Environ. Microbiol.* **2012**, *14*, 754–764, doi:10.1111/j.1462-2920.2011.02631.x.
37. Liu, X.; Li, M.; Castelle, C.J.; Probst, A.J.; Zhou, Z.; Pan, J.; Liu, Y.; Banfield, J.F.; Gu, J.-D. Insights into the ecology, evolution, and metabolism of the widespread *Woesearchaeotal* lineages. *Microbiome* **2018**, *6*, 102, doi:10.1186/s40168-018-0488-2.
38. Suzuki, D.; Li, Z.; Cui, X.; Zhang, C.; Katayama, A. Reclassification of *Desulfobacterium anilini* as *Desulfatiglans anilini* comb. nov. within *Desulfatiglans* gen. nov., and description of a 4-chlorophenol-degrading sulfate-reducing bacterium, *Desulfatiglans parachlorophenolica* sp. nov. *Int. J. Syst. Evol. Microbiol.* **2014**, *64*, 3081–3086, doi:10.1099/ijs.0.064360-0
39. Galushko, A.; Kuever, J. *Desulfatiglans*. In *Bergey's Manual of Systematics of Archaea and Bacteria*; John Wiley & Sons, Inc: Hoboken, NJ, USA, 2019; pp. 1–4, ISBN 978-1-118-96060-8, doi:10.1002/9781118960608.gbm01679.
40. Deeg, C.M.; Zimmer, M.M.; George, E.E.; Husnik, F.; Keeling, P.J.; Suttle, C.A. *Chromulinavorax destructans*, a pathogen of microzooplankton that provides a window into the enigmatic candidate phylum Dependentiae. *PLoS Pathog.* **2019**, *15*, e1007801, doi:10.1371/journal.ppat.1007801.
41. Snell-Castro, R.; Méndez-Acosta, H.O.; Arreola-Vargas, J.; González-Álvarez, V.; Pintado-González, M.; González-Morales, M.T.; Godon, J.J. Active prokaryotic population dynamics exhibit high correlation to reactor performance during methane production from acid hydrolysates of *Agave tequilana* var. *azul* bagasse. *J. Appl. Microbiol.* **2019**, *126*, 1618–1630, doi:10.1111/jam.14234.
42. Zhang, S.; Chang, J.; Lin, C.; Pan, Y.; Cui, K.; Zhang, X.; Liang, P.; Huang, X. Enhancement of methanogenesis via direct interspecies electron transfer between *Geobacteraceae* and *Methanosaetaceae* conducted by granular activated carbon. *Bioresour. Technol.* **2017**, *245*, 132–137, doi:10.1016/j.biortech.2017.08.111.
43. Zinder, S.; Bräuer, S. *Methanoregula*. In *Bergey's Manual of Systematics of Archaea and Bacteria*; John Wiley & Sons, Inc: Hoboken, NJ, USA, 2016; pp. 1–8, ISBN 978-1-118-96060-8, doi:10.1002/9781118960608.gbm01368
